# *Brevibacillus laterosporus* strains BGSP7, BGSP9 and BGSP11 isolated from silage produce broad spectrum multi-antimicrobials

**DOI:** 10.1371/journal.pone.0216773

**Published:** 2019-05-10

**Authors:** Marija Miljkovic, Sofija Jovanovic, Paula M. O’Connor, Nemanja Mirkovic, Branko Jovcic, Brankica Filipic, Miroslav Dinic, David John Studholme, Djordje Fira, Paul D. Cotter, Milan Kojic

**Affiliations:** 1 Laboratory for Molecular Microbiology, Institute of Molecular Genetics and Genetic Engineering, University of Belgrade, Belgrade, Serbia; 2 Teagasc Food Research Centre, Moorepark, Fermoy, Ireland; 3 APC Microbiome Ireland, Cork, Ireland; 4 Faculty of Biology, University of Belgrade, Belgrade, Serbia; 5 Faculty of Pharmacy, University of Belgrade, Belgrade, Serbia; 6 Biosciences, College of Life and Environmental Sciences, University of Exeter, Exeter, United Kingdom; Institut National de la Recherche Agronomique, FRANCE

## Abstract

Bacteria active against multi-drug resistant pathogens, isolated by direct selection of colonies from clover silage samples, produce zones of inhibition against two Gram-negative (*Klebsiella pneumoniae* Ni9 and *Pseudomonas aeruginosa* MMA83) and two Gram-positive (*Staphylococcus aureus* ATCC25923 and *Listeria monocytogenes* ATCC19111) pathogens. Isolates BGSP7, BGSP9, BGSP11 and BGSP12 produced the largest zones of inhibition against all four pathogens when grown in LB broth with aeration at 37°C. Isolates BGSP7, BGSP9, BGSP11 and BGSP12 were identified as *Brevibacillus laterosporus* and pulsed field gel electrophoresis and extracellular protein profiles showed that three different strains (BGSP7, BGSP9 and BGSP11) were isolated. A semi-native SDS-PAGE (sodium dodecyl sulphate-polyacrylamide gel electrophoresis) gel overlay assay showed that BGSP7 and BGSP9 produce small antimicrobial molecules of about 1.5 kDa, while BGSP11 produces antimicrobial molecules of 1.5 and 6 kDa active against *S*. *aureus* ATCC25923. Amino acid analysis of two antimicrobial molecules (1583.73 Da; from BGSP7 and 1556.31 Da; from BGSP11) revealed that they have a similar composition and differ only by virtue of the presence of a methionine which is present only in BGSP11 molecule. Genome sequencing of the three isolates revealed the presence of gene clusters associated with the production of non-ribosomally synthesized peptides (brevibacillin, bogorol, gramicidin S, plipastatin and tyrocin) and bacteriocins (laterosporulin, a lactococcin 972-like bacteriocin, as well as putative linocin M18, sactipeptide, UviB and lantipeptide-like molecules). Ultimately, the purification of a number of antimicrobial molecules from each isolate suggests that they can be considered as potent biocontrol strains that produce an arsenal of antimicrobial molecules active against Gram-positive and Gram-negative multi-resistant pathogens, fungi and insects.

## Introduction

The acquisition and spread of antibiotic resistance among pathogenic bacteria poses a great threat to public health, especially in light of the paucity of new antibiotics being developed [1]. Consequently, there is a need for new antimicrobials that can be used as alternatives to conventional antibiotics. Antimicrobial peptides and bacteriocins are considered as such potential alternatives [2, 3]. Bacteriocins are ribosomally synthesized peptides with antimicrobial activity produced by many bacterial species. In most cases, bacteriocins inhibit the growth of closely related bacteria, although some exhibit a broad inhibitory spectrum against food-spoilage and pathogenic bacteria [4]. The vast majority of these molecules are initially synthesized intracellularly as larger inactive precursors, and many of them are further subjected to different enzymatic modifications, such as cyclization, glycosylation and the introduction of lanthionine and beta-methyl lanthionine [5]. According to the latest classification scheme, bacteriocins can be divided into three main groups [2, 6]. Class I are represented by antimicrobial peptides that have been enzymatically modified; Class II, which encompasses non-modified or minimally modified peptides, that are further divided into several subgroups; Class III, containing the large, unmodified heat sensitive antimicrobial proteins with bacteriolytic or some other mechanism of action.

Bacteria from the genus *Bacillus* and related genera produce many substances with antimicrobial activity, including non-ribosomally synthesized peptides and lipopeptides [7–9], polyketide compounds [10] and bacteriocins. Indeed, *Bacillus* strains produce bacteriocins from all three classes [11]. Class I representatives are subtilin, ericin A, ericin S, mersacidin, paenibacillin, sublancin 168 [11, 12], haloduracin, lichenicidin and subtilosin A [13–15]. Class II bacteriocins found in *Bacillus* strains include coagulin, produced by *Bacillus coagulans* [16], lichenocin 50.2, produced by *B*. *licheniformis* VPS50.2 [17] and others [18]. In *Bacillus*, class III is represented by large proteins that have enzymatic activity, such as the megacin family of bacteriocins produced by strains of *Bacillus megaterium* [18–20].

Antagonistic compounds produced by bacteria from the genus *Brevibacillus* have also been studied in recent years [21] and strains of *Brevibacillus laterosporus* are well known producers of antibacterial and antifungal agents [22–24]. They are considered an important tool for biological control because of their biopesticidal properties against insects and nematodes [25, 26]. The recently characterized *Br*. *laterosporus* OSY-I_1_ produces brevibacillin, a 1583 Da antimicrobial lipopeptide with a linear structure containing 13 amino acids and a C_6_ fatty acid at the N-terminus [27]. Brevibacillin shows strong antimicrobial activity against some pathogenic and food-spoilage Gram-positive bacteria, particularly methicillin resistant *Staphylococcus aureus*, *Listeria monocytogenes* and *Bacillus cereus*. The mechanism of action of this molecule is most likely based on its amphiphilic nature and the ability of the cationic amino acids to interact with the negatively charged phospholipids of the cell membrane, causing the disruption and depolarization of the membrane [27]. A similar mechanism was observed in the case of paenibacterin, a broad-spectrum antimicrobial lipopeptide produced by *Paenibacillus thiaminolyticus* [28]. In addition, it was found that the marine bacterial isolate *Brevibacillus laterosporus* PNG-276 showed broad-spectrum antibiotic activity (producing polyketides the basiliskamides A and B and nonribosomal peptides: loloatins A-D and bogorols A-E) against the human pathogens MRSA, VRE, *Mycobacterium tuberculosis*, *Candida albicans*, and *Escherichia coli* [29].

From a bacteriocin perspective, laterosporulin is an example of a class IId bacteriocin produced by strain GI-9, preliminarily identified as *Br*. *laterosporus* [30]. Laterosporulin is active against both Gram-positive and Gram-negative bacteria and was found to be resistant to a range of proteolytic enzymes. Structural studies revealed that the peptide consists of twisted β-sheet and includes three disulfide bonds [31]. Laterosporulin is relatively rich in cysteine and polar amino acids, which is atypical for bacteriocins in general, whereas its structure showed similarities with mammalian defensins. More recently, laterosporulin 10, produced by the strain *Brevibacillus* sp. SKDU10 was characterized [32] and, while considered similar, shows only 57.6% identity with laterosporulin. In addition, this novel bacteriocin has a different antimicrobial spectrum to laterosporulin as activity is limited to Gram-positive bacteria. However, laterosporulin 10 has also proven to be a promising new anti-cancer molecule that exhibits a cytotoxic effect on cancer cells [33].

A number of *Br*. *laterosporus* strains have been sequenced and their genomes deposited in Genbank. These include LMG 15441 (under accession number AFRV00000000, [34]), GI-9 (under accession numbers CAGD01000001 to CAGD01000061, [35]), B9 (under accession numbers CP011074-CP011076, [36]), Lak 1210 (under accession number NDIP00000000, [37]), OSY-I_1_ (under accession number NOLX00000000, [38]), DSM25 (accession number ARFS00000000), ZQ2, UBA5783, DSM25, PE36 (accession number NZ_AXBT00000000.1) and Uniss_18.

The current study focused on the isolation of new cultivable bacteria that produce natural antimicrobial molecules effective against multidrug resistant pathogens of humans, animals and plants, regardless of their taxonomy. This approach lead to the characterization and determination of the antimicrobial activity of three new isolated strains of *Br*. *laterosporus* from silage in order to determine their potential as biocontrol agents.

## Materials and methods

### Bacterial strains and culture conditions

Strains used in this study are listed in Table 1. *Achromobacter*, *Acinetobacter*, *Burkholderia*, *Erwinia*, *Escherichia*, *Klebsiella*, *Pseudomonas*, *Salmonella* and *Staphylococcus* strains were grown in Luria Bertani (LB) medium at 37°C with aeration, while *Agrobacterium*, *Bacillus*, *Chromobacterium*, *Ralstonia* and *Xanthomonas* strains were grown in the same medium at 30°C with aeration. *Enterococcus*, *Lactococcus*, and *Listeria* strains were grown in M17 medium (Merck GmbH, Darmstadt, Germany) supplemented with D-glucose (0.5% w/v) (GM17) at 30°C. *Lactobacillus* strains were grown in MRS medium (Merck GmbH, Darmstadt, Germany) at 30°C. *Streptococcus* strains were grown in Brain Heart Infusion (BHI) medium (Oxoid, Basingstoke, Hampshire, England) at 37°C and an atmosphere of 5% CO_2_. *Paenibacillus larvae* was grown in Mueller Hinton broth (Oxoid, Basingstoke, Hampshire, England) supplemented with yeast extract (1.5% w/v), K_2_HPO_4_ (0.3% w/v), D-glucose (0.2% w/n), sodium pyruvate (0.1% w/n) and 0.1 mg/l of thiamine at 30°C. The bioactive compounds-producing isolates, *Br*. *laterosporus* BGSP7, BGSP9, BGSP11 and BGSP12 were isolated from a sample of clover silage, taken from a household in a village Stari Tamiš, near Pančevo, Serbia. Optimal growth conditions of *Br*. *laterosporus* BGSP7, BGSP9 and BGSP11 strains (medium, optimal temperature, aeration), were determined by growing a 1% inoculum of overnight culture (small-scale 5 ml) in different growth media (LB, minimal medium (M9) with glucose, BHI, GM17 and MRS) at different temperatures and aeration by monitoring the absorbance OD_600_ at every hour of growth. For surface sliding mobility assays, minimal medium (MSgg) as described previously by Branda et al. [39] was used. Solid medium and soft agar were made by adding 1.5% or 0.7% (w/v) agar (Torlak, Belgrade, Serbia) to the liquid media, respectively. The following antibiotic concentrations were used: erythromycin, 10 μg/ml (lactococci and *B*. *subtilis* 168) and 300 μg/ml (*E*. *coli*); tetracycline, 20 μg/ml (*E*. *coli*); and ampicillin, 100 μg/ml (*E*. *coli*). The 5-bromo-4-chloro-3-indolyl-β-D-galacto-pyranoside (X-Gal) (Fermentas, Vilnius, Lithuania) was added to LB medium plates for blue/white color screening of colonies with cloned fragments at a final concentration 40 μg/ml (*E*. *coli*).

**Table 1:** Bacterial strains used in this study.

Strains, plasmids and derivatives	Source or reference
*Achromobacter xylosoxidans* MS4	[40]
*Acinetobacter baumannii* 6077/12	[41]
*Agrobacterium tumefaciens*	LMM collection
*Bacillus cereus* ATCC11778	ATCC collection
*Bacillus pumilis* BGSP1-2	This study
*Bacillus pumilis* BGSP2-2	This study
*Bacillus pumilis* BGSP3-1	This study
*Bacillus pumilis* BGSP3-3	This study
*Bacillus pumilis* BGSP5-2	This study
*Bacillus subtilis* 168	[42]
*Brevibacillus laterosporus* BGSP7	This study
*Brevibacillus laterosporus* BGSP9	This study
*Brevibacillus laterosporus* BGSP11	This study
*Brevibacillus laterosporus* BGSP12	This study
*Burkholderia cepacia*	[42]
*Burkholderia glumae*	[42]
*Chromobacterium violaceum*	LMM collection
*Enterococcus faecalis* ZLS10-27	[43]
*Erwinia carotovora*	[42]
*Escherichia coli* ATCC25922	ATCC collection
*Escherichia coli* DH5α	[44]
*Escherichia coli* HB101	[45]
*Klebsiella pneumoniae* Ni9	[46]
*Lactobacillus paracasei* BGSJ2-8	[47]
*Lactobacillus plantarum* 9208	[48]
*Lactobacillus zeae*	[48]
*Lactococcus cremoris* MG7284	[49]
*Lactococcus lactis* BGBU1-4	[50]
*Lactococcus lactis* HP	Laboratory collection
*Lactococcus raffinolactis* BGTRK10-1	[51]
*Listeria monocytogenes* ATCC19111	ATCC collection
*Paenibacillus larvae*	Faculty of Veterinary Medicine, University of Belgrade, Serbia
*Pseudomonas aeruginosa* MMA83	[52]
*Pseudomonas syringae*	LMM collection
*Ralstonia pickettii* 666	LMM collection
*Salmonella enteritidis* ATCC13706	ATCC collection
*Staphylococcus aureus* ATCC25923	ATCC collection
*Streptococcus agalactiae* B165	Laboratory “Paster”, Belgrade, Serbia
*Streptococcus mutans* BGSF1	[53]
*Streptococcus pneumoniae* P173	Laboratory “Paster”, Belgrade, Serbia
*Streptococcus pyogenes* A2941	Laboratory “Paster”, Belgrade, Serbia
*Streptococcus thermophilus*	LMM collection
*Xanthomonas oryzae*	LMM collection

ATCC—American Type Culture Collection;

LMM collection—Collection of Laboratory for Molecular Microbiology, Institute of Molecular Genetics and Genetic Engineering, University of Belgrade, Serbia.

### Sample collection, screening and isolation of producers of antimicrobial molecules

Samples of soil (5), silage (2) and fermented vegetables (13) were collected for the isolation of bacterial producers of antimicrobials active against pathogens. One gram of each sample was suspended in 50 ml of physiological solution (8.9 g/l NaCl) and incubated at room temperature in an orbital shaker at 200 rpm for 30 min. Mixtures were allowed to settle and serial dilutions up to 10^−5^ were prepared using sterile physiological solution. Isolation of bacteria from these mixtures was done by spreading serial dilutions of each sample on five different growth media in quadruplicate (LA, M9A, BHIA, GM17A and MRSA) and plates incubated at 30 and 37°C for 48 hours. The Petri dishes with grown colonies (50–300) were overlaid with soft agar containing four different multi-drug resistant pathogenic indicator strains (two Gram-negative pathogens: *K*. *pneumoniae* Ni9, *P*. *aeruginosa* MMA83 and two Gram-positive pathogens: *S*. *aureus* ATCC25923, *L*. *monocytogenes* ATCC19111) and plates incubated overnight at the appropriate temperatures. Colonies producing antimicrobial molecules were detected by the appearance of a zone of inhibition around the colonies. Purification of antimicrobial producers was achieved by repeated streaking of single colonies from the center of inhibition zones on the medium from which it was originally selected. The ability of the purified isolates to produce inhibitory molecules and their spectrum of activity was tested by the agar well diffusion assay as described previously [54]. Briefly, 50 μl aliquots of sample (culture or filtered supernatant) were assayed in wells pre-made in soft agar, and indicator plates were incubated under appropriate conditions for the respective indicator strain. A clear zone of inhibition around the wells following 24h of incubation was taken as evidence of antimicrobial production. To monitor the kinetics of antimicrobial production/activity 100 ml of fresh pre-warmed LB broth was inoculated with overnight culture (1% v/v) and incubated at 37°C with aeration. Samples were taken every hour from 0 to 16h and at 24h for determination of OD_600_ and antimicrobial activity by the agar well diffusion assay (using cell free supernatants of cultures) where *S*. *aureus* ATCC25923 and *P*. *aeruginosa* MMA83 were used as indicator strains. To confirm the proteinaceous nature of antimicrobial molecules, a crystal of the proteolytic enzyme, pronase E, (Sigma, St. Louis, MO, USA) was placed close to the edge of the potential antimicrobial compound containing well and reduction of activity was taken as confirmation.

Molecular masses of bioactive peptides were estimated by Tricine-SDS-PAGE (sodium dodecyl sulphate-polyacrylamide gel electrophoresis) 15% acrylamide gels [55]. The position of the active peptides was determined by comparing the position of the zone inhibition (on overlaid part of the gel with LB soft agar containing indicator strain *S*. *aureus* and incubated overnight at 37°C) with protein bands on the part of the stained gel with Coomassie brilliant blue R-250.

### Identification of isolates with antimicrobial activity

Identification of selected isolates was done by testing their morphological and biochemical characteristics. Taxonomic determination of isolates was done initially by 16S rRNA gene sequencing [56], and confirmed by genome sequencing. Platinum *Taq* DNA Polymerase High Fidelity (Thermo Fisher Scientific, Waltham, MA, USA) was used to amplify the gene for 16S rRNA using a GeneAmp PCR System 2700 thermal cycler (Applied Biosystems, Foster City, CA, USA) under the conditions listed in S1A Table. PCR products were checked on a 1% agarose gel (at a constant voltage of 80 V) and purified using a Thermo Scientific PCR Purification Kit (Thermo Scientific, Lithuania) according to the manufacturer’s protocol and sequenced and identified by using BLAST.

Pulse field gel electrophoresis (PFGE) was performed for isolates comparison and for strain determination, as described previously [57] using *Not*I macro-restriction polymorphism.

### Effect of temperature, pH and enzymes on antimicrobial activity

Antimicrobial molecules concentrated from culture supernatant using 50% ammonium sulphate saturation were tested for: thermal stability by incubation at 60, 80, 100 and 121°C for 15 and 30 min; for pH resistance by adjusting pH from 2–12 and incubating for 1 h at 37°C followed by neutralisation to pH 7 for 30 min at room temperature; for resistance to different enzymes (trypsin, pepsin, α-chymotrypsin, proteinase K, pronase E, lysozyme, lipase and α-amylase) samples of antimicrobial molecules were incubated in buffered conditions and temperature appropriate for enzymes for 1h and 24h as described previously [54]. After treatments, antimicrobial activity was determined by agar well diffusion assay, as described above using *S*. *aureus* ATCC25923 and *L*. *monocytogenes* ATCC19111 indicator strains; untreated bacteriocin samples were used as a control. All experiments were done in triplicate.

The potency of purified bacteriocin like inhibitory substances (stored at 4, 37 and 45°C) were checked at regular intervals 0, 1, 2, 3, 4, 5, 6 and 12 months against sensitive indicators by the agar well diffusion method.

### Purification of antimicrobial molecules produced by *Br*. *laterosporus* strains and partial characterisation by amino acid analysis

One liter aliquots of BGSP7, BGSP9 and BGSP11 were grown overnight in LB broth which had been passed through a column containing Amberlite XAD 16 to remove hydrophobic peptides which can interfere with purification. Cultures were centrifuged (8000 rpm, 10°C, 20 min) and antimicrobial molecules purified from both cells (i) and supernatant (ii); (i) the cell pellet was mixed with 200 ml of 70% propan-2-ol 0.1% TFA (IPA) and stirred for 3–4 h at RT. The resulting suspension was centrifuged (8000 rpm, 10°C, 20 min) and cell supernatant retained for purification. The IPA was removed from the cell extract and it was applied to a 5g, 20 ml Strata-E C18 SPE column (Phenomenex, Cheshire, UK) pre-equilibrated with methanol and distilled water. The column was washed with 30 ml of 30% ethanol followed by 30 ml of 70% IPA. Eluents were assayed for antimicrobial activity by agar well diffusion using *Lactococcus lactis* HP as the indicator strain. In next step, the IPA was removed from the active eluent and it was applied to a semi prep C12 Proteo column running a 25–50% gradient where buffer A was 0.1% TFA and buffer B 100% acetonitrile 0.1% TFA. Eluent was monitored at 214 nm and fractions were collected at 1 min intervals. Fractions were assayed on *L*. *lactis* HP indicator plates; (ii) the culture supernatant was passed through an Econo column containing 30g Amberlite XAD16 beads pre-washed with 300 ml of distilled water. The column was washed with 300 ml of 35% ethanol and the inhibitory activity eluted in 300 ml of 70% IPA and retained. The IPA was removed from the XAD IPA eluent and it was further purified by C18 SPE and Reversed Phase HPLC as described for the cell extract.

Total amino acids analysis of the 1583.73 Da antimicrobial molecule purified from BGSP7 cells and the 1556.31 Da antimicrobial molecule purified from BGSP11 supernatant were determined using a Biochrom 30+ Automatic Amino Acid Analyzer (Biochrom, Cambridge, UK) provided by Institute of Food Technology, University of Novi Sad, Serbia. Hydrolysis of purified antimicrobial molecules with 5 N HCl and preparation of samples for amino acid composition analysis were performed as recommended by manufacturer (Biochrom, Cambridge, UK).

### Determination of minimum inhibitory concentrations (MICs) for the two most abundant antimicrobial molecules

Micro-dilution assays were done with the 1583.73 Da antimicrobial molecule from BGSP7 and the 1556.31 Da molecule from BGSP11 against indicator organisms (listed in Table 1) as described previously [58]. All indicator strains were diluted to 0.5 McFarland units from which 20 μl were distributed in wells of a clear 96-well flat bottom microtiter plate. Antimicrobial molecules from BGSP7 and BGSP11 (resuspended at 1 mg/ml in water) were two-fold serially diluted to give a dilution series from 250 μg/ml to 7.8 μg/ml. Medium (blanks) and untreated culture were included as controls. The microtiter plates were incubated at 37°C for 24 h, and the optical densities at 595 nm (OD_595_) recorded (Infinite M200pro, Tecan, Switzerland). All experiments were performed in triplicate.

### DNA manipulations

Genomic DNA was extracted by the method described previously [59] with minor modifications: logarithmic phase cells were pre-treated with lysozyme (4 mg/ml, for 15 min at 37°C) prior to treatment with 2% SDS. Plasmids from *Br*. *laterosporus*, *B*. *subtilis* and *L*. *lactis* were isolated by the method described by O’Sullivan and Klaenhammer [60]. For plasmid isolation from *E*. *coli*, a Thermo Fisher Scientific GeneJET Plasmid Miniprep kit was used according to the manufacturer’s recommendations (Thermo Scientific, Lithuania). Digestion with restriction enzymes was conducted according to the supplier’s instructions (Thermo Fisher Scientific Waltham, MA, USA). The DNA fragments from agarose gels were purified using QIAqick Gel extraction kit as described by the manufacturer (Qiagen, Hilden, Germany). DNA was ligated with T4 DNA ligase (Agilent technologies, USA) according to the manufacturer’s recommendations. Standard heat-shock transformation was used for plasmid transformation of *E*. *coli* [44]. *L*. *lactis* subsp. *cremoris* MG7284 was transformed with plasmid constructs by electroporation using a method described by Holo and Nes [61] with modifications specified in [62]. *Bacillus subtilis* 168 was transformed by plasmid DNA constructs after induction of competence by the method described by Bennallack et al. [63].

### Real time quantitative PCR (RT-qPCR)

Total RNA was isolated from bacterial cells during different growth phases of *Br*. *laterosporus* BGSP7, BGSP9 and BGSP11 using the RNeasy Mini kit (Qiagen). The residual DNA was digested using an Ambion DNA free Kit (Thermo Fisher Scientific, MA, USA). Isolated RNA was quantified using a NanoDrop spectrophotometer (GE Healthcare, Life Science) and integrity was analyzed on a 1.2% formaldehyde-agarose gel. The first-strand of cDNA was synthesized with a RevertAid RT Reverse Transcription Kit according to the enzyme manufacturer instructions (Thermo Fisher Scientific, MA, USA), using 1 μg of isolated RNA as a template. Random hexamers (Applied Biosystems) and RiboLock RNase inhibitor (Thermo Scientific) were used in the reactions. The qPCR was carried out using the KAPA SYBR Fast qPCR Kit (KAPA Biosystems, MA, USA) in a 7500 Fast Real-Time PCR System (Applied Biosystems) under the conditions and appropriate pairs of primers listed in S1C Table. The results were normalized to the reference *rpoD* gene. The obtained Ct values from log phase of bacterial growth were set as calibrators and all results were expressed as relative target abundance using the 2^-ΔΔCt^ method [64].

### Construction of a cosmid library and screening for clones carrying bacteriocin operons

Total genomic DNA isolated from BGSP7, BGSP9 and BGSP11 was partially digested with *Eco*RI restriction enzyme. Digestion was carried out at room temperature (ca. 23°C) and, during incubation, samples were collected at different time points and EDTA immediately added to a final concentration 10 mM (pH 8) to stop digestion. pLAFR3 cosmid vector was used for construction and the cosmid library was digested with the same restriction enzyme, dephosphorylated and ligated with partially digested total DNA. Ligation was checked for formed concatemers of high molecular weight on 1% agarose gel electrophoresis (at a constant voltage of 80 V) before encapsulation into phage particles using a packaging kit (Agilent technologies). Encapsulated cosmids were transfected into *E*. *coli* HB101 cells prepared in 10 mM MgSO_4_. Clones were selected on LA plates containing tetracycline 20μg/ml.

Cosmid libraries were screened for cosmids carrying cloned bacteriocin gene clusters for laterosporulin and lactococcin 972-like bacteriocins using colony PCR under the conditions listed in S1B Table. Specific sets of primers for laterosporulin and lactococcin 972-like bacteriocin genes were used; one pair of primers for laterosporulin and second pair for lactococcin 972-like bacteriocin genes from all three strains (BGSP7, BGSP9 and BGSP11) were designed according to comparative analysis of their sequences. For PCR analysis from each cosmid library (BGSP7, BGSP9 and BGSP11) 550 colonies were randomly chosen. The size of the fragments cloned into the cosmid vector was analysed on 1% agarose gels (at a constant voltage of 80 V), after digestion with *Eco*RI to compare the presence of common fragments. Positively selected colonies (pcosLS7, pcosLS9, pcosLS11, pcosLC7, pcosLC9, and pcosLC11) were further tested after DNA isolation using the QIAprep Spin Miniprep kit (Qiagen GmBH, Hilden, Germany) to possess the entire gene cluster for the corresponding bacteriocin by the PCR method. From restriction enzyme analysis of genome sequences using DNA Strider3 program it was found that the entire gene clusters for both bacteriocins are located within the *Eco*RI restriction fragments of different lengths (laterosporulin: BGSP7-6738 bp, BGSP9-6739 bp, BGSP11-6750 bp; lactococcin 972-like bacteriocin: BGSP7-8532 bp, BGSP9-5749 bp, BGSP11-7217 bp). Corresponding *Eco*RI restriction fragments were subcloned to pAZIL (giving corresponding constructs named as pAZIL-ELS7, pAZIL-ELS9, pAZIL-ELS11, pAZIL-ELC7, pAZIL-ELC9 and pAZIL-ELC11) and pA13 vectors (giving corresponding constructs named as pA13-ELS7, pA13-ELS9, pA13-ELS11, pA13-ELC7, pA13-ELC9 and pA13-ELC11) that were confirmed by sequencing and stored for further experiments.

### DNA sequencing and sequence analysis

Amplified fragments and constructs were sequenced by Macrogen sequencing service (Macrogen Europe, Amsterdam, Netherlands). Sequence annotation and a database search for sequence similarities were completed using the BLAST program of the National Center for Biotechnology Information—NCBI [65]. The DNA Strider3 program was used for open reading frame (ORF) and restriction enzyme prediction.

### Complete genome sequencing and annotation

The genome sequences of *Br*. *laterosporus* BGSP7, BGSP9 and BGSP11 were determined using the Illumina HiSeq platform by MicrobesNG service (MicrobesNG, IMI-School of Biosciences, University of Birmingham, Birmingham, UK). Draft genome sequences of *Br*. *laterosporus* BGSP7, BGSP9 and BGSP11 have been deposited in GenBank under accession numbers: GCA_002927075.1, GCA_002927085.1 and GCA_002926995.1, respectively.

## Results

### Isolation of strains with antimicrobial activity

**Fig 1:**
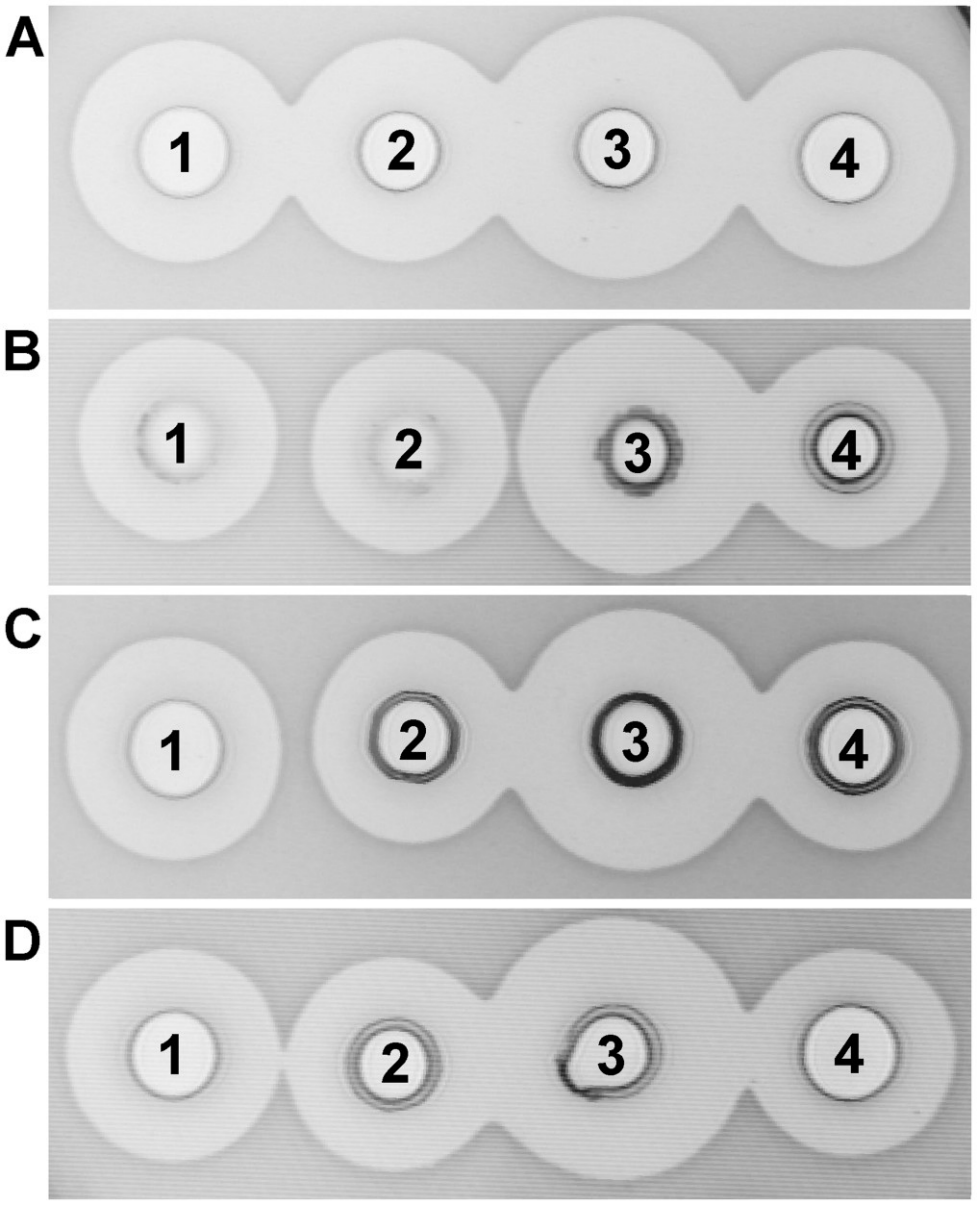
Antimicrobial activity of *Br*. *Laterosporus* BGSP7 (1), BGSP9 (2), BGSP11 (3) and BGSP12 (4) strains on different indicator strains: *S*. *aureus* ATCC25923 (A); *L*. *monocytogenes* ATCC19111 (B); *P*. *aeruginosa* MMA83 (C); *K*. *pneumoniae* Ni9 (D). Antimicrobial activity was analysed after overnight growth (16 h) of the indicator strains.

From approximately 20,000 colonies isolated from samples of soil, clover and corn silage and fermented vegetables, 22 colonies were chosen as potential producers of antimicrobial molecules active against at least one of four tested multi-drug resistant pathogenic strains. It was found that cultures and cell free supernatant of four isolates (BGSP7, BGSP9, BGSP11 and BGSP12) from clover silage showed the strongest inhibitory activity against all four indicator strains, producing zones of inhibition of 20 mm (Fig 1). A further 5 isolates showed inhibitory effects against *S*. *aureus* ATCC25923 and *L*. *monocytogenes* ATCC19111 only with zones of inhibition of 15 mm, while 13 isolates did not show inhibition in the repeated antimicrobial test.

**Fig 2:**
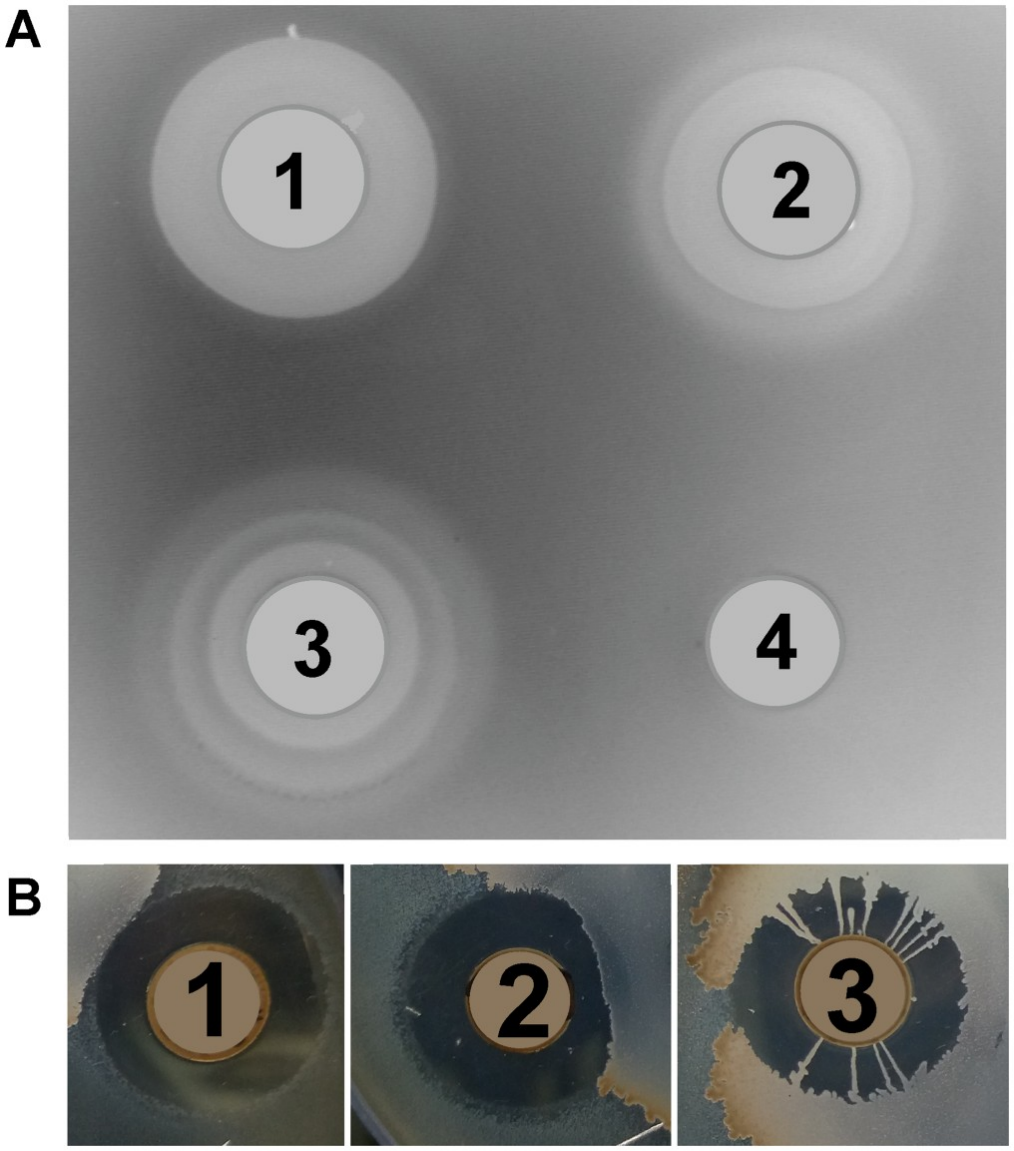
Antimicrobial activity of *Br*. *laterosporus* BGSP7 (1), BGSP9 (2) and BGSP11 (3) strains on *S*. *aureus* ATCC25923 incubated for three days (A); Development of specific structures to over-bridge zone of inhibition by *Br*. *laterosporus* BGSP11 strain in antimicrobial assay with *Br*. *laterosporus* strain BGSP7 (1), BGAP9 (2) and BGSP11 (3) on sensitive strain *S*. *aureus* ATCC25923. Negative control (4) used in antimicrobial test: *S*. *aureus* ATCC25923. Parts showing the antimicrobial activity of the strains were taken from the same Petri dish that was incubated for five days (B).

Isolate BGSP11 produced the largest zones of inhibition against all tested indicator strains (Fig 1). Interestingly, after three days, BGSP9 and especially BGSP11 showed antimicrobial activity in waves of concentric circles of different size. BGSP7 does not display this behavior (Fig 2A). This may indicate that antimicrobial molecules from BGSP9 and BGSP11 strains are active against non-dividing cells of *S*. *aureus* ATCC25923 during stationary phase or successively synthesize various antimicrobial molecules exhibiting different diffusion capacities through agar. In addition, strain BGSP11 forms radial bridges consisting of tendril-like fibers (Fig 2B). Since these strains, especially BGSP11, showed motility, their ability to move on a semi-solid surface was tested. Previously it has been demonstrated that *B*. *subtilis* can use sliding motility to colonize surfaces, using a tendril-like fiber-based growth mode when various macronutrients or micronutrients are limiting [66]. We tested the ability of isolated strains to move on semi-solid medium and found that surface colonization on defined semi-solid medium is dependent on potassium ion and agarose concentration. At low KCl concentrations, strain BGSP7 showed the strongest swarming motility, swarming over the surface with a more dendritic central colony and produced a less robust surface film. Strain BGSP9 showed slower swarming movement on the surface at low KCl concentration but formed a robust surface film compared to strain BGSP7. Strain BGSP11 swarmed over the surface forming a robust film when the concentration of KCl is high, while on medium with low KCl concentrations forms a very weak film without tendril-like fibers (S1 Fig).

### Identification of antimicrobial producing isolates

Phenotypic characterization and BLAST analysis of 16S rRNA gene sequence of nine antimicrobial producing isolates resulted in them being assigned to one of two groups: a) four isolates/strains (BGSP7, BGSP9, BGSP11 and BGSP12) with strong antimicrobial activity against all tested pathogens were classified as *Br*. *laterosporus* species (showing 98–99% identity with *Br*. *laterosporus* strains NRRL-B-14295, HS-A-465 and CSS8); b) the other 5 isolates (BGSP1-2, BGSP2-2, BGSP3-1, BGSP3-3 and BGSP5-2) with moderate antimicrobial activity were classified as *Bacillus pumilus* species (showing 97% identity with *B*. *pumilus* strains X3, HN-30 and S10). The *Br*. *laterosporus* strains were selected for further characterization as they showed much stronger and wider antimicrobial activity compared to the *B*. *pumilus* strains. Optimal growth and production of inhibitory molecules for all four *Br*. *laterosporus* strains/isolates was observed at 37°C in LB medium with aeration. Plasmid isolation revealed that all four isolates contain small plasmids of similar size between 8 and 10 kb; isolates BGSP9 and BGS12 showed the same plasmid profile (S2 Fig).

**Fig 3:**
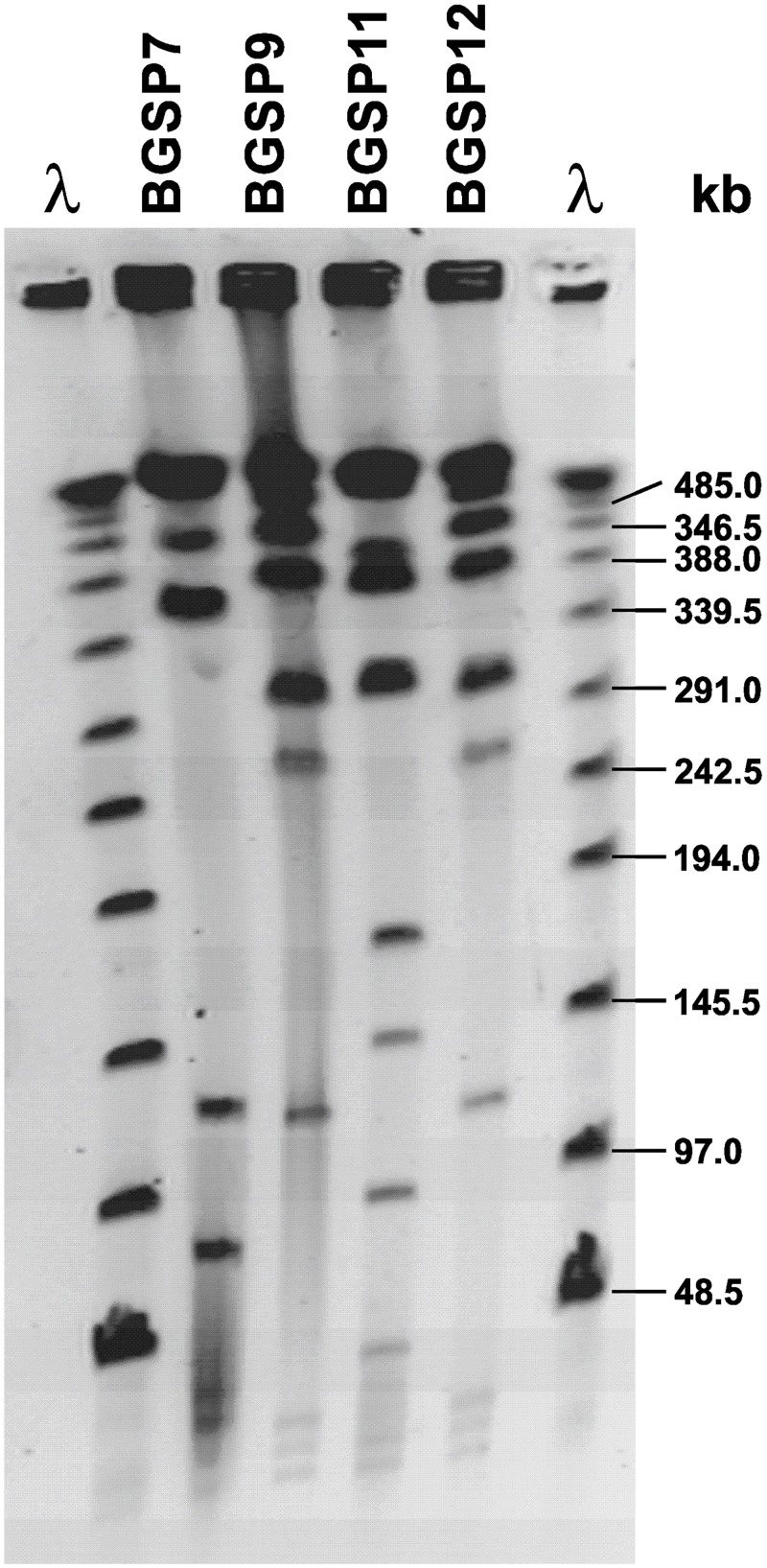
Macrorestriction pattern analysis by pulsed field gel electrophoresis (PFGE) of *Br*. *laterosporus* BGSP7, BGSP9, BGSP11 and BGSP12 strains using *Not*I restriction enzyme on 1.2% agarose gel.

As all four *Br*. *laterosporus* strains/isolates (BGSP7, BGSP9, BGSP11 and BGSP12) showed similar PFGE genotypes. Ultimately, among the four isolates, three distinct strains (BGSP7, BGSP9 and BGSP11) were found to be present, as the BGSP9 and BGSP12 isolates showed identical genomic DNA restriction (*Not*I) profiles (Fig 3).

### Spectrum of activity of antimicrobial molecules produced by *Br*. *laterosporus* BGSP7, BGSP9 and BGSP11 strains

The antimicrobial activity of overnight culture and crude extract from cell free supernatant (50% saturation ammonium sulfate precipitate) was tested against various Gram-positive and Gram-negative spoilage and pathogenic bacteria (listed in Table 1). Both, culture and crude extract from supernatant of isolated strains (BGSP7, BGSP9 and BGSP11) significantly inhibited the growth of all bacteria tested with the exception of *Achromobacter xylosoxidans* MS4, indicating high inhibitory potential and a wide host range. Particularly strong antimicrobial activity was exhibited against *P*. *larvae*, an organism that causes American foulbrood (AFB), a destructive disease of honey bee colonies (S3 Fig).

### Kinetics of antimicrobial production of *Br*. *laterosporus* BGSP7, BGSP9 and BGSP11 strains

**Fig 4:**
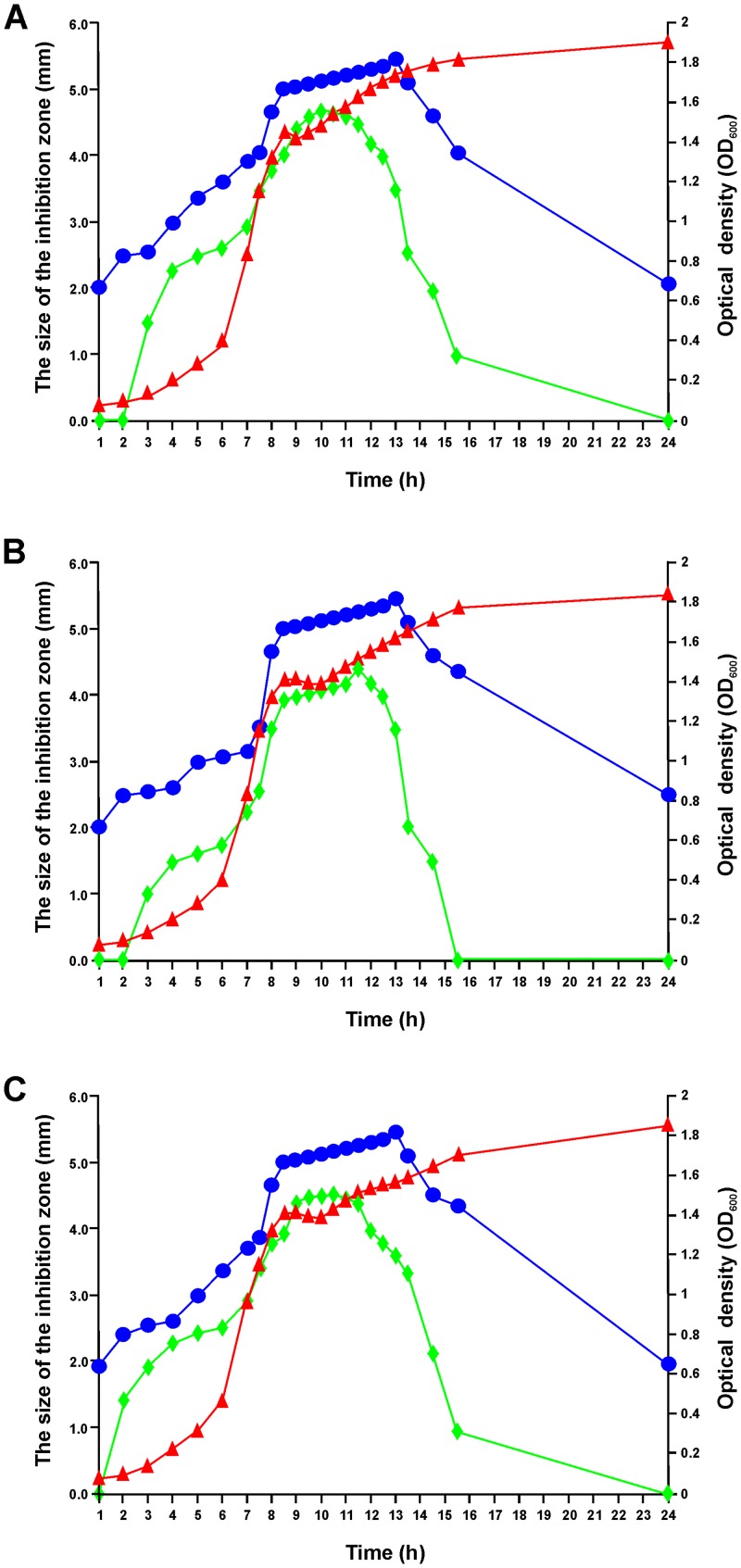
Kinetics of antimicrobial production of *Br*. *laterosorus* BGSP7 (A), BGSP9 (B) and BGSP11 (C) strains during the growth in LB medium at 37°C. Growth of analyzed strains was monitored by measuring optical density at OD_600_ (▲); Synthesis of antimicrobials was quantified by growth inhibition of *S*. *aureus* ATCC25923 (●) and *P*. *aeruginosa* MMA83 (◆) by producer strains measured in millimeters of inhibition zone (mm).

It was found that antimicrobial production depends on the growth phase of strains BGSP7, BGSP9 and BGSP11 (Fig 4). Inhibition of *S*. *aureus* ATCC25923 was achieved by components produced after 1h of growth by all three strains, while production of the components that are inhibitory for *P*. *aeruginosa* MMA83 begins after between 2 and 3h. Thereafter, the antimicrobial bioactivity of the BGSP7, BGSP9 and BGSP11 cultures increases against both indicator strains, which correlates with increasing cell numbers in the logarithmic growth phase. The production of antimicrobial components plateaus between 8 and 13h, which corresponds from late logarithmic to early stationary phase. The antimicrobial bioactivity of BGSP7, BGSP9 and BGSP11 strains on *S*. *aureus* ATCC25923 begins to decline after 13h, but at 24h it is still effective. In contrast, the antimicrobial activity of BGSP7, BGSP9 and BGSP11 strains against *P*. *aeruginosa* MMA83 rapidly decreases after 12h and at 16h has completely disappeared in strain BGSP9, while there is still a low level of activity in BGSP7 and BGSP11.

### Heat, pH, enzymatic and storage stability of crude extract from cell free supernatant

Crude antimicrobial extracts of all three strains showed similar characteristics, i.e., a) high heat stability at 60, 80, 100, and 121°C for 20 min, although treatment at 121°C resulted in a smaller zone of inhibition; b) sensitivity to proteolytic enzymes as evidenced by loss of antimicrobial activity following pronase E treatment for 24 hours, partial reduction by trypsin, pepsin, α-chymotrypsin and proteinase K and insensitivity to lysozyme, lipase and α-amylase confirmed the proteinaceous nature of antimicrobials; c) stability over a wide pH range (2–14); d) antimicrobial activity was stable when stored for one year at 4°C, while at 37°C activity was lost after three months, and at 45°C was lost after one month.

### SDS PAGE analysis of antimicrobial peptides produced by *Br*. *laterosporus* BGSP7, BGSP9 and BGSP11

**Fig 5:**
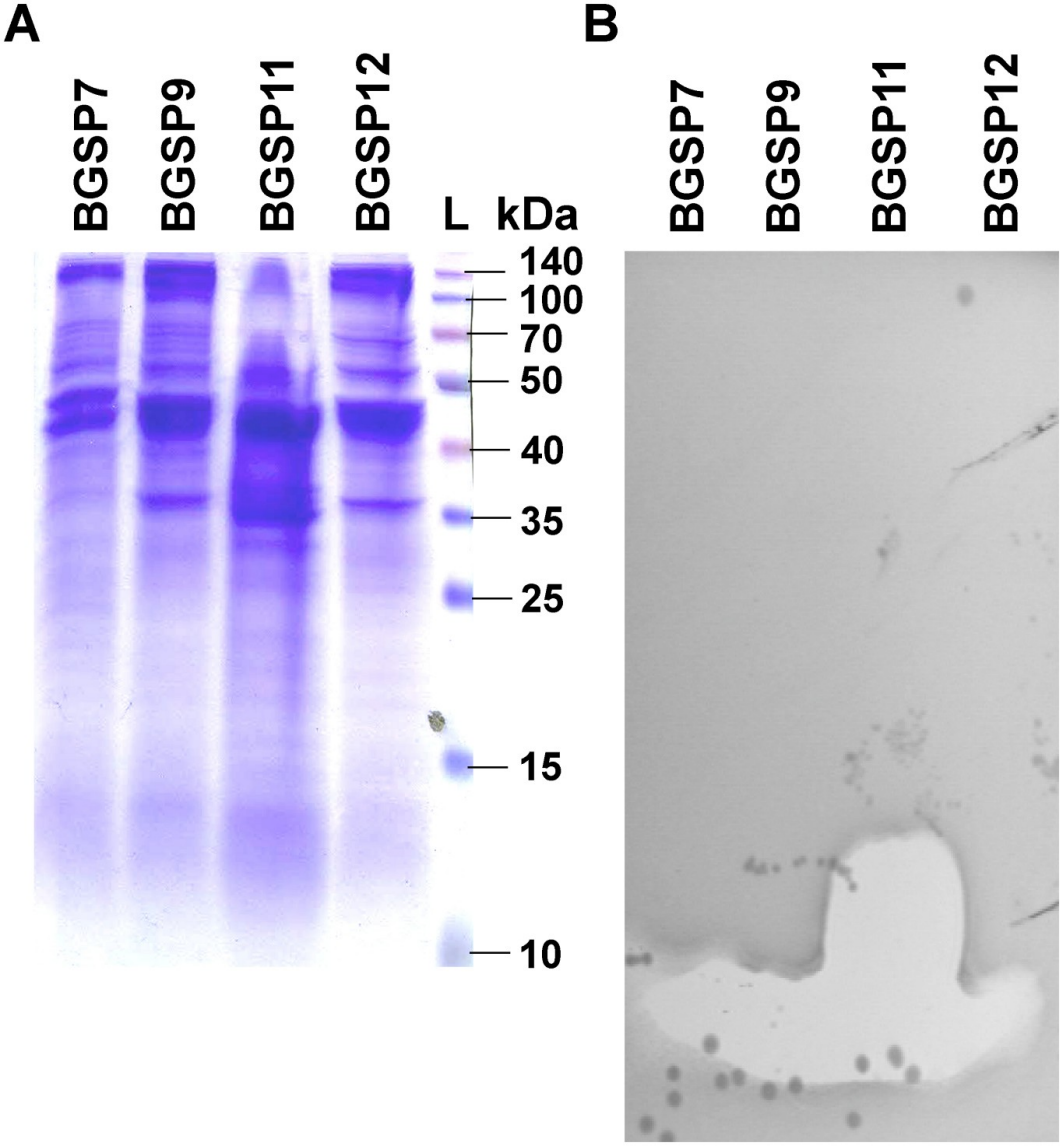
SDS-PAGE gel electrophoresis of proteins precipitated from supernatant of *Br*. *laterosporus* BGSP7, BGSP9, BGSP11 and BGSP12 strains on 15% acrylamide gel stained with Coomassie Brilliant Blue (A); gel with the same samples overlayed with LA-soft agar containing *S*. *aureus* ATCC25923 after incubation overnight at 37°C (B).

SDS PAGE analysis shows two clear zones of inhibition for strain BGSP11 and one zone for BGSP7 and BGSP9 (Fig 5). Zones of inhibition corresponding to small molecular mass molecule(s) (between 1–2 kDa) are evident for all three strains while, for strain BGSP11, an additional strong zone of inhibition corresponding to a mass of ~6 kDa was detected. The apparent production of at least one additional antimicrobial by BGSP11 explains why this strain produces larger zones of inhibition relative to BGSP7 and BGSP9 (Fig 1). In addition, according to extracellular protein profile of the strains (Fig 5) it can be concluded that isolates BGSP9 and BGSP12 are identical.

### Purification and molecular mass determination of antimicrobial molecules produced by *Br*. *laterosporus* BGSP7, BGSP9 and BGSP11

Antimicrobial molecules were purified from both cells and supernatants of *Br*. *laterosporus* BGSP7, BGSP9 and BGSP11 by Amberlite XAD (supernatant only), C18 solid phase extraction and Reversed Phase HPLC. A number of HPLC fractions from each strain were active and MALDI TOF mass spectrometry suggests that strains BGSP7, BGSP9 and BGSP11 produce a number of small antimicrobial molecules of different molecular masses. Three common molecular masses, i.e., 1557, 1570 and 1583 Da, are associated with the most active fractions produced by all three strains (S4–S6 Figs).

Specifically, antimicrobial peptide masses purified from BGSP7 were (i) from cells: 1583.73 Da—which correlates with the main peak on the chromatogram and a minor 1568.83 Da peptide (S4A Fig); (ii) from the supernatant: a 1583.94 Da mass again correlating with the most dominant peak and a minor 1570 Da (S4B Fig). The most active antimicrobial molecules purified from BGSP9 was (i) from cells: 1571.64 Da (fractions 32–33; these were the most active fractions and correlate with the biggest peak) and 1585.45 Da (S5A Fig); (ii) from supernatant: 1618.41 Da (fraction 24), 1555.79 and 1571.82 Da (fractions 25–27), 1557.19 Da (fraction 28), 1556.84, 1579.09 and 1603.16 (fractions 29–30), 1571.05 Da and 1603.22 (fractions 31–32) and 1583.91 Da (fractions 33–36); (S5B Fig). Antimicrobial molecules purified from BGSP11 were (i) from cells: 1556.54 Da (fraction 36), 1571.05Da (fractions 37–41), 1584.56 Da (fraction 42), 1223.97 and 1274.78Da (fractions 56–57) (S6A Fig); (ii) from supernatant: 1636.18 Da (fraction 19), 1618.95Da (fractions 29–30), 1564.90 Da (fractions 31–33), 1556.31 Da (fractions 34–37), 1570.11 Da (fractions 38–40), 1584.62 Da (fractions 41–44), 1585.44 and 1598.19 Da (fractions 45–51) and 1223.92 and 1274.02 Da (fraction 57) (S6B Fig). Based on the analysis of the molecular masses of active fractions, it can be concluded that the strains produce number of different antimicrobial molecules of molecular mass ranging from 1223.21 to 1657.13 Da, wherein the following polypeptide antibiotics can be classified: tyrocidine (from 1269.6 to 1361.7 Da), plipastatin (1463.7 Da) [67] and lipopeptides like brevibacillin (1583 Da), bogorols (A-1545, B-1570, C-1556, D-1602 and E-1618 Da) [27, 29] and Lh-1 of 1608.023 Da [68].

The most potent and abundant fractions, i.e., 1583.73 Da antimicrobial molecule from BGSP7 and 1556.31 Da from BGSP11, were selected for amino acid composition analysis. Amino acid analysis revealed that both peptides have a similar amino acid composition, but differ by virtue of the presence of in methionine, which is found only in the 1556.31 antimicrobial from the BGSP11 strain (Table 2).

**Table 2:** Amino acids analysis of antimicrobial peptides purified by RP-HPLC from BGSP7 and BGSP11 strains. Results are presented in percent (%).

Fractions from strains Amino acid	Molecular weight average (Da)	Peptide of 1583.73 Da from fractions 39–41 from cells wash of BGSP7 strain	Peptide of 1556.31 Da from fractions 34–37 from supernatant of BGSP11 strain
Threonine (Thr—T)	101.105	-	0.0757
Serine (Ser—S)	87.078	0.1685	0.3528
Glutamic acid (Glu—E)	129.116	0.2693	0.8266
Proline (Pro—P)	97.117	0.5710	1.2604
Glycine (Gly—G)	57.052	0.0328	0.1684
Alanine (Ala—A)	71.019	0.0606	0.1885
Cysteine (Cys—C)	103.144	0.2046	0.5951
Valine (Val—V)	99.133	10.5905	10.9690
Methionine (Met—M)	131.198	0.1754	2.3586
Isoleucine (Ile—I)	113.160	6.0451	2.5983
Leucine (Leu—L)	113.160	11.9155	8.5396
Tyrosine (Tyr—Y)	163.170	9.1807	7.9586
Phenylalanine (Phe—F)	147.177	0.7485	1.1338
Histidine (His—H)	137.142	0.0614	-
Lysine (Lys—K)	128.174	12.7185	8.8001
Arginine (Arg—R)	156.188	0.0816	0.3034
Total amino acids		52.7422	45.8254

### Minimal inhibitory concentrations (MICs) of purified antimicrobial molecules required to inhibit selected indicators

The minimal inhibitory concentrations (MICs) of the two most abundant antimicrobial molecules, 1583.73 Da from BGSP7 and 1556.31 Da from BGSP11, against a number of indicator strains were determined. Both antimicrobial molecules showed strong antimicrobial activity against all tested Gram-positive and Gram-negative strains, although small differences were evident with respect to *Achromobacter xylosoxidans* MS4 (two times higher for antimicrobial molecule of 1556.31 Da), *Pseudomonas syringae* (eight times higher for antimicrobial molecule 1583.73 Da) and *Salmonella enteritidis* ATCC13706 (two times higher for antimicrobial molecule of 1556.31 Da) (Table 3).

**Table 3:** Minimal inhibitory concentrations (MICs).

Strains	MIC (μΜ)	
	Antimicrobial molecule 1583.73 Da	Antimicrobial molecule 1556.31 Da
*Achromobacter xylosoxidans* MS4	158	316
*Acinetobacter baumannii* 6077/12	20	20
*Agrobacterium tumefaciens*	20	20
*Bacillus cereus* ATCC11778	5	5
*Bacillus subtilis* 168	5	5
*Burkholderia cepacia*	5	5
*Burkholderia glumae*	5	5
*Chromobacterium violaceum*	316	316
*Enterococcus faecalis* ZLS10-27	10	10
*Erwinia carotovora*	79	79
*Escherichia coli* ATCC25922	79	79
*Klebsiella pneumoniae* Ni9	316	316
*Lactobacillus paracasei* BGSJ2-8	5	5
*Lactobacillus plantarum* 9208	5	5
*Lactobacillus zeae*	5	5
*Lactococcus cremoris* MG7284	5	5
*Lactococcus lactis* BGBU1-4	5	5
*Lactococcus raffinolactis* BGTRK10-1	5	5
*Listeria monocytogenes* ATCC19111	5	5
*Paenibacillus larvae*	5	5
*Pseudomonas aeruginosa* MMA83	158	158
*Pseudomonas syringae*	158	20
*Ralstonia pickettii* 666	316	316
*Salmonella enteritidis* ATCC13706	158	31 6
*Staphylococcus aureus* ATCC25923	5	5
*Streptococcus agalactiae* B165	10	10
*Streptococcus mutans* BGSF1	5	5
*Streptococcus pneumoniae* P173	5	5
*Streptococcus pyogenes* A2941	5	5
*Streptococcus thermophilus*	5	5
*Xanthomonas oryzae*	316	316

### Comparative analysis of *Br*. *laterosporus* BGSP7, BGSP9 and BGSP11 genome sequences

The genomes of *Br*. *laterosporus* BGSP7, BGSP9 and BGSP11 were sequenced. The genomes (5,335,373 bp—BGSP7; 5,801,339 bp—BGSP9 and 5,643,326 bp—BGSP11) consisted of one chromosome and one circular plasmid of 9,427 bp (pBLSP7 of BGSP7), 9,158 bp (pBLSP9 of BGSP9) or 8,578 bp (pBLSP11 of BGSP11), with a total GC content of about 40% (Table 4). Plasmids pBLSP9 and pBLSP11 showed a high degree of identity (93%), but only 27% with plasmid pBLSP7. BLAST analysis found that pBLSP7 shares 88% of identity (on 60% coverage) with plasmid pBRLA07 from *Br*. *laterosporus* LMG 15441, while plasmids pBLSP9 and pBLSP11 showed 85% identity (on 40% coverage) with pBRLA07.

**Table 4:** Summary statistics for genome sequence assemblies.

Strain	Assembly accession number	Number of contigs	Total length (bp)	Number of genes	Contig N50 (bp)	G+C content (%)
BGSP7	GCA_002927075.1	111	5,335,373	4962	185,683	40.10
BGSP9	GCA_002927085.1	193	5,801,339	5313	117,738	40.24
BGSP11	GCA_002926995.1	157	5,643,326	5291	128,934	40.00

Comparative analysis of genomes shows that there are differences in both genome size and consequently in the numbers of predicted genes. Strain BGSP7 possesses 197 genes that are not present in BGSP9 and BGSP11 strains (S2A Table); BGSP9 strain possesses 228 genes that are not present in BGSP7 and BGSP11 strains (S2B Table) and strain BGSP11 possesses 256 genes that are not present in BGSP7 and BGSP9 strains (S2C Table). These differences may be responsible for strain specific phenotypic characteristics. Most of these differentially present/absent genes are mobile elements, type I restriction-modification systems, phages and hypothetical proteins. Among the genes that might plausibly contribute to the phenotypic differences are, in strain BGSP7, a stage V sporulation protein, arsenate reductase and mosquitocidal toxin; in strain BGSP9, a lanthionine biosynthesis protein LanB, lanthionine biosynthesis cyclase LanC and flagellar protein FlgJ; and in strain BGSP11, an oxetanocin A resistance protein, ferric siderophore transport system and a spore coat protein A.

### *Br*. *laterosporus* BGSP7, BGSP9 and BGSP11 contain operons/gene clusters predicted to encode the production of bacteriocins and secondary metabolites

The genomes of all three strains were searched for the presence of secondary-metabolism biosynthesis genes using AntiSMASH (Antibiotics & Secondary Metabolite Analysis Shell) [69] and BAGEL3 (identification of genes encoding bacteriocins and non-bactericidal post-translationally modified peptides) [70]. All three strains possess a number of genes that could possibly encode known antimicrobial molecules. Results of AntiSMASH and BAGEL3 searches are presented in Table 5 for all three strains. In addition, BLAST searches revealed that all three strains possess candidate genes for production of the bacteriocin laterosporulin. The predicted products of these genes showed 57% amino-acid identity (strain BGSP7) and 63% identity (strains BGSP9 and BGSP11) with known laterosporulim proteins from *Br*. *laterosporus* GI-9 and LMG15441 (GenBank accession numbers: CCD21955.1 and AIG26526.1, respectively). A search for genes encoding ribosomally synthesized antimicrobial peptides revealed that all three strains had the potential to produce numerous bacteriocins in addition to laterosporulin, lactococcin 972-like bacteriocin, linocin M18, LAPs, a sactipeptide, UviB and in addition strain BGSP9 possesses genes for the synthesis of a lantipeptide (Table 5). The semi-native SDS PAGE protein overlay antimicrobial assay suggests that only strain BGSP11 is able to produce an antimicrobial molecule of about 6 kDa that could be bacteriocin laterosporulin.

**Table 5:** Genome-based identification of novel molecules with antimicrobial activity by bioinformatic tools (AntiSMASH and BAGEL3).

Antimicrobial molecule	Strain	Detected by	Amino Acid Sequence	Identity (%) among strains BGSP7, BGSP9 and BGSP11
Laterosporulin	BGSP7	**BLAST**	MACANQCPDAVKSWAYTDYQCHPVNKKWYRQVYAVCMNGANLYCKTEWSTKC	BGSP7:BGSP9 = 86 BGSP7:BGSP11 = 86 BGSP9:BGSP11 = 100
	BGSP9	**BLAST**	MACVNQCPDAVKSWSYTDYQCHPVERKYYRHVYAVCMNGLNLYCKTEWSTKC	
	BGSP11	**BLAST**	MACVNQCPDAVKSWSYTDYQCHPVERKYYRHVYAVCMNGLNLYCKTEWSTKC	
Linocin M18	BGSP7	**AntiSMASH**	MDKSQKFPDSPLSKEEWRQLDETIVEMARRQLVGRRFIDIYGPLGEGIQTITNDIYDESRFGNMSLRGESLELTQPSKRVSLTIPIVYKDFMLYWRDMAQARTLGMPIDLSPAANAASSCALMEDDLIFNGNPEFDLPGIMNVKGRLTHIKSDWMESGNAFADIVEARNKLLKMGHSGPYALVVSPELYSLLHRVHKGTNVLEIDHIRNLVTDGVFQSPVIKGGALVATGRHNLDLAIAEDFDSAFLGDEQMNSLMRVYECAVLRIKRPSAICTLETTEE	BGSP7:BGSP9 = 100 BGSP7:BGSP11 = 99 BGSP9:BGSP11 = 99
	BGSP9	**AntiSMASH**	MDKSQKFPDSPLSKEEWRQLDETIVEMARRQLVGRRFIDIYGPLGEGIQTITNDIYDESRFGNMSLRGESLELTQPSKRVSLTIPIVYKDFMLYWRDMAQARTLGMPIDLSPAANAASSCALMEDDLIFNGNPEFDLPGIMNVKGRLTHIKSDWMESGNAFADIVEARNKLLKMGHSGPYALVVSPELYSLLHRVHKGTNVLEIDHIRNLVTDGVFQSPVIKGGALVATGRHNLDLAIAEDFDSAFLGDEQMNSLMRVYECAVLRIKRPSAICTLETTEE	
	BGSP11	**AntiSMASH**	MDKSQKFPDSPLSKEEWRQLDETIVEMARRQLVGRRFIDIYGPLGEGIQTITNDIYDESRFGNMSLRGESLELTQPSKRVSLTIPIVYKDFMLYWRDMAQARTLGMPIDLSPAANAASSCALMEDDLIFNGNPEFDLPGIMNVKGRLTHIKSDWMESGNAFADIVEARNKLLKMGHSGPYALVVSPELYSLLHRVHKGTNVLEIDHIRNLVTDGVFQSPVIKGGAVATGRHNLDLAIAEDFDSAFLGDEQMNSLMRVYECAVLRIKRPSAICTLETIEE	
Lactococcin 972-like	BGSP7	**AntiSMASH**	MMLKKSMVGLIAVLTLAMGSQAMATDGKAELVNSNVTPFKTIGAGGGDWNYLIQIWCSKEMLVPLQTPGQFSFCDSYHRRR	BGSP7:BGSP9 = 45 BGSP7:BGSP11 = 44 BGSP9:BGSP11 = 89
	BGSP9	**AntiSMASH**	MMLKKSMVGLIAVLTLAMGSQAMATDGKAELVNSNVTPFKTIGIGGGDWNYGSSIIGAQKKCWSHYKHPDNFHSATAIIGDDEKTDYAEAGEWAKADAYADKKNTCYTHWDDEPKRPK	
	BGSP11	**AntiSMASH**	MMLKKSMVGLIAVLTLAMGSQAMATDGKAELVNSNVTPFKTTGIGGGDWNYGSSKIGAQKKCWSYYKHPEKFHSATAIIGDDEDTDYAEAGEWAKAEAYADKKYTCYAHWDNKAKRPK	
Linear Azole containing peptides (LAPs)	BGSP7	**BAGEL3**	MDDFQNELKKLRVDKFQGGDVSPWENESQQDAMLVQRRCGRCHHCSGSCSCSCSCSCSCSCSCSCVCLFINCFRCSRCSRC	BGSP7:BGSP9 = 87 BGSP7:BGSP11 = 87 BGSP9:BGSP11 = 100
	BGSP9	**BAGEL3**	MDDFQNELKKLRVDKFKGGDVSPWENESQQDAMLVQRRGGRCQRCSCSCSCSCSCSCSCSCSCSCSCSCFCIFINCFRCSRCSRCF	
	BGSP11	**BAGEL3**	MDDFQNELKKLRVDKFKGGDVSPWENESQQDAMLVQRRGGRCQRCSCSCSCSCSCSCSCSCSCSCSCSCFCIFINCFRCSRCSRCF	
Sactipeptide	BGSP7	**BAGEL3**	MKNYTTPKVKVVNPGVIDVIDSCQCGSKNGAGA	BGSP7:BGSP9 = 88 BGSP7:BGSP11 = 88 BGSP9:BGSP11 = 100
	BGSP9	**BAGEL3**	MKNYTTPKVKVVNPGVTDVVDSCQCGAKNGAGA	
	BGSP11	**BAGEL3**	MKNYTTPKVKVVNPGVTDVVDSCQCGAKNGAGA	
UviB	BGSP7	**BAGEL3**	MEESVMNALLQQGPFAALFVWLLFSTKKEGRDRETRLVKQAQAREAKLMEHNERMVIQLERNTSTLQQIERSLSGLEMELQELKEKVE	BGSP7:BGSP9 = 73 BGSP7:BGSP11 = 88 BGSP9:BGSP11 = 99
	BGSP9	**BAGEL3**	MEESVMNALLQQGPFAALFVWLLFSTKKEGRDRETRLVKQAQAREAKLMEHNERMVIQLE	
	BGSP11	**BAGEL3**	MEESVLNALLQQGPFAALFVWLLFSTKKEGRDRETRLVKQAQAREAKLMEHNERMVIQLERNTSTLQQIERSLSGLEMELQELKEKVE	
Lantipeptide	BGSP9	**BAGEL3**	MKKEDLFDLDVQVKEASQAQGDSVVSDLICTTFCSATFCQSNCC	

Since all three strains produce non-ribosomally synthesized antimicrobial molecules of molecular mass between 1.2 and 1.6 kDa (Fig 5), the genomes of all three strains were analyzed for the presence of synthase genes. It was found that all three strains possess numerous synthase genes grouped/distributed into a number of gene clusters at different locations on the genomes: the genomes of BGSP7, BGSP9 and BGSP11 strains possess 56, 70 and 56 synthase genes, respectively (Table 6).

**Table 6:** Synthase genes present in genomes of *Br*. *laterosporus* BGSP7, BGSP9 and BGSP11 strains.

Synthase gene product	Number of genes/products in chromosome of strains		
	BGSP7	BGSP9	BGSP11
Gramicidin S synthase 1 (GrsA)	2	2	2
Gramicidin S synthase 2 (GrsB)	12	12	12
Linear gramicidin synthase subunit B (LgrB)	2	3	2
Linear gramicidin synthase subunit D (LgrD)	3	2	2
Polyketide synthase (PksB)	1	2	1
Polyketide synthase (PksE)	1	2	1
Polyketide synthase (PksF)	1	2	1
Polyketide synthase (PksG)	1	2	1
Polyketide synthase (PksH)	1	2	1
Polyketide synthase (PksI)		1	
Polyketide synthase (PksJ)	2	3	2
Polyketide synthase (PksL)	1	3	1
Polyketide synthase (PksM)	2	5	2
Polyketide synthase (PksN)	5	7	5
Polyketide synthase (PksR)	1	2	1
Polyketide biosynthesis acyl-carrier-protein (AcpK)	1	1	2
Phthiocerol/phenolphthiocerol synthesis polyketide synthase type I (PpsC)	2		
Phthiocerol/phenolphthiocerol synthesis polyketide synthase type I (PpsE)	1		
Polyketide biosynthesis protein (BaeE)	1	1	1
Plipastatin synthase subunit B (PpsB)	1	1	1
Plipastatin synthase subunit C (PpsC)	1	1	1
Plipastatin synthase subunit D (PpsD)	1	1	1
Plipastatin synthase subunit E (PpsE)		1	1
Surfactin synthase subunit 1 (SrfAA)	4	3	4
Tyrocidine synthase 3 (TycC)	8	10	10
EryA	1	1	1
**Total number of genes / products**	**56**	**70**	**56**

**Fig 6:**
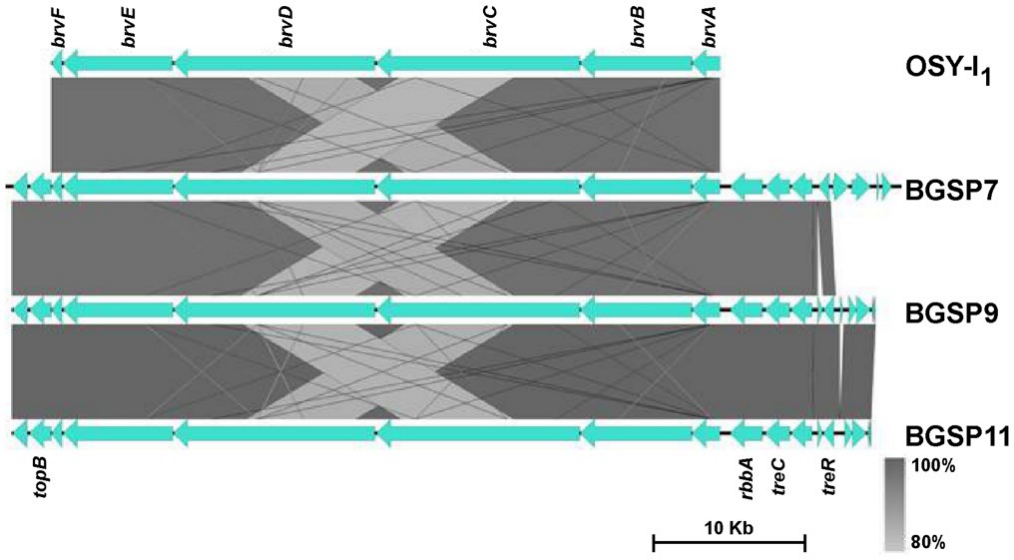
Comparative analysis of brevibacillin gene cluster among *Brevibacillus laterosporus* strains. *brvA*—brevibacillin synthetase A, *brvB*—brevibacillin synthetase B, *brvC*—brevibacillin synthetase C, *brvD*—brevibacillin synthetase D, *brvE*—brevibacillin synthetase E, and *brvF*—brevibacillin ABC-transporter F genes.

Brevibacillin is a potent 1583 Da antimicrobial lipopeptide produced by *Br*. *laterosporus* OSY-I_1_ [27]. The brevibacillin gene cluster consists of five brevibacillin synthase genes (*brvA*, *brvB*, *brvC*, *brvD*, *brvE*) and one ABC transporter gene (*brvF*) (Accession No. MF526970.1; [38]. Since the most abundant antimicrobial molecule produced by strains BGSP7, BGSP9 and BGSP11 was 1583 Da, the same mass as brevibacillin, genomes were searched for brevibacillin synthesis gene cluster. The brevibacillin gene cluster was found in each of the three strains positioned between the *rbbA* gene for ribosome-associated ATPase and the *topB* gene for DNA topoisomerase 3. Amino-acid identity between the synthase proteins is very high among BGSP strains and also with those from strain OSY-I_1_ (between 96–99%) with the highest identity between BGSP7 strain and OSY-I_1_ (Fig 6). It is interesting to note that the strain BGSP9 possess an unusually large gene cluster of 99.8 kb on the chromosome, consisting of 16 genes for polyketide synthases (PksB, PksE, PksM1, PksN1, PksL1, PksM2, PksL2, PksN2, AcpK, PksF, PksG, PksH, PksI, PksM3, PksJ and PksR) unlike the other strains.

### Bacteriocin gene expression analysis by RT-qPCR

**Fig 7:**
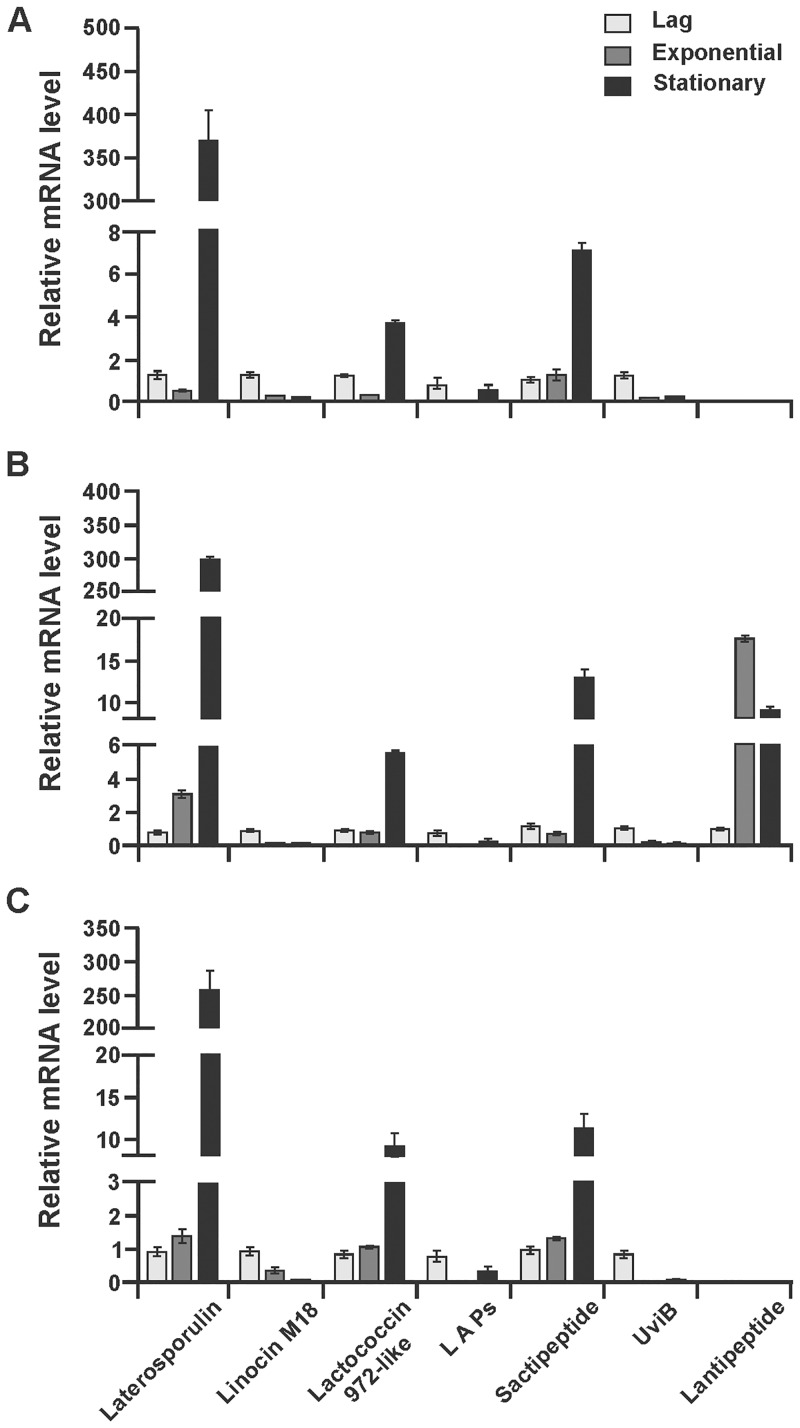
Bacteriocin gene transcription analysis of *Br*. *laterosporus* BGSP7 (A); BGSP9 (B) and BGSP11 (C) by RT-qPCR.

Genome analysis has shown that all three strains have numerous genes encoding for bacteriocin production, and the semi-native protein gel result (Fig 5) in combination with HPLC analysis has shown that all three strains synthesize small peptides. The SDS gel also suggests that strain BGSP11 produces a 6 kDa bacteriocin which is most probably latersporulin. Comparative analysis of gene clusters revealed that there are some differences in the structural genes for bacteriocin-like substances (Table 5). To elucidate if the structural genes are functional (transcribed) the RT-qPCR method was performed using primers specific for each bacteriocin structural gene (S1C Table). The relative expression level of each gene was calculated for each growth phase (lag, exponential and stationary) in order to determine the level of bacteriocin gene transcription during growth in LB medium. The RT-qPCR analysis of total RNA samples from different growth phases show that the gene encoding the laterosporulin structural peptide presented the highest level of transcription in 16h old culture for all three strains (more than 200-fold increase). In strains BGSP9 and BGSP11 there is direct correlation between growth and laterosporulin gene transcription, while in strain BGSP7 there is a decrease in gene transcription during exponential phase (Fig 7). Also, transcription of the genes for the synthesis of sactipeptides and lactococcin 972-like bacteriocin was significantly increased during the stationary phase in all three strains, with differences as transcription is gradually increased in correlation with cell density for sactipeptides in strains BGSP7 and BGSP11 and for the lacococcin 972-like bacteriocin in BGSP11 strain, while transcription of genes for sactipeptides and lacococcin 972-like bacteriocin in strain BGSP9 have reduced in exponential growth phase and in strain BGSP7 transcription of gene for lacococcin 972-like bacteriocin is almost completely absent in exponential growth phase. Transcription of the gene for the bacteriocin linocin M18 has a negative correlation with the increase in cell density in all three strains. Similar results for gene transcription were obtained for LAPs and UviB in all three strains. Transcription of the lantipeptide gene in strain BGSP9 showed very high levels with the pattern of increase in exponential and reduction in stationary phase of growth (Fig 7B). RTqPCR analysis revealed that all selected bacteriocin genes present in *Br*. *laterosporus* BGSP7, BGSP9 and BGSP11 strains are regulated on a transcription level in a growth phase-dependent manner.

### Selection of clones carrying bacteriocin operons and expression in heterologous hosts

Cosmid libraries of *Br*. *laterosporus* BGSP7, BGSP9 and BGSP11 strains were constructed and analysis revealed that laterosporulin and lactococcin 972-like bacteriocin clusters seem to be complete because they contained a structural gene, a hypothetical protein-most likely responsible for immunity, two transporters and a transcription regulator and differ among the strains. Cosmid clones, carrying bacteriocin genes clusters were cloned into vectors replicating in Gram-positive bacteria (pAZIL for *L*. *lactis* and pA13 for *B*. *subtilis*) giving corresponding constructs: pAZIL-ELS7, pAZIL-ELS9, pAZIL-ELS11, pAZIL-ELC7, pAZIL-ELC9 and pAZIL-ELC11, pA13-ELS7, pA13-ELS9, pA13-ELS11, pA13-ELC7, pA13-ELC9 and pA13-ELC11. *B*. *subtilis* 168 and *L*. *lactis* subsp. *cremoris* MG7284 were chosen for heterologous expression of laterosporulin and lactococcin 972-like bacteriocin gene clusters. *B*. *subtilis* 168 transformants were checked for enhanced antimicrobial activity on *S*. *aureus* ATCC25923 (since strain *B*. *subtilis* 168 produces a cyclic peptide antibiotic, subtilosin A) and confirmed for plasmid integrity after re-transformation of DH5α and by restriction enzyme analysis. Antimicrobial activity of *L*. *lactis* subsp. *cremoris* MG7284 transformants against *L*. *lactis* subsp. *cremoris* MG7284, *S*. *aureus* ATCC25923 and *L*. *monocytogenes* ATCC19111 was tested. None of the transformants obtained in strains *B*. *subtilis* 168 and *L*. *lactis* subsp. *cremoris* MG7284 produced antimicrobial molecules indicating that cloned gene clusters are not expressed in the heterologous hosts used for expression.

## Discussion

The emergence of resistance to antibiotics, has created an urgent need for new antimicrobials and has focused research in two directions i) towards the direct isolation of new bacterial isolates with antimicrobial activity and ii) the analysis of metagenomes for the presence of genes/operons with potential for novel antimicrobial production [71]. In this study, clover silage samples were assayed for antipathogenic potential and three new *Br*. *laterosporus* strains (BGSP7, BGSP9 and BGSP11) were isolated showing strong antimicrobial activity against Gram-positive and Gram-negative food-borne spoilage and pathogenic bacteria from humans, animals and plants. Members of the species *Br*. *laterosporus* do not cause disease or harmful effects on humans, animals and plants and are even considered/used as probiotics [72]. The bioactive compounds produced by *Br*. *laterosporus* SA14 include antimicrobial peptides which are medically important substances that may be used for alternative treatment of MRSA infection [73]. This genus continues to be a source of numerous enzymes of great biotechnological interest due to their ability to biodegrade low density polyethylene and ability to act as a candidate biocontrol agent [22].

Silage is a common fermented ruminant food prepared from fresh grass or corn stalk. The most frequent bacterial species that contribute to the quality and durability (shelf life) of silage are lactobacilli and *Brevibacillus*, which most likely enter from the environment [74]. Antimicrobial molecules with narrow or broad spectrum activity are often produced by bacterial strains to compete with other microbes, which inhabit the same ecological niches including different strains of the same species. In recent years, research on *Br*. *laterosporus* strains has intensified, as it is a non-pathogenic bacterium with the ability to synthesize a significant number of antimicrobial molecules. Recently, different antimicrobial peptides [18, 30, 32], lipopeptides [27, 67, 75] and cyclic dipeptides [76] have been isolated and characterized from strains of *Br*. *laterosporus* with broad-spectrum antimicrobial activity. In this study we investigate the antibacterial potential of three new isolated *Br*. *laterosporus* BGSP7, BGSP9 and BGSP11 strains from clover silage. All three strains show inhibitory activity towards a number of pathogenic bacteria, without the emergence of resistant colonies even after several days of incubation. It is interesting that In addition, it has been observed that strains show cross inhibition, even auto-inhibition was noticed (data not shown), which is most probably due to the synthesis of a large number of diverse antimicrobial molecules. It is interesting that the period of the inhibition of Gram-positive and Gram-negative bacteria by synthesized antimicrobial compounds of isolated strains BGSP7, BGSP9 and BGSP11 is different (Fig 4) suggesting two possibilities: that the synthesized molecule(s) have a different antimicrobial effect on various bacteria as shown in the analysis of the MIC values for two of them (Table 3), or at different time period are synthesized antimicrobial molecules of different specificities.

Crude cell free antimicrobial extracts (50% saturation of ammonium sulfate) from BGSP7, BGSP9 and BGSP11 are stable across a wide temperature and pH range and during storage for up to 1 year at +4°C. These are desirable properties that can increase the potential applications of the strains. It has been shown that laterosporulin, produced by *Brevibacillus* sp. strain GI-9 is thermostable, pH tolerant and resistant to proteolytic enzymes [30]. Purified brevibacillin showed no sign of degradation when it was held at 80°C for 60 min, and it retained at least 50% of its antimicrobial activity when it was held for 22h under acidic or alkaline conditions [27].

It is interesting that the most abundant antimicrobial molecule of 1583 Da, purified from BGSP7 shows low MIC values against Gram-negative pathogens unlike brevibacillin, which is a lipopeptide of the same molecular mass [27] indicating differences in the molecule produced by BGSP7. Two of the most potent and abundant fractions (one from BGSP7-1583.73 Da and second from BGSP11-1556.31 Da) were analyzed by N-terminal amino acid sequencing that failed because N-terminus was protected most likely due to the presence of a non-proteinaceous component. Total amino acid analysis was then performed and results revealed that the amino acid content of the samples was 52.7422 and 45.8254% respectively suggesting the presence of non-proteinaceous components (Table 2). It is interesting that almost all antimicrobial peptides and lipopeptides contain similar amino acids residues, specifically the aliphatic aminio acids: Leu, Ile and Val, cationic: Lys and polar, aromatic: Tyr. Examples include gramicidin (Val-Gly-Ala-Leu-Ala-Val-Val-Val-Trp-Leu-Tyr-Leu-Trp-Leu-Trp), tyrocidine cyclic decapeptide (DPhe-Pro-Phe-DPhe-Asn-Gln-Tyr-Val-Orn-Leu), plipastatin cyclodecapeptide (Glu-Orn-Tyr-aThr-Glu-Ala/Val-Pro-Glu-Tyr-Ile), brevibacillin (FA-Dhb-Leu-Orn-Ile-Ile-Val-Lys-Val-Val-Lys-Tyr-Leu-Valinol), bogorols (Hmp-Aba-Leu-Orn-Ile-Val-Val-Lys-Val-Leu-Lys-Tyr-Leu-Valinol) laterocidin (Tyr-Pro-Phe-Phe-Asn-Asp-Leu-Val-Orn-Leu), surfactin (Glu-Leu-Leu-Val-Asp-Leu-Leu/Ile) and iturin (Asn-Tyr-Asn-Glu-Asn-Ser) [21, 29]. It is not possible to determine the sequence of the antimicrobial molecules from the amino acid analysis results of these two fractions, but the presence and ratio of amino acids indicates that the strains produce similar, but not identical antimicrobial molecules to those previously described. This is particularly evident for the 1556.31 Da antimicrobial molecule produced by strain BGSP11 as it contains the amino acid methionine which presence has been detected only in bogorols D and E, both of which of different molecular mass (1602 and 1618 Da, respectively) [29]. BGSP strains isolated from silage produce novel mutually similar complex antimicrobial molecules that differ slightly in amino acid composition. It is demonstrated that changes in amino acid composition of LsbB bacteriocin can drastically affect activity [77]. In addition to the differences in the protein portion, the antimicrobial molecules could also differ in the non-protein part, so that a greater variety of molecules can be expected among isolated strains. Because of the similar molecular mass and amino acid composition, we assume that most of the antimicrobial molecules produced by BGSP strains are lipoproteins similar to brevibacillin [27] or bogorol variants [29]. Determining the sequence of different antimicrobial molecules purified from BGSP strains by LC MS/MS and structure using nuclear magnetic resonance (NMR) will be the subject of future research.

Powerful molecular mining approaches using bioinformatics tools (e.g., AntiSMASH and BAGEL3) are available for identification of genetic determinants of antimicrobial production. [78]. Zhao and Kuipers [18] provided a classification scheme of known and putative antimicrobial compounds produced by a wide variety of *Bacillales* species using web based genome-mining prediction tools. In order to fully study the antimicrobial potential of isolated strains, we applied two approaches: a) complete genome sequencing applying both AntiSMASH and BAGEL3 searches for the presence of genes (operons, gene clusters) encoding antimicrobials and b) purification of antimicrobials from culture supernatant and the cell surface. The genome search showed that all three strains had the potential to produce numerous antimicrobials including bacteriocins, non-ribosomally synthetized polypeptides and lipopeptides. A purification approach confirmed that BGSP strains produce a large number of small molecular mass antimicrobials (between 1200–1600 Da) and strain BGSP11 seems to produces one bacteriocin of about 6 kDa, which is most likely laterosporulin that shows only 57% identity with GI-9 strain. It is possible that the 6 kDa zone of inhibition produced by strain BGSP11 on the SDS gel could be due to multimerization of lipopeptide antimicrobial molecules, as noted by the Alajlani and coauthors [79] in *Bacillus subtilis* strain BIA, since N terminal sequencing of the protein band failed. The transcriptional analysis of the genes for the synthesis of bacteriocins showed that they were all transcribed, but that the transcription was regulated in a growth phase-dependent manner. In order to overcome this problem, we cloned gene clusters for two bacteriocins (laterosporulin and lactococcin 972-like bacteriocin) and tried to express them in the heterologous host, but it was unsuccessful. Singh and coauthors [30] also tried to express a 4 kb fragment carrying laterosporulin gene cluster from *Brevibacillus* sp. strain GI-9, also unsuccessfully, indicating that most probably there are other host factors involved in the expression. The next approach that we plan to apply will be mutagenesis or phage induction, that may trigger bacteriocin synthesis as demonstrated by Brady and coauthors [80]. Among *Brevibacillus* species genetic manipulations are well developed for *Br*. *choshinensis* strains and it is used as Gram positive expression system (Takara *Brevibacillus* expression system). According to our best knowledge tools for genetic manipulations in *Br*. *laterosporus* are still lacking, but since many strains possess plasmids that can be used for the construction of tools/vectors for cloning, expression and mutagenesis and because of the importance of these bacteria there is a need for its development. Plasmids from BGSP strains showed homology (at different levels) with only one plasmid characterized in strains of *Br*. *laterosporus* (pBRLA07) indicating their different origins. An additional characteristic of *Br*. *laterosporus* strains, which makes them suitable for genetic manipulation, is an exceptional antibiotic sensitivity (less than or about 1 μg/ml for erythromycin, chloramphenicol and tetracycline).

In addition to antibacterial activity, strains of *Brevibacillus* are well known producers of antifungal agents [22] and they are also active against insects and nematodes [25, 26]. *Br*. *laterosporus* is an invertebrate pathogen that is characterized by a unique spore coat and canoe-shaped parasporal body (SC-CSPB) complex surrounding the core spore. Some of the proteins associated with the spore coat, exosporium and CSPB complex represent putative virulence factors acting against insects. In addition, these proteins are progressively synthetized during bacterial growth, a proportional increase in the insecticidal activity of this bacterium was observed, being fully toxic when the spore envelopes are completely formed [81]. Preliminary results obtained on potato beetle (*Leptinotarsa decemlineata*) indicate that BGSP7, BGSP9 and BGSP11 strains show a high degree of antagonism, both against larval forms and adults, although BGSP11 strain shows the highest efficacy, most probably because it possesses an additional spore coat protein A (unpublished data). It is well known that some strains of *Br*. *laterosporus* are highly toxic for mosquitoes [82] because they produce crystalline inclusions of various shapes and sizes [83]. The genome search revealed that strain BGSP7 possesses a gene for mosquitocidal toxin that could be involved in anti-insecticidal activity. Preliminary tests performed on a limited number of pathogenic fungi have shown that strains BGSP7, BGSP9 and BGSP11 have the potential to inhibit the growth of several pathogenic fungi (unpublished data).

Based on the results obtained in this study it can be concluded that novel strains of *Br*. *laterosporus* BGSP7, BGSP9 and BGSP11 produce a number of antimicrobial molecules that are active against various Gram-positive and Gram-negative pathogens of humans, animals and plants with MIC values lower than nisin for many strains used in analysis and represent good candidates for isolation and application of various novel antimicrobial molecules (bacteriocins, antimicrobial peptides, lipopeptides and polyketides), as well as for biological control.

## Supporting information

S1 Fig Surface colonization by *Br*. *laterosporus* BGSP7, BGSP9 and BGSP11 strains on defined semi-solid media is dependent on the level of potassium ion and agarose concentration. Semi-solid MSggN agarose plates (0.3 to 1.5% w/v agarose and 100 μM or 5 mM KCl) were inoculated in the center with 2μl of culture of the strains (in triplicate). After growth for 24 h at 37°C, typical plates were photographed.
(TIF)

S2 Fig Gel electrophoresis of plasmid DNA isolated from *Br*. *laterosporus* BGSP7, BGSP9, BGSP11 and BGSP12 on 1% agarose gel containing ethidium bromide (200 μg/ml) photographed under UV light. (TIF)

S3 Fig The agar well diffusion assay. Antimicrobial activity of *Br*. *laterosporus* BGSP7 (3), BGSP9 (4) and BGSP11 (5) strains on *P*. *larvae* PC19726256. Negative control *P*. *larvae* PC19726256 (1) and nisin producer *L*. *lactis* (2) as positive control, were used.
(TIF)

S4 Fig Reverse-phase high-performance liquid chromatography (RP-HPLC) chromatogram from the cell wash (A) and supernatant (B) from BGSP7 strain. Matrix-assisted laser desorption ionization–time of flight (MALDI-TOF) mass spectrometry data from the fractions 33–36; 1568.83 Da (Aa), fractions 37–41; 1583.73 Da (Ab), fractions 29–42; 1583.94 Da (Ba), fractions 56–57; 1569.87 (Bb).
(TIF)

S5 Fig Reverse-phase high-performance liquid chromatography (RP-HPLC) chromatogram from the cell wash (A) and supernatant (B) from BGSP9 strain. Matrix-assisted laser desorption ionization–time of flight (MALDI-TOF) mass spectrometry data from the fractions 24–25; 1657.53 Da (Aa), fraction 28; 1611.46 Da (Ab), fraction 29; 1557.37 Da (Ac), fraction 30; 1625.44 and 1603.16 Da (Ad), fractions 32–33; 1571.64 Da (Ae), fractions 34–36; 1585.45 Da (Af), fraction 24; 1618.41 Da (Ba), fractions 25–27; 1571.82 and 1555.79 Da (Bb), fraction 28; 1557.19 Da (Bc), fractions 29–30; 1579.09,1556.84 (Bd) and 1603.16 Da (Be), fractions 31–32; 1571.05 and 1603.22 Da (Bf), fractions 33–36; 1583.91 Da (Bg) and fraction 37; 1584.86 Da (Bh).
(TIF)

S6 Fig Reverse-phase high-performance liquid chromatography (RP-HPLC) chromatogram from the cell wash (A) and supernatant (B) from BGSP11 strain. Matrix-assisted laser desorption ionization–time of flight (MALDI-TOF) mass spectrometry data from the fraction 36; 1556.54 Da (Aa), fractions 37–41; 1571.05 Da (Ab), fractions 42–44; 1584.56 Da (Ac), fractions 56–57; 1223.97 (Ad) and 1274.78 Da (Ae), fraction 19; 1636.18 Da (Ba), fraction 29–30; 1618.95 Da (Bb), fractions31-33; 1564.90 Da (Bc), fractions 34–37; 1556.31 (Bd), fractions 38–40; 1570.11 Da (Be), fractions 41–44; 1584.62 Da (Bf), fractions 45–51; 1585.44 (Bg) and 1598.19 Da (Bh) and fraction 57; 1274.02 and 1223.92 Da (Bi).
(TIF)

S1 Table Primers used in this study. (A) Primers and PCR conditions used for identification of isolates with antimicrobial activity. (B) Primers and PCR conditions used for screening cosmid libraries of BGSP7, BGSP9 and BGSP11. (C) Primers and conditions used in RT-qPCR.
(DOCX)

S2 Table List of genes specific for each strain of *Br. laterosporus* isolated in this study (A) List of genes present in *Br. laterosporus* BGSP7 genome which are absent in BGSP9 and BGSP11 genomes. (B) List of genes present in *Br. laterosporus* BGSP9 genome which are absent in BGSP7 and BGSP11 genomes. (C) List of genes present in *Br. laterosporus* BGSP11 genome which are absent BGSP7 and BGSP9 genomes.
(DOCX)
