# Supplementary figures and images for: Brevibacillus laterosporus strains BGSP7, BGSP9 and BGSP11 isolated from silage produce broad spectrum multi-antimicrobials
Source: PLoS One. 2019 May 10;14(5):e0216773. doi: 10.1371/journal.pone.0216773 (PMC6510442; doi:10.1371/journal.pone.0216773)

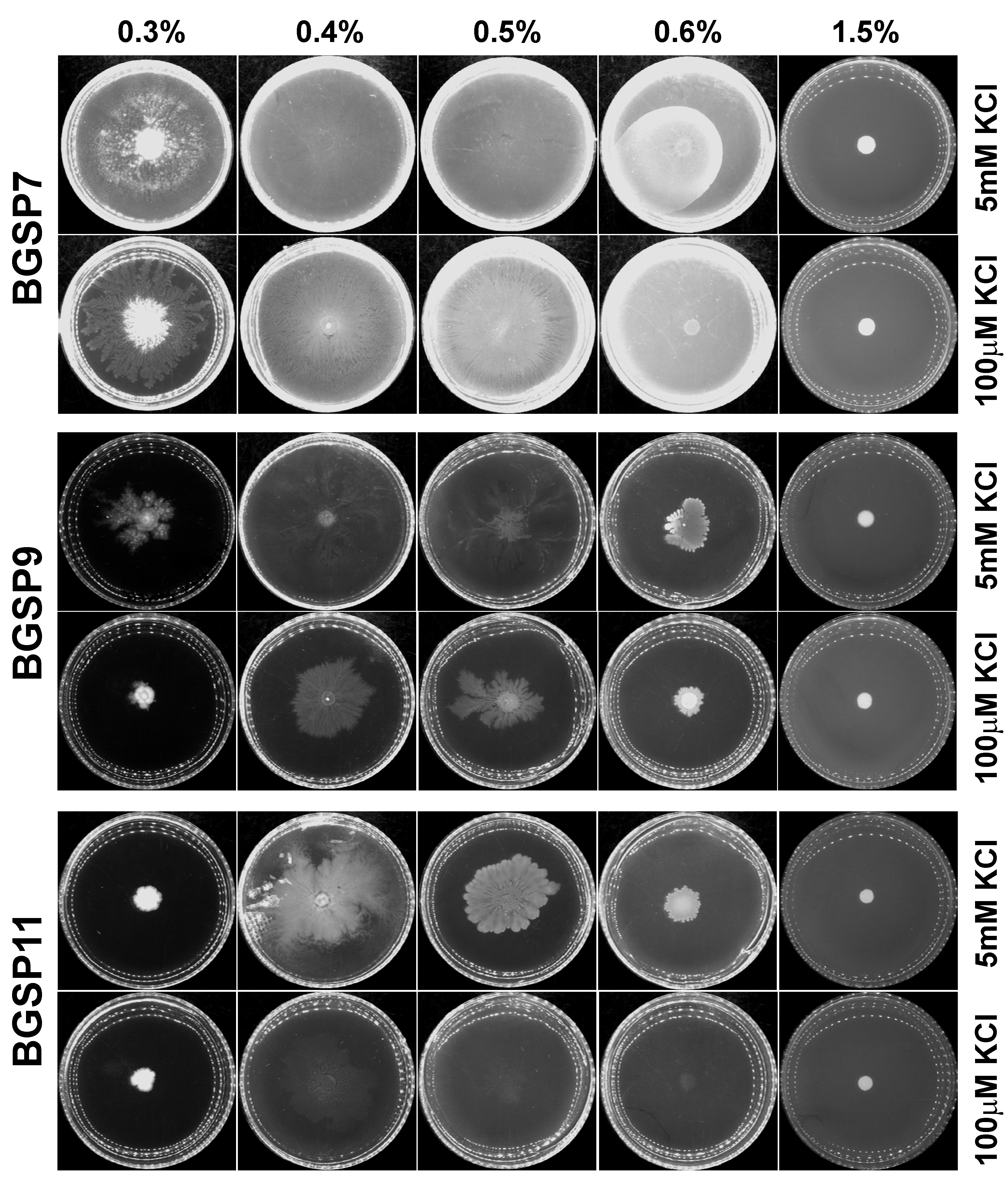

Supplement: S1 Fig — Semi-solid MSggN agarose plates (0.3 to 1.5% w/v agarose and 100 μM or 5 mM KCl) were inoculated in the center with 2μl of culture of the strains (in triplicate). After growth for 24 h at 37°C, typical plates were photographed. (TIF) [file pone.0216773.s001.tif]

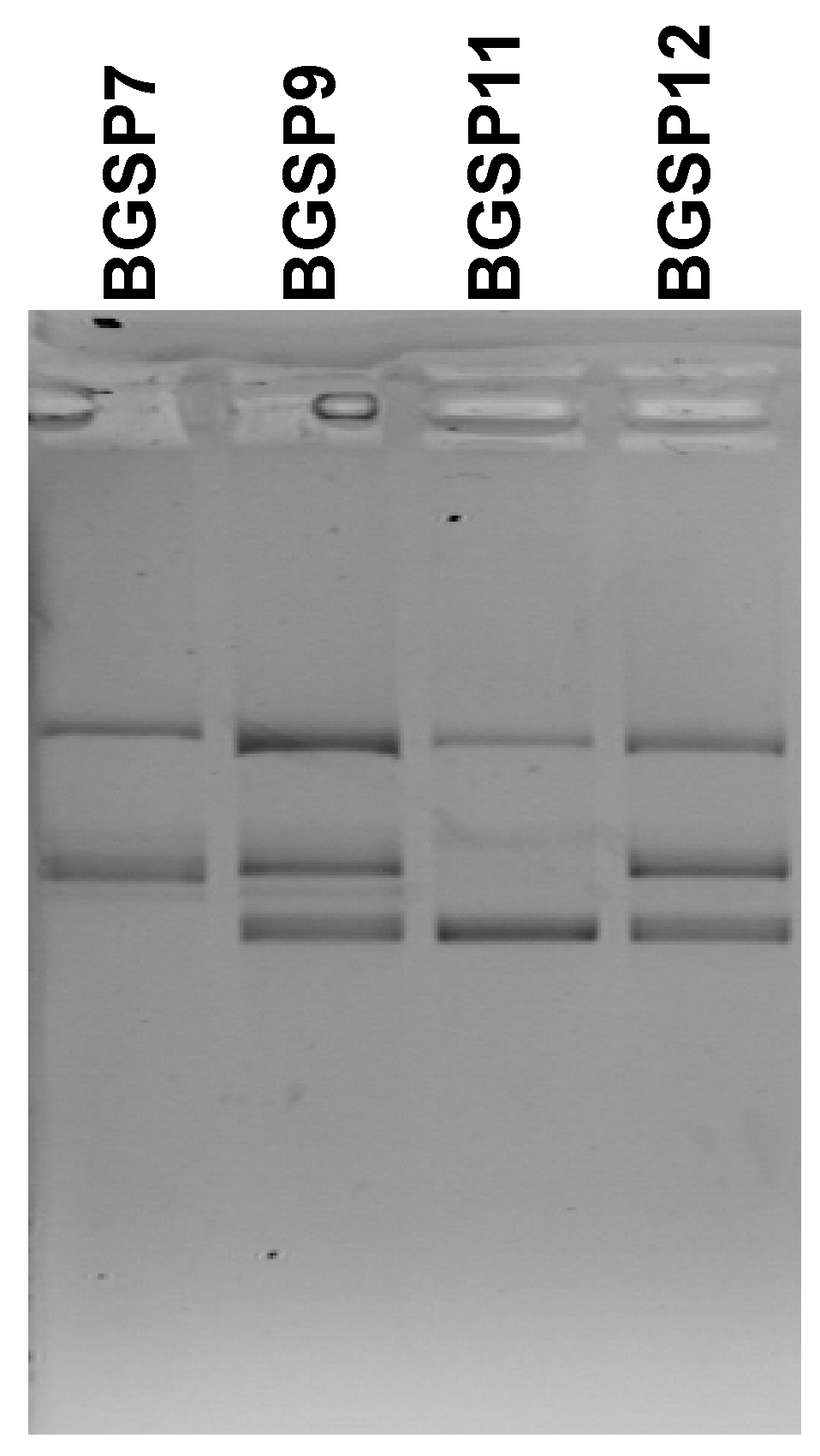

Supplement: S2 Fig — (TIF) [file pone.0216773.s002.tif]

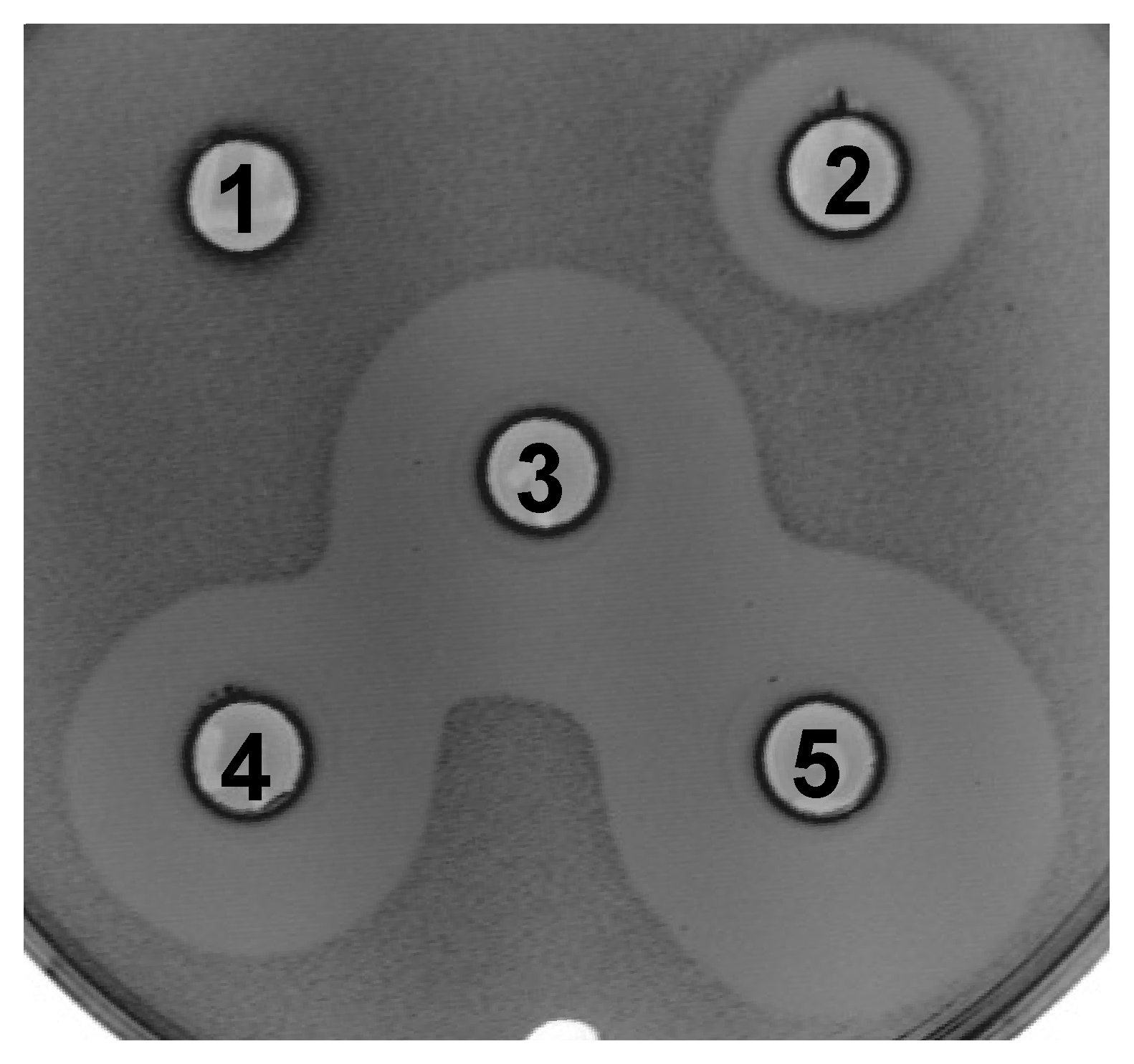

Supplement: S3 Fig — Antimicrobial activity of Br. laterosporus BGSP7 (3), BGSP9 (4) and BGSP11 (5) strains on P. larvae PC19726256. Negative control P. larvae PC19726256 (1) and nisin producer L. lactis (2) as positive control, were used. (TIF) [file pone.0216773.s003.tif]

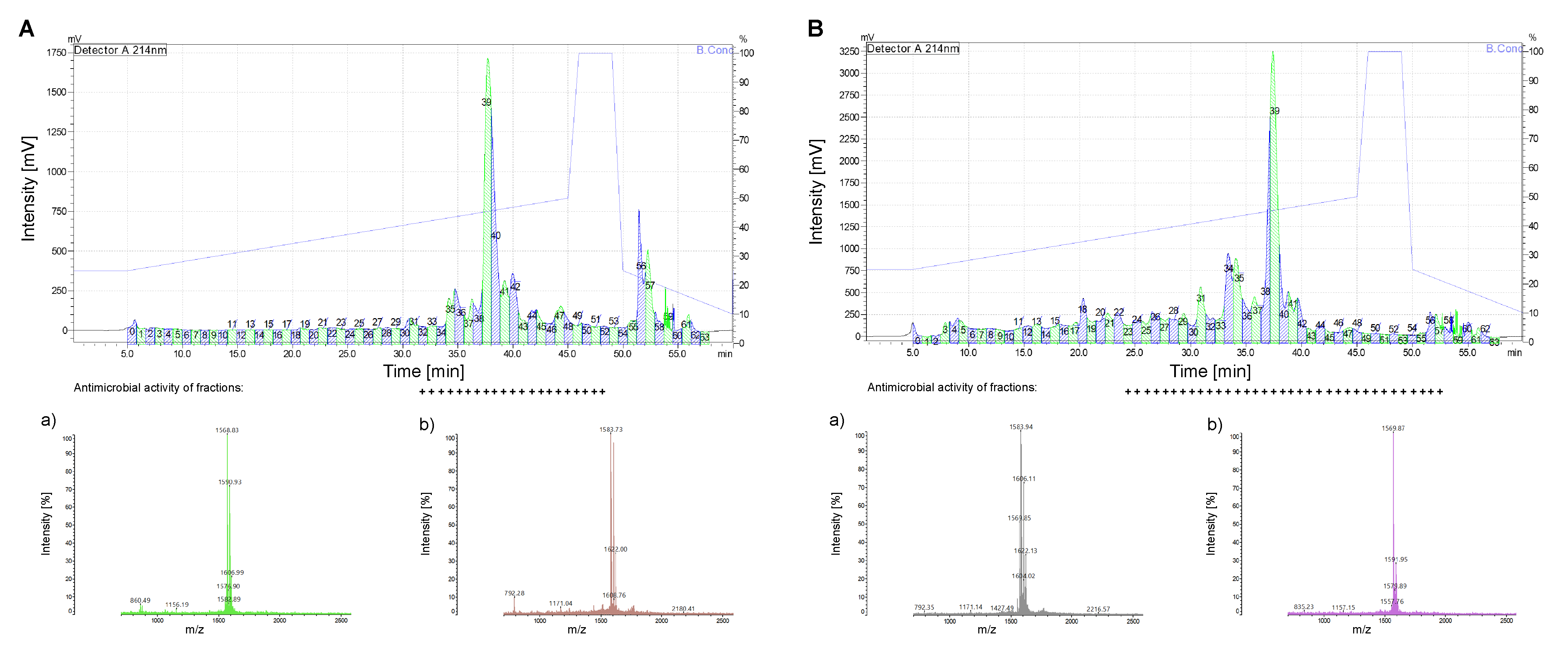

Supplement: S4 Fig — Matrix-assisted laser desorption ionization–time of flight (MALDI-TOF) mass spectrometry data from the fractions 33–36; 1568.83 Da (Aa), fractions 37–41; 1583.73 Da (Ab), fractions 29–42; 1583.94 Da (Ba), fractions 56–57; 1569.87 (Bb). (TIF) [file pone.0216773.s004.tif]

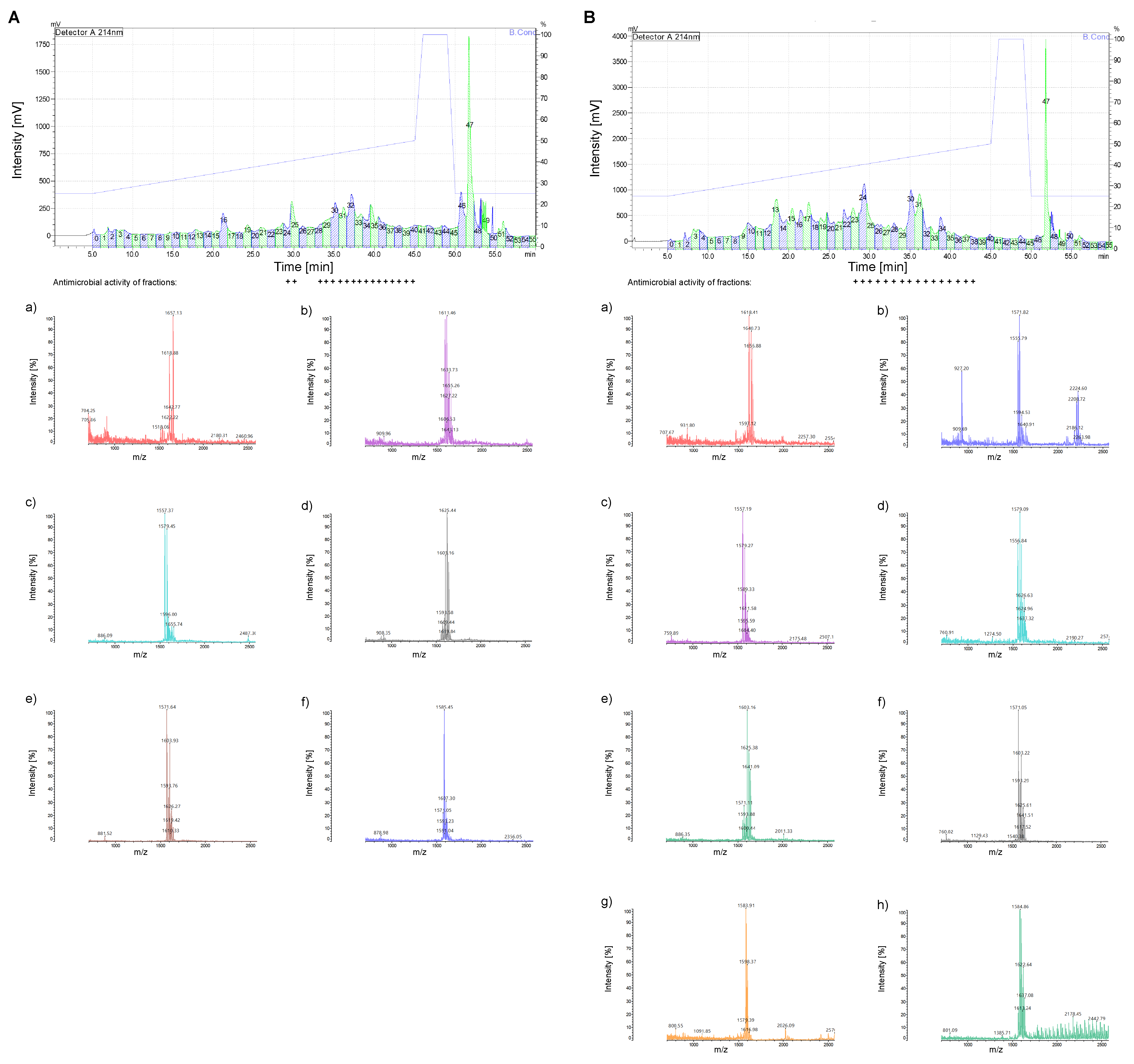

Supplement: S5 Fig — Matrix-assisted laser desorption ionization–time of flight (MALDI-TOF) mass spectrometry data from the fractions 24–25; 1657.53 Da (Aa), fraction 28; 1611.46 Da (Ab), fraction 29; 1557.37 Da (Ac), fraction 30; 1625.44 and 1603.16 Da (Ad), fractions 32–33; 1571.64 Da (Ae), fractions 34–36; 1585.45 Da (Af), fraction 24; 1618.41 Da (Ba), fractions 25–27; 1571.82 and 1555.79 Da (Bb), fraction 28; 1557.19 Da (Bc), fractions 29–30; 1579.09,1556.84 (Bd) and 1603.16 Da (Be), fractions 31–32; 1571.05 and 1603.22 Da (Bf), fractions 33–36; 1583.91 Da (Bg) and fraction 37; 1584.86 Da (Bh). (TIF) [file pone.0216773.s005.tif]

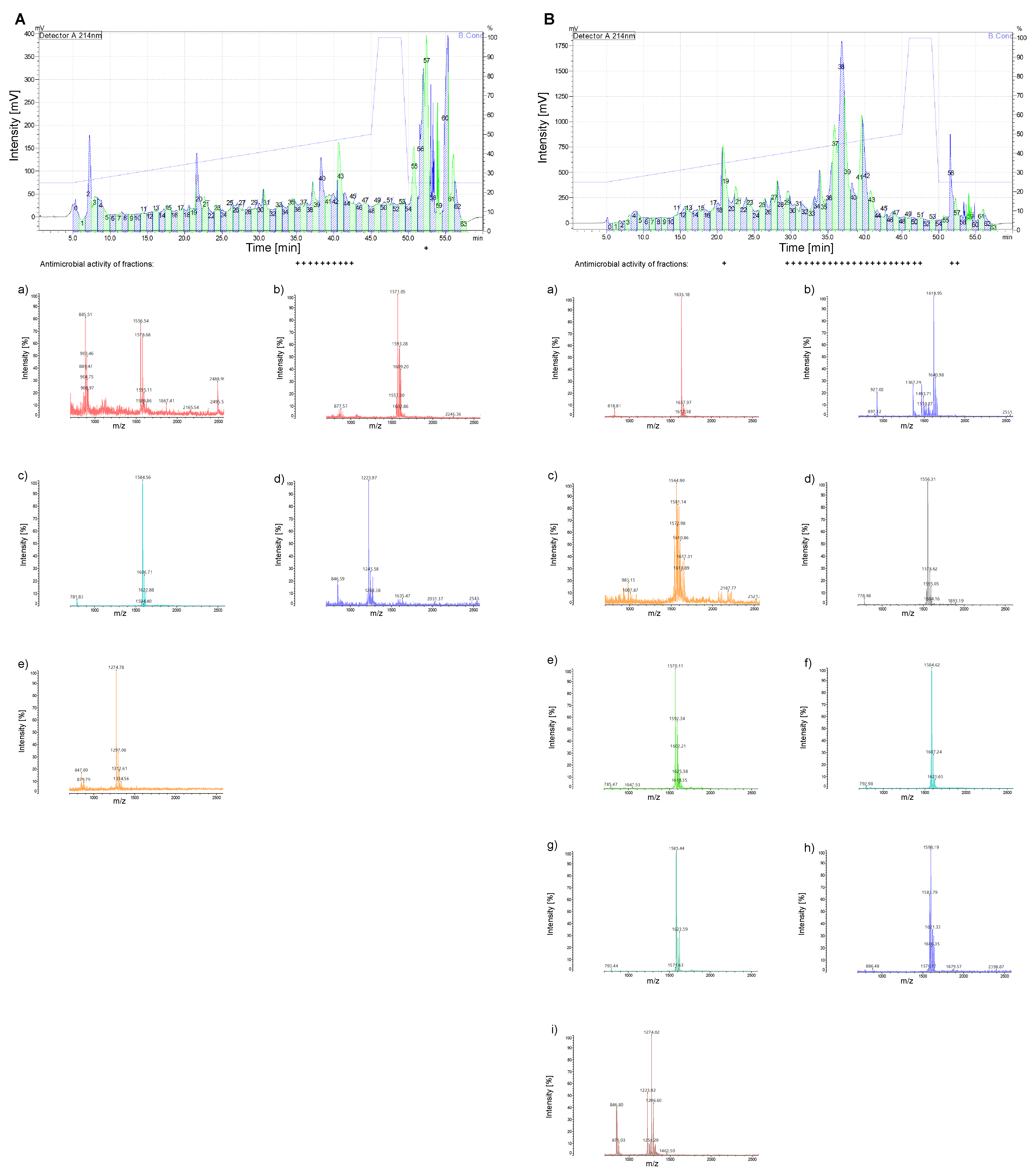

Supplement: S6 Fig — Matrix-assisted laser desorption ionization–time of flight (MALDI-TOF) mass spectrometry data from the fraction 36; 1556.54 Da (Aa), fractions 37–41; 1571.05 Da (Ab), fractions 42–44; 1584.56 Da (Ac), fractions 56–57; 1223.97 (Ad) and 1274.78 Da (Ae), fraction 19; 1636.18 Da (Ba), fraction 29–30; 1618.95 Da (Bb), fractions31-33; 1564.90 Da (Bc), fractions 34–37; 1556.31 (Bd), fractions 38–40; 1570.11 Da (Be), fractions 41–44; 1584.62 Da (Bf), fractions 45–51; 1585.44 (Bg) and 1598.19 Da (Bh) and fraction 57; 1274.02 and 1223.92 Da (Bi). (TIF) [file pone.0216773.s006.tif]
