# Supplementary material for: Brevibacillus laterosporus strains BGSP7, BGSP9 and BGSP11 isolated from silage produce broad spectrum multi-antimicrobials
Source: PLoS One. 2019 May 10;14(5):e0216773. doi: 10.1371/journal.pone.0216773 (PMC6510442; doi:10.1371/journal.pone.0216773)
Supplement: S1 Table — (A) Primers and PCR conditions used for identification of isolates with antimicrobial activity. (B) Primers and PCR conditions used for screening cosmid libraries of BGSP7, BGSP9 and BGSP11. (C) Primers and conditions used in RT-qPCR. (DOCX) [file pone.0216773.s007.docx]

**S1 Table**. Primers used in this study. (A) Primers and PCR conditions used for identification of isolates with antimicrobial activity. (B) Primers and PCR conditions used for screening cosmid libraries of BGSP7, BGSP9 and BGSP11. (C) Primers and conditions used in RT-qPCR.

**A)**

| **Name of primers** | **Sequence (5' - 3')** | **Template** | **Cycling conditions** |
| --- | --- | --- | --- |
| 16S - Fw | GAATCTTCCACAATGGACG | Genomic DNA isolated from BGSP7, BGSP9 and BGSP11 | 94°C/30 sek  55°C/30 sek  72°C/60 sek  [30 cycles] |
| 16S - Rev | TGACGGGCGGTGTGTACAAG |  |  |

**B)**

| **Name of primers** | **Sequence (5' - 3')** | **Template** | **Cycling conditions** |
| --- | --- | --- | --- |
| Laterosporulin - Fw | CCTTCCCTTCTGTATTTCC | Cosmid library of BGSP7/BGSP9/BGSP11 | 95°C/30 sek  50°C/30 sek  72°C/60 sek  [30 cycles] |
| Laterosporulin - Rev | GTGTAATCCCGTGCACCAC |  |  |
| Lactococcin 972-like - Fw | CAACTCTAATGTGACTCCG | Cosmid library of BGSP7/BGSP9/BGSP11 |  |
| Lactococcin 972-like - Rev | CATTTTCATAACGCCGATCC |  |  |

**C)**

| **Name of primers** | **Sequence (5' - 3')** | **Template** | **Cycling conditions** |
| --- | --- | --- | --- |
| rpoB - Fw | GAGACGGGCGAAGTGAAGGAGC | BGSP7, BGSP9 and BGSP11 | 95°C/3 min  95°C/15 sek  60°C/60 sek  [40 cycles] |
| rpoB - Rev | GTGTTATAGTAAACACTAGGGG |  |  |
| Linocin 972 – M18 Fw | ATGGATAAATCACAGAAATTCCC | BGSP7, BGSP9 and BGSP11 |  |
| Linocin 972 – M18 Rev | GTAATGGTTTGAATACCTTCTCC |  |  |
| Sactipeptide Fw | ATGAAAAATTATACAACACC | BGSP7, BGSP9 and BGSP11 |  |
| Sactipeptide Rev | CGCTCCTGCTCCATTTTTTTG |  |  |
| UviB2 Fw | TTTGTATGGCTATTGTTTTC | BGSP7, BGSP9 and BGSP11 |  |
| UviB2 Rev | GCCCACCTTTTCTTTTAG |  |  |
| UviB1 Fw | GCTCCAACAAGGTCCGTTTGC | BGSP7, BGSP9 and BGSP11 |  |
| UvuB1 Rev | GTGCTCCATGAGCTTAGCC |  |  |
| Lantipeptide Fw | GGATGTACAGGTGAAAGAGG | BGSP9 |  |
| Lantipeptide Rev | TTAGCAGCAGTTACTTTGGC |  |  |
| LAPs –Fw | GGGTGGAGATGTATCGCCTTGGG | BGSP7, BGSP9 and BGSP11 |  |
| LAPs – Rev | GACTGCATCGGAAGCAATTGATG |  |  |
| Linocin M18 –Fw | ATGGATAAATCACAGAAATTCCC | BGSP7, BGSP9 and BGSP11 |  |
| Linocin M18 –Rev | GTAATGGTTTGAATACCTTCTCC |  |  |
| Laterosporulin7 – Fw | ATGGCATGCGCAAATCAATGTCC | BGSP7 |  |
| Laterosporulin7 – Rev | TCTAGCATTTTGTGCTCC |  |  |
| Laterosporulin – Fw | ATGGCATGCGTAAATCAATGTCC | BGSP9 and BGSP11 |  |
| Laterosporulin – Rev | TCTAGCATTTTGTGCTCC |  |  |
