# Supplementary material for: Brevibacillus laterosporus strains BGSP7, BGSP9 and BGSP11 isolated from silage produce broad spectrum multi-antimicrobials
Source: PLoS One. 2019 May 10;14(5):e0216773. doi: 10.1371/journal.pone.0216773 (PMC6510442; doi:10.1371/journal.pone.0216773)
Supplement: S2 Table — List of genes specific for each strain of Br. laterosporus isolated in this study (A) List of genes present in Br. laterosporus BGSP7 genome which are absent in BGSP9 and BGSP11 genomes. (B) List of genes present in Br. laterosporus BGSP9 genome which are absent in BGSP7 and BGSP11 genomes. (C) List of genes present in Br. laterosporus BGSP11 genome which are absent BGSP7 and BGSP9 genomes. (DOCX) [file pone.0216773.s008.docx]

**S2 Table.** List of genes specific for each strain of *Br. laterosporus* isolated in this study. (A) List of genes present in *Br. laterosporus* BGSP7 genome which are absent in BGSP9 and BGSP11 genomes. (B) List of genes present in *Br. laterosporus* BGSP9 genome which are absent in BGSP7 and BGSP11 genomes. (C) List of genes present in *Br. laterosporus* BGSP11 genome which are absent BGSP7 and BGSP9 genomes.

**A)**

| **No** | **Scaffold** | **Start position** | **End position** | **Length** | **ID** | **Function** |
| --- | --- | --- | --- | --- | --- | --- |
| 1 | scf_19168_10 | 165431 | 165898 | 468 | fig\|1465.24.peg.336 | hypothetical protein |
| 2 | scf_19168_10 | 167051 | 167587 | 537 | fig\|1465.24.peg.337 | Syd protein |
| 3 | scf_19168_11 | 161670 | 161816 | 147 | fig\|1465.24.peg.521 | hypothetical protein |
| 4 | scf_19168_11 | 162189 | 163283 | 1095 | fig\|1465.24.peg.522 | probable secreted protein homolog of yjcM/yhbB B. subtilis |
| 5 | scf_19168_11 | 163364 | 164824 | 1461 | fig\|1465.24.peg.523 | FIG006789: Stage V sporulation protein |
| 6 | scf_19168_12 | 15333 | 16793 | 1461 | fig\|1465.24.peg.554 | hypothetical protein |
| 7 | scf_19168_12 | 16814 | 17215 | 402 | fig\|1465.24.peg.555 | hypothetical protein |
| 8 | scf_19168_12 | 80026 | 80889 | 864 | fig\|1465.24.peg.612 | Site-specific recombinase XerD |
| 9 | scf_19168_13 | 140063 | 141691 | 1629 | fig\|1465.24.peg.844 | FIG00674096: hypothetical protein |
| 10 | scf_19168_13 | 141739 | 142842 | 1104 | fig\|1465.24.peg.845 | DNA-cytosine methyltransferase (EC2.1.1.37); Ontology_term=KEGG_ENZYME:2.1.1.37 |
| 11 | scf_19168_13 | 143083 | 144024 | 942 | fig\|1465.24.peg.846 | hypothetical protein |
| 12 | scf_19168_13 | 168549 | 168701 | 153 | fig\|1465.24.peg.873 | hypothetical protein |
| 13 | scf_19168_13 | 168784 | 168897 | 114 | fig\|1465.24.peg.874 | hypothetical protein |
| 14 | scf_19168_13 | 168921 | 169832 | 912 | fig\|1465.24.peg.875 | hypothetical protein |
| 15 | scf_19168_14 | 157934 | 159088 | 1155 | fig\|1465.24.peg.1035 | hypothetical protein |
| 16 | scf_19168_14 | 159091 | 160032 | 942 | fig\|1465.24.peg.1036 | hypothetical protein |
| 17 | scf_19168_14 | 160036 | 161052 | 1017 | fig\|1465.24.peg.1037 | Lmo0572 protein |
| 18 | scf_19168_14 | 35752 | 36138 | 387 | fig\|1465.24.peg.913 | hypothetical protein |
| 19 | scf_19168_14 | 37162 | 37716 | 555 | fig\|1465.24.peg.914 | hypothetical protein |
| 20 | scf_19168_14 | 37834 | 38070 | 237 | fig\|1465.24.peg.915 | hypothetical protein |
| 21 | scf_19168_14 | 38394 | 38522 | 129 | fig\|1465.24.peg.916 | hypothetical protein |
| 22 | scf_19168_14 | 52369 | 52914 | 546 | fig\|1465.24.peg.934 | LysR family transcriptional regulator STM2281 |
| 23 | scf_19168_14 | 85120 | 85266 | 147 | fig\|1465.24.peg.969 | hypothetical protein |
| 24 | scf_19168_14 | 85338 | 86354 | 1017 | fig\|1465.24.peg.970 | hypothetical protein |
| 25 | scf_19168_15 | 14617 | 16485 | -1869 | fig\|1465.24.peg.1052 | Name=Tn7-like transposition protein D |
| 26 | scf_19168_15 | 16466 | 17998 | -1533 | fig\|1465.24.peg.1053 | FIG01239805: hypothetical protein |
| 27 | scf_19168_15 | 18346 | 19452 | 1107 | fig\|1465.24.peg.1054 | DNA-cytosine methyltransferase (EC2.1.1.37);  Ontology_term=KEGG_ENZYME:2.1.1.37 |
| 28 | scf_19168_15 | 19490 | 20554 | 1065 | fig\|1465.24.peg.1055 | hypothetical protein |
| 29 | scf_19168_15 | 20547 | 21617 | 1071 | fig\|1465.24.peg.1056 | hypothetical protein |
| 30 | scf_19168_15 | 22242 | 22481 | 240 | fig\|1465.24.peg.1057 | hypothetical protein |
| 31 | scf_19168_15 | 22552 | 22719 | 168 | fig\|1465.24.peg.1058 | hypothetical protein |
| 32 | scf_19168_15 | 26328 | 26441 | 114 | fig\|1465.24.peg.1064 | hypothetical protein |
| 33 | scf_19168_15 | 26755 | 27324 | 570 | fig\|1465.24.peg.1065 | Type I restriction-modification system specificit y subunit |
| 34 | scf_19168_15 | 27344 | 28936 | 1593 | fig\|1465.24.peg.1066 | Type I restriction-modification system, DNA-methyltransferase subunit M (EC2.1.1.72); Ontology_term=KEGG_ENZYME:2.1.1.72 |
| 35 | scf_19168_15 | 28926 | 30164 | 1239 | fig\|1465.24.peg.1067 | Type I restriction-modification system, specificity subunit S (EC3.1.21.3); Ontology_term=KEGG_ENZYME:3.1.21.3 |
| 36 | scf_19168_15 | 30188 | 33349 | 3162 | fig\|1465.24.peg.1068 | Type I restriction-modification system, restriction subunit R (EC3.1.21.3); Ontology_term=KEGG_ENZYME:3.1.21.3 |
| 37 | scf_19168_15 | 35422 | 35544 | 123 | fig\|1465.24.peg.1070 | hypothetical protein |
| 38 | scf_19168_15 | 35797 | 36519 | 723 | fig\|1465.24.peg.1071 | hypothetical protein |
| 39 | scf_19168_15 | 36594 | 36770 | 177 | fig\|1465.24.peg.1072 | hypothetical protein |
| 40 | scf_19168_15 | 36777 | 36893 | 117 | fig\|1465.24.peg.1073 | hypothetical protein |
| 41 | scf_19168_16 | 10061 | 10069 | 9 | fig\|1465.24.peg.1183 | Mobile element protein |
| 42 | scf_19168_16 | 9376 | 9420 | 45 | fig\|1465.24.peg.1183 | Mobile element protein |
| 43 | scf_19168_16 | 9941 | 10057 | 117 | fig\|1465.24.peg.1183 | Mobile element protein |
| 44 | scf_19168_18 | 3269 | 3445 | 177 | fig\|1465.24.peg.1445 | hypothetical protein |
| 45 | scf_19168_19 | 100238 | 100765 | 528 | fig\|1465.24.peg.1653 | hypothetical protein |
| 46 | scf_19168_19 | 105575 | 106054 | 480 | fig\|1465.24.peg.1660 | conserved membrane protein |
| 47 | scf_19168_19 | 106091 | 106789 | 699 | fig\|1465.24.peg.1661 | Predicted N-ribosylNicotinamide CRP-like regulator |
| 48 | scf_19168_19 | 98775 | 98918 | 144 | fig\|1465.24.peg.1651 | hypothetical protein |
| 49 | scf_19168_19 | 99022 | 100080 | 1059 | fig\|1465.24.peg.1652 | hypothetical protein |
| 50 | scf_19168_1 | 10075 | 10317 | 243 | fig\|1465.24.peg.17 | hypothetical protein |
| 51 | scf_19168_1 | 10363 | 11109 | 747 | fig\|1465.24.peg.18 | hypothetical protein |
| 52 | scf_19168_1 | 11133 | 11288 | 156 | fig\|1465.24.peg.19 | hypothetical protein |
| 53 | scf_19168_1 | 13262 | 13540 | 279 | fig\|1465.24.peg.25 | hypothetical protein |
| 54 | scf_19168_1 | 1345 | 1698 | 354 | fig\|1465.24.peg.2 | Mobile element protein |
| 55 | scf_19168_1 | 13955 | 14089 | 135 | fig\|1465.24.peg.26 | hypothetical protein |
| 56 | scf_19168_1 | 158 | 1048 | 891 | fig\|1465.24.peg.1 | hypothetical protein |
| 57 | scf_19168_1 | 15902 | 16336 | 435 | fig\|1465.24.peg.28 | transcriptional regulator, MarR family |
| 58 | scf_19168_1 | 16391 | 17587 | 1197 | fig\|1465.24.peg.29 | penicillin-binding protein, putative |
| 59 | scf_19168_1 | 1709 | 2431 | 723 | fig\|1465.24.peg.3 | Mobile element protein |
| 60 | scf_19168_1 | 17836 | 17970 | 135 | fig\|1465.24.peg.30 | hypothetical protein |
| 61 | scf_19168_1 | 18021 | 18191 | 171 | fig\|1465.24.peg.31 | hypothetical protein |
| 62 | scf_19168_1 | 18834 | 19022 | 189 | fig\|1465.24.peg.32 | hypothetical protein |
| 63 | scf_19168_1 | 20882 | 23695 | 2814 | fig\|1465.24.peg.38 | Ribonucleotide reductase of class II (coenzyme B12- dependent) (EC1.17.4.1); Ontology_term=KEGG_ENZYME:1.17.4.1 |
| 64 | scf_19168_1 | 218188 | 219081 | 894 | fig\|1465.24.peg.128 | hypothetical protein |
| 65 | scf_19168_1 | 219137 | 219775 | 639 | fig\|1465.24.peg.129 | HNH endonuclease |
| 66 | scf_19168_1 | 2428 | 3681 | 1254 | fig\|1465.24.peg.4 | Mobile element protein |
| 67 | scf_19168_1 | 27484 | 27894 | 411 | fig\|1465.24.peg.45 | Phage protein |
| 68 | scf_19168_1 | 29778 | 30041 | 264 | fig\|1465.24.peg.50 | hypothetical protein |
| 69 | scf_19168_1 | 3685 | 4812 | -1128 | fig\|1465.24.peg.5 | FIG00533656: hypothetical protein |
| 70 | scf_19168_1 | 4815 | 4955 | 141 | fig\|1465.24.peg.6 | hypothetical protein |
| 71 | scf_19168_1 | 9316 | 9492 | 177 | fig\|1465.24.peg.15 | hypothetical protein |
| 72 | scf_19168_20 | 82860 | 83489 | 630 | fig\|1465.24.peg.2088 | hypothetical protein |
| 73 | scf_19168_21 | 11817 | 12464 | 648 | fig\|1465.24.peg.2142 | hypothetical protein |
| 74 | scf_19168_21 | 12994 | 13119 | 126 | fig\|1465.24.peg.2143 | hypothetical protein |
| 75 | scf_19168_21 | 13175 | 13918 | 744 | fig\|1465.24.peg.2144 | hypothetical protein |
| 76 | scf_19168_21 | 14729 | 14869 | 141 | fig\|1465.24.peg.2146 | hypothetical protein |
| 77 | scf_19168_21 | 14859 | 15215 | 357 | fig\|1465.24.peg.2147 | Transcriptional regulator |
| 78 | scf_19168_21 | 15367 | 15594 | 228 | fig\|1465.24.peg.2148 | Repressor (cro-like) [Bacteriophage A118] |
| 79 | scf_19168_21 | 17468 | 17602 | 135 | fig\|1465.24.peg.2153 | hypothetical protein |
| 80 | scf_19168_21 | 17962 | 18744 | 783 | fig\|1465.24.peg.2154 | Protein gp49 replication initiation [Bacteriop hage A118] |
| 81 | scf_19168_21 | 18735 | 19082 | 348 | fig\|1465.24.peg.2155 | replication |
| 82 | scf_19168_21 | 19153 | 20442 | 1290 | fig\|1465.24.peg.2156 | Replicative DNA helicase (DnaB) (EC3.6.4.12);  Ontology_term=KEGG_ENZYME:3.6.4.12 |
| 83 | scf_19168_21 | 20709 | 21017 | 309 | fig\|1465.24.peg.2158 | hypothetical protein |
| 84 | scf_19168_21 | 22321 | 22569 | 249 | fig\|1465.24.peg.2163 | hypothetical protein |
| 85 | scf_19168_21 | 24817 | 24843 | 27 | fig\|1465.24.peg.2169 | prophage LambdaBa01 positive control factor Xpf |
| 86 | scf_19168_21 | 25628 | 26293 | 666 | fig\|1465.24.peg.2171 | hypothetical protein |
| 87 | scf_19168_21 | 26433 | 27836 | 1404 | fig\|1465.24.peg.2172 | hypothetical protein |
| 88 | scf_19168_21 | 27937 | 28284 | 348 | fig\|1465.24.peg.2173 | Phage-associated homing endonuclease |
| 89 | scf_19168_21 | 28386 | 28700 | -315 | fig\|1465.24.peg.2174 | Phage terminase, small subunit |
| 90 | scf_19168_21 | 28678 | 30414 | -1737 | fig\|1465.24.peg.2175 | Phage terminase, large subunit |
| 91 | scf_19168_21 | 30433 | 31641 | 1209 | fig\|1465.24.peg.2176 | Phage portal protein |
| 92 | scf_19168_21 | 31625 | 32392 | -768 | fig\|1465.24.peg.2177 | Prophage Clp protease-like protein |
| 93 | scf_19168_21 | 32389 | 33522 | 1134 | fig\|1465.24.peg.2178 | Phage major capsid protein |
| 94 | scf_19168_21 | 33538 | 33807 | 270 | fig\|1465.24.peg.2179 | hypothetical protein |
| 95 | scf_19168_21 | 33820 | 34116 | 297 | fig\|1465.24.peg.2180 | Phage protein |
| 96 | scf_19168_21 | 47274 | 47669 | 396 | fig\|1465.24.peg.2201 | Phage holin |
| 97 | scf_19168_21 | 48425 | 49030 | 606 | fig\|1465.24.peg.2203 | hypothetical protein |
| 98 | scf_19168_21 | 49276 | 49512 | 237 | fig\|1465.24.peg.2204 | hypothetical protein |
| 99 | scf_19168_21 | 73612 | 74328 | 717 | fig\|1465.24.peg.2230 | hypothetical protein |
| 100 | scf_19168_21 | 81401 | 82501 | -1101 | fig\|1465.24.peg.2234 | FIG01228564: hypothetical protein |
| 101 | scf_19168_22 | 94043 | 94351 | 309 | fig\|1465.24.peg.2304 | Transposase |
| 102 | scf_19168_22 | 94472 | 94612 | 141 | fig\|1465.24.peg.2305 | Mobile element protein |
| 103 | scf_19168_22 | 94616 | 94708 | 93 | fig\|1465.24.peg.2305 | Mobile element protein |
| 104 | scf_19168_22 | 97403 | 98047 | 645 | fig\|1465.24.peg.2308 | hypothetical protein |
| 105 | scf_19168_22 | 98084 | 98245 | 162 | fig\|1465.24.peg.2309 | hypothetical protein |
| 106 | scf_19168_23 | 36972 | 37112 | 141 | fig\|1465.24.peg.2352 | hypothetical protein |
| 107 | scf_19168_23 | 37535 | 38086 | 552 | fig\|1465.24.peg.2353 | hypothetical protein |
| 108 | scf_19168_23 | 38124 | 38273 | 150 | fig\|1465.24.peg.2354 | hypothetical protein |
| 109 | scf_19168_24 | 2669 | 3856 | -1188 | fig\|1465.24.peg.2420 | FIG01225154: hypothetical protein |
| 110 | scf_19168_24 | 283 | 558 | 276 | fig\|1465.24.peg.2415 | hypothetical protein |
| 111 | scf_19168_24 | 66679 | 66726 | 48 | fig\|1465.24.peg.2470 | Arsenate reductase (EC1.20.4.1);Ontology_term=KEGG_ENZYME:1.20.4.1 |
| 112 | scf_19168_25 | 2282 | 2401 | 120 | fig\|1465.24.peg.2482 | hypothetical protein |
| 113 | scf_19168_26 | 10006 | 10290 | -285 | fig\|1465.24.peg.2537 | FIG01229470: hypothetical protein |
| 114 | scf_19168_26 | 8473 | 8589 | 117 | fig\|1465.24.peg.2535 | hypothetical protein |
| 115 | scf_19168_26 | 8940 | 9920 | 981 | fig\|1465.24.peg.2536 | Cell wall surface anchor family protein |
| 116 | scf_19168_27 | 24088 | 24249 | -162 | fig\|1465.24.peg.2646 | membrane protein, putative |
| 117 | scf_19168_28 | 1861 | 1998 | 138 | fig\|1465.24.peg.2691 | hypothetical protein |
| 118 | scf_19168_28 | 2849 | 3070 | 222 | fig\|1465.24.peg.2693 | Mobile element protein |
| 119 | scf_19168_28 | 3982 | 4374 | 393 | fig\|1465.24.peg.2694 | Bona fide RidA/YjgF/TdcF/RutC subgroup |
| 120 | scf_19168_28 | 6936 | 7079 | 144 | fig\|1465.24.peg.2696 | hypothetical protein |
| 121 | scf_19168_28 | 79661 | 79819 | 159 | fig\|1465.24.peg.2764 | hypothetical protein |
| 122 | scf_19168_3 | 153410 | 153574 | 165 | fig\|1465.24.peg.3011 | hypothetical protein |
| 123 | scf_19168_41 | 5383 | 6750 | 1368 | fig\|1465.24.peg.3870 | hypothetical protein |
| 124 | scf_19168_48 | 1506 | 1667 | 162 | fig\|1465.24.peg.3911 | hypothetical protein |
| 125 | scf_19168_49 | 280 | 495 | 216 | fig\|1465.24.peg.3913 | hypothetical protein |
| 126 | scf_19168_49 | 939 | 1532 | 594 | fig\|1465.24.peg.3914 | hypothetical protein |
| 127 | scf_19168_4 | 120627 | 121187 | 561 | fig\|1465.24.peg.3697 | hypothetical protein |
| 128 | scf_19168_4 | 121335 | 121574 | 240 | fig\|1465.24.peg.3698 | hypothetical protein |
| 129 | scf_19168_4 | 135505 | 138042 | 2538 | fig\|1465.24.peg.3724 | prophage LambdaBa01 acyltransferase |
| 130 | scf_19168_4 | 138365 | 138586 | 222 | fig\|1465.24.peg.3725 | hypothetical protein |
| 131 | scf_19168_4 | 148879 | 149517 | 639 | fig\|1465.24.peg.3739 | hypothetical protein |
| 132 | scf_19168_4 | 152827 | 152964 | 138 | fig\|1465.24.peg.3744 | hypothetical protein |
| 133 | scf_19168_4 | 153258 | 154271 | 1014 | fig\|1465.24.peg.3745 | Mosquitocidal toxin |
| 134 | scf_19168_4 | 155386 | 155601 | 216 | fig\|1465.24.peg.3747 | hypothetical protein |
| 135 | scf_19168_4 | 156000 | 156524 | 525 | fig\|1465.24.peg.3748 | hypothetical protein |
| 136 | scf_19168_4 | 260126 | 260239 | 114 | fig\|1465.24.peg.3824 | hypothetical protein |
| 137 | scf_19168_4 | 263299 | 263535 | 237 | fig\|1465.24.peg.3828 | hypothetical protein |
| 138 | scf_19168_4 | 263959 | 264399 | 441 | fig\|1465.24.peg.3829 | hypothetical protein |
| 139 | scf_19168_4 | 268261 | 268479 | 219 | fig\|1465.24.peg.3833 | hypothetical protein |
| 140 | scf_19168_57 | 54 | 614 | 561 | fig\|1465.24.peg.4141 | hypothetical protein |
| 141 | scf_19168_5 | 10565 | 10684 | 120 | fig\|1465.24.peg.3931 | Flagellar motor rotation protein MotB |
| 142 | scf_19168_5 | 10717 | 10911 | 195 | fig\|1465.24.peg.3932 | methyl-accepting chemotaxis protein, putative |
| 143 | scf_19168_5 | 12526 | 12774 | 249 | fig\|1465.24.peg.3934 | hypothetical protein |
| 144 | scf_19168_5 | 154599 | 154901 | 303 | fig\|1465.24.peg.4049 | Lipoprotein, putative |
| 145 | scf_19168_5 | 191803 | 192510 | 708 | fig\|1465.24.peg.4088 | hypothetical protein |
| 146 | scf_19168_5 | 2787 | 3158 | 372 | fig\|1465.24.peg.3918 | hypothetical protein |
| 147 | scf_19168_5 | 3784 | 3909 | 126 | fig\|1465.24.peg.3919 | hypothetical protein |
| 148 | scf_19168_5 | 4127 | 4888 | 762 | fig\|1465.24.peg.3920 | Methyltransferase (EC 2.1.1.-); Ontology_term=KEGG_ENZYME:2.1.1.- |
| 149 | scf_19168_5 | 5203 | 5376 | 174 | fig\|1465.24.peg.3921 | DNA-invertase |
| 150 | scf_19168_5 | 5456 | 5827 | 372 | fig\|1465.24.peg.3922 | Transcriptional repressor, BlaI/MecI family |
| 151 | scf_19168_5 | 54679 | 54822 | 144 | fig\|1465.24.peg.3952 | hypothetical protein |
| 152 | scf_19168_5 | 5820 | 6872 | 1053 | fig\|1465.24.peg.3923 | Regulatory sensor-transducer, BlaR1/MecR1 family |
| 153 | scf_19168_5 | 66861 | 67451 | 591 | fig\|1465.24.peg.3965 | hypothetical protein |
| 154 | scf_19168_5 | 6872 | 7696 | 825 | fig\|1465.24.peg.3924 | hypothetical protein |
| 155 | scf_19168_5 | 73910 | 74155 | 246 | fig\|1465.24.peg.3974 | hypothetical protein |
| 156 | scf_19168_5 | 7994 | 8452 | 459 | fig\|1465.24.peg.3925 | Name=Endonuclease |
| 157 | scf_19168_5 | 82756 | 83187 | -432 | fig\|1465.24.peg.3979 | NADPH:quinone oxidoreductase 2 |
| 158 | scf_19168_5 | 8420 | 8983 | 564 | fig\|1465.24.peg.3926 | hypothetical protein |
| 159 | scf_19168_5 | 87377 | 87772 | 396 | fig\|1465.24.peg.3985 | hypothetical protein |
| 160 | scf_19168_5 | 8952 | 9281 | 330 | fig\|1465.24.peg.3927 | hypothetical protein |
| 161 | scf_19168_5 | 90909 | 91781 | -873 | fig\|1465.24.peg.3990 | Cys-tRNA(Pro) deacylase YbaK |
| 162 | scf_19168_5 | 91915 | 92490 | 576 | fig\|1465.24.peg.3991 | LysE |
| 163 | scf_19168_5 | 9248 | 9454 | 207 | fig\|1465.24.peg.3928 | hypothetical protein |
| 164 | scf_19168_60 | 322 | 441 | 120 | fig\|1465.24.peg.4331 | hypothetical protein |
| 165 | scf_19168_69 | 221 | 421 | 201 | fig\|1465.24.peg.4332 | tRNA (Guanine37-N1) -methyltransferase (EC2.1.1.31);  Ontology_term=KEGG_ENZYME:2.1.1.31 |
| 166 | scf_19168_6 | 201335 | 202294 | 960 | fig\|1465.24.peg.4323 | PROBABLE CONSERVED LIPOPROTEIN LPQO |
| 167 | scf_19168_78 | 123 | 281 | 159 | fig\|1465.24.peg.4544 | conserved domain protein |
| 168 | scf_19168_7 | 16481 | 17065 | 585 | fig\|1465.24.peg.4350 | hypothetical protein |
| 169 | scf_19168_7 | 17124 | 17921 | 798 | fig\|1465.24.peg.4351 | hypothetical protein |
| 170 | scf_19168_7 | 18435 | 18620 | 186 | fig\|1465.24.peg.4353 | hypothetical protein |
| 171 | scf_19168_7 | 86322 | 86666 | 345 | fig\|1465.24.peg.4428 | Possible uridine kinase (EC 2.7.1.48);Ontology_term=KEGG_ENZYME:2.7.1.48 |
| 172 | scf_19168_82 | 24 | 278 | -255 | fig\|1465.24.peg.4741 | FIG00553873: hypothetical protein |
| 173 | scf_19168_84 | 242 | 367 | 126 | fig\|1465.24.peg.4742 | hypothetical protein |
| 174 | scf_19168_85 | 138 | 338 | 201 | fig\|1465.24.peg.4743 | hypothetical protein |
| 175 | scf_19168_86 | 244 | 399 | 156 | fig\|1465.24.peg.4744 | COG2110 Macro domain, possibly ADP-ribose binding module |
| 176 | scf_19168_87 | 24 | 155 | 132 | fig\|1465.24.peg.4745 | Low molecular weight protein-tyrosine-phosphatase Wzb (EC3.1.3.48); Ontology_term=KEGG_ENZYME:3.1.3.48 |
| 177 | scf_19168_9 | 11334 | 12011 | 678 | fig\|1465.24.peg.4761 | Phage-associated DNA primase (EC 2.7.7.-); Ontology_term=KEGG_ENZYME:2.7.7.- |
| 178 | scf_19168_9 | 115010 | 115186 | 177 | fig\|1465.24.peg.4887 | hypothetical protein |
| 179 | scf_19168_9 | 115511 | 115816 | 306 | fig\|1465.24.peg.4888 | Mobile element protein |
| 180 | scf_19168_9 | 12044 | 13510 | -1467 | fig\|1465.24.peg.4762 | DNA helicase phage-associated |
| 181 | scf_19168_9 | 13524 | 14129 | 606 | fig\|1465.24.peg.4763 | Phage protein |
| 182 | scf_19168_9 | 136 | 738 | 603 | fig\|1465.24.peg.4747 | hypothetical protein |
| 183 | scf_19168_9 | 14192 | 15163 | 972 | fig\|1465.24.peg.4764 | Phage protein |
| 184 | scf_19168_9 | 15217 | 16314 | 1098 | fig\|1465.24.peg.4765 | Phage protein |
| 185 | scf_19168_9 | 16884 | 18080 | 1197 | fig\|1465.24.peg.4766 | hypothetical protein |
| 186 | scf_19168_9 | 19645 | 20712 | 1068 | fig\|1465.24.peg.4769 | Adenine-specific methyltransferase (EC 2.1.1.72); Ontology_term=KEGG_ENZYME:2.1.1.72 |
| 187 | scf_19168_9 | 23513 | 23629 | 117 | fig\|1465.24.peg.4778 | hypothetical protein |
| 188 | scf_19168_9 | 24284 | 24523 | 240 | fig\|1465.24.peg.4780 | hypothetical protein |
| 189 | scf_19168_9 | 26498 | 26926 | 429 | fig\|1465.24.peg.4784 | hypothetical protein |
| 190 | scf_19168_9 | 26973 | 27518 | 546 | fig\|1465.24.peg.4785 | hypothetical protein |
| 191 | scf_19168_9 | 4355 | 4624 | 270 | fig\|1465.24.peg.4754 | Phage protein |
| 192 | scf_19168_9 | 5492 | 5662 | 171 | fig\|1465.24.peg.4756 | hypothetical protein |
| 193 | scf_19168_9 | 6793 | 9912 | 3120 | fig\|1465.24.peg.4759 | DNA polymerase III alpha subunit (EC 2.7.7.7); Ontology_term=KEGG_ENZYME:2.7.7.7 |
| 194 | scf_19168_9 | 76527 | 76964 | 438 | fig\|1465.24.peg.4843 | hypothetical protein |
| 195 | scf_19168_9 | 77153 | 77731 | 579 | fig\|1465.24.peg.4844 | hypothetical protein |
| 196 | scf_19168_9 | 78859 | 78993 | 135 | fig\|1465.24.peg.4847 | hypothetical protein |
| 197 | scf_19168_9 | 9917 | 11020 | 1104 | fig\|1465.24.peg.4760 | Phage protein |

**B)**

| **No** | **Scaffold** | **Start position** | **End position** | **Length** | **ID** | **Function** |
| --- | --- | --- | --- | --- | --- | --- |
| 1 | scf_20796_13 | 10546 | 10962 | 417 | fig\|1465.25.peg.660 | hypothetical protein |
| 2 | scf_20796_13 | 10965 | 11330 | 366 | fig\|1465.25.peg.661 | hypothetical protein |
| 3 | scf_20796_13 | 11331 | 11714 | 384 | fig\|1465.25.peg.662 | hypothetical protein |
| 4 | scf_20796_13 | 11733 | 11951 | 219 | fig\|1465.25.peg.663 | hypothetical protein |
| 5 | scf_20796_13 | 11963 | 13081 | 1119 | fig\|1465.25.peg.664 | Phage protein |
| 6 | scf_20796_13 | 1267 | 1845 | 579 | fig\|1465.25.peg.650 | phage-like element pbsx protein XkdT |
| 7 | scf_20796_13 | 13144 | 13839 | 696 | fig\|1465.25.peg.665 | Phage capsid and scaffold |
| 8 | scf_20796_13 | 13911 | 14738 | 828 | fig\|1465.25.peg.666 | Rhs family protein |
| 9 | scf_20796_13 | 14738 | 16066 | 1329 | fig\|1465.25.peg.667 | FIG01245128: hypothetical protein |
| 10 | scf_20796_13 | 16306 | 18006 | 1701 | fig\|1465.25.peg.668 | FIG01238653: hypothetical protein |
| 11 | scf_20796_13 | 1838 | 2911 | 1074 | fig\|1465.25.peg.651 | Phage-like element PBSX protein xkdT |
| 12 | scf_20796_13 | 20100 | 20732 | 633 | fig\|1465.25.peg.671 | hypothetical protein |
| 13 | scf_20796_13 | 21010 | 21870 | 861 | fig\|1465.25.peg.672 | hypothetical protein |
| 14 | scf_20796_13 | 22152 | 22508 | 357 | fig\|1465.25.peg.673 | hypothetical protein |
| 15 | scf_20796_13 | 23331 | 23450 | 120 | fig\|1465.25.peg.674 | hypothetical protein |
| 16 | scf_20796_13 | 24155 | 24604 | 450 | fig\|1465.25.peg.675 | BH0956 unknown |
| 17 | scf_20796_13 | 24604 | 24831 | 228 | fig\|1465.25.peg.676 | hypothetical protein |
| 18 | scf_20796_13 | 24984 | 26201 | 1218 | fig\|1465.25.peg.677 | DNA methylase |
| 19 | scf_20796_13 | 26202 | 27029 | 828 | fig\|1465.25.peg.678 | DNA-cytosine methyltransferase (EC2.1.1.37); Ontology_term=KEGG_ENZYME:2.1.1.37 |
| 20 | scf_20796_13 | 27094 | 27774 | 681 | fig\|1465.25.peg.679 | DNA-cytosine methyltransferase (EC2.1.1.37);Ontology_term=KEGG_ENZYME:2.1.1.37 |
| 21 | scf_20796_13 | 28140 | 28265 | 126 | fig\|1465.25.peg.680 | hypothetical protein |
| 22 | scf_20796_13 | 28391 | 28936 | 546 | fig\|1465.25.peg.681 | adenylate kinase and related kinases |
| 23 | scf_20796_13 | 28929 | 30209 | 1281 | fig\|1465.25.peg.682 | hypothetical protein |
| 24 | scf_20796_13 | 2914 | 3330 | 417 | fig\|1465.25.peg.652 | Phage-like element PBSX protein xkdS |
| 25 | scf_20796_13 | 30237 | 31073 | 837 | fig\|1465.25.peg.683 | Thymidylate synthase thyX (EC 2.1.1.-); Ontology_term=KEGG_ENZYME:2.1.1.- |
| 26 | scf_20796_13 | 31264 | 31839 | 576 | fig\|1465.25.peg.684 | Deoxyuridine 5'-triphosphate nucleotidohydrolase (EC 3.6.1.23);Ontology_term=KEGG_ENZYME:3.6.1.23 |
| 27 | scf_20796_13 | 32341 | 34569 | 2229 | fig\|1465.25.peg.685 | Ribonucleotide reductase of class II (coenzyme B12 -dependent) (EC1.17.4.1);  Ontology_term=KEGG_ENZYME:1.17.4.1 |
| 28 | scf_20796_13 | 3320 | 3466 | 147 | fig\|1465.25.peg.653 | hypothetical protein |
| 29 | scf_20796_13 | 34930 | 35091 | 162 | fig\|1465.25.peg.686 | hypothetical protein |
| 30 | scf_20796_13 | 35293 | 35577 | 285 | fig\|1465.25.peg.687 | Phage protein |
| 31 | scf_20796_13 | 35580 | 36104 | 525 | fig\|1465.25.peg.688 | hypothetical protein |
| 32 | scf_20796_13 | 36566 | 37153 | 588 | fig\|1465.25.peg.689 | DNA polymerase III epsilon subunit (EC2.7.7.7);Ontology_term=KEGG_ENZYME:2.7.7.7 |
| 33 | scf_20796_13 | 37153 | 38121 | 969 | fig\|1465.25.peg.690 | hypothetical protein |
| 34 | scf_20796_13 | 38139 | 38441 | 303 | fig\|1465.25.peg.691 | hypothetical protein |
| 35 | scf_20796_13 | 38444 | 39574 | 1131 | fig\|1465.25.peg.692 | DNA polymerase I (EC2.7.7.7); Ontology_term=KEGG_ENZYME:2.7.7.7 |
| 36 | scf_20796_13 | 43498 | 43653 | 156 | fig\|1465.25.peg.697 | hypothetical protein |
| 37 | scf_20796_13 | 43671 | 44426 | 756 | fig\|1465.25.peg.698 | hypothetical protein |
| 38 | scf_20796_13 | 44456 | 44848 | 393 | fig\|1465.25.peg.699 | hypothetical protein |
| 39 | scf_20796_13 | 45251 | 45805 | 555 | fig\|1465.25.peg.700 | hypothetical protein |
| 40 | scf_20796_13 | 46859 | 47089 | 231 | fig\|1465.25.peg.702 | hypothetical protein |
| 41 | scf_20796_13 | 47329 | 47472 | 144 | fig\|1465.25.peg.703 | hypothetical protein |
| 42 | scf_20796_13 | 47474 | 48799 | 1326 | fig\|1465.25.peg.704 | hypothetical protein |
| 43 | scf_20796_13 | 4790 | 5464 | 675 | fig\|1465.25.peg.655 | Phage-like element PBSX protein xkdP |
| 44 | scf_20796_13 | 48964 | 49182 | 219 | fig\|1465.25.peg.705 | hypothetical protein |
| 45 | scf_20796_13 | 49155 | 50168 | 1014 | fig\|1465.25.peg.706 | DNA primase |
| 46 | scf_20796_13 | 50182 | 51525 | 1344 | fig\|1465.25.peg.707 | hypothetical protein |
| 47 | scf_20796_13 | 51545 | 52345 | 801 | fig\|1465.25.peg.708 | Phage antirepressor protein |
| 48 | scf_20796_13 | 52571 | 52753 | 183 | fig\|1465.25.peg.709; | hypothetical protein |
| 49 | scf_20796_13 | 52750 | 53520 | 771 | fig\|1465.25.peg.710 | Helicase loader DnaI |
| 50 | scf_20796_13 | 53741 | 54121 | 381 | fig\|1465.25.peg.711 | hypothetical protein |
| 51 | scf_20796_13 | 54108 | 54494 | 387 | fig\|1465.25.peg.712 | hypothetical protein |
| 52 | scf_20796_13 | 54628 | 55020 | 393 | fig\|1465.25.peg.713 | hypothetical protein |
| 53 | scf_20796_13 | 55017 | 55325 | 309 | fig\|1465.25.peg.714 | hypothetical protein |
| 54 | scf_20796_13 | 55322 | 55564 | 243 | fig\|1465.25.peg.715 | hypothetical protein |
| 55 | scf_20796_13 | 55599 | 55811 | 213 | fig\|1465.25.peg.716 | hypothetical protein |
| 56 | scf_20796_13 | 57281 | 58303 | 1023 | fig\|1465.25.peg.718 | Chromosome (plasmid) partitioning protein ParB |
| 57 | scf_20796_13 | 59396 | 59686 | 291 | fig\|1465.25.peg.719 | hypothetical protein |
| 58 | scf_20796_13 | 5962 | 7971 | 2010 | fig\|1465.25.peg.656 | Phage tail length tape-measure protein |
| 59 | scf_20796_13 | 8144 | 8509 | 366 | fig\|1465.25.peg.657 | hypothetical protein |
| 60 | scf_20796_13 | 8573 | 9019 | 447 | fig\|1465.25.peg.658 | hypothetical protein |
| 61 | scf_20796_13 | 9034 | 10071 | 1038 | fig\|1465.25.peg.659 | Phage-like element PBSX protein xkdK |
| 62 | scf_20796_150 | 227 | 343 | 117 | fig\|1465.25.peg.984 | Nucleoside-diphosphate-sugar epimerases |
| 63 | scf_20796_156 | 352 | 412 | 61 | fig\|1465.25.rna.27 | tRNA-Val-CAC |
| 64 | scf_20796_156 | 352 | 412 | 61 | fig\|1465.25.rna.27 | tRNA-Val-CAC |
| 65 | scf_20796_156 | 352 | 412 | 61 | fig\|1465.25.rna.27 | tRNA-Val-CAC |
| 66 | scf_20796_15 | 116722 | 117294 | 573 | fig\|1465.25.peg.977 | hypothetical protein |
| 67 | scf_20796_162 | 30 | 191 | 162 | fig\|1465.25.peg.1067 | Isovaleryl-CoA dehydrogenase (EC1.3.8.4);Ontology_term=KEGG_ENZYME:1.3.8.4 |
| 68 | scf_20796_170 | 53 | 376 | -324 | fig\|1465.25.peg.1109 | FIG00639119: hypothetical protein |
| 69 | scf_20796_172 | 44 | 211 | 168 | fig\|1465.25.peg.1110 | Putative Na(+)/H(+) exchanger protein, CPA1 family precursor |
| 70 | scf_20796_17 | 23162 | 23290 | 129 | fig\|1465.25.peg.1083 | Metal-dependent hydrolase |
| 71 | scf_20796_17 | 496 | 648 | 153 | fig\|1465.25.peg.1068 | hypothetical protein |
| 72 | scf_20796_1 | 129666 | 130247 | 582 | fig\|1465.25.peg.128 | Transcription antitermination protein NusG |
| 73 | scf_20796_1 | 135171 | 139838 | 4668 | fig\|1465.25.peg.131 | Malonyl CoA-acyl carrier protein transacylase (EC2.3.1.39); Ontology_term=KEGG_ENZYME:2.3.1.39 |
| 74 | scf_20796_1 | 139868 | 150655 | 10788 | fig\|1465.25.peg.132 | Malonyl CoA-acyl carrier protein transacylase (EC2.3.1.39); Ontology_term=KEGG_ENZYME:2.3.1.39 |
| 75 | scf_20796_1 | 150679 | 158673 | 7995 | fig\|1465.25.peg.133 | Malonyl CoA-acyl carrier protein transacylase (EC2.3.1.39); Ontology_term=KEGG_ENZYME:2.3.1.39 |
| 76 | scf_20796_1 | 196339 | 196587 | 249 | fig\|1465.25.peg.137 | Acyl carrier protein |
| 77 | scf_20796_1 | 196556 | 197791 | 1236 | fig\|1465.25.peg.138 | 3-oxoacyl-[acyl-carrier-protein] synthase KASII (EC 2.3.1.179); Ontology_term=KEGG_ENZYME:2.3.1.179 |
| 78 | scf_20796_1 | 197816 | 199075 | 1260 | fig\|1465.25.peg.139 | Hydroxymethylglutaryl-CoA synthase (EC 2.3.3.10);  Ontology_term=KEGG_ENZYME:2.3.3.10 |
| 79 | scf_20796_1 | 199075 | 199839 | 765 | fig\|1465.25.peg.140 | Methylglutaconyl-CoA hydratase (EC 4.2.1.18); Ontol ogy_term=KEGG_ENZYME:4.2.1.18 |
| 80 | scf_20796_1 | 199846 | 200589 | 744 | fig\|1465.25.peg.141 | Enoyl-CoA hydratase (EC 4.2.1.17);Ontology_term=KEGG_ENZYME:4.2.1.17 |
| 81 | scf_20796_1 | 231806 | 231979 | 174 | fig\|1465.25.peg.145 | hypothetical protein |
| 82 | scf_20796_1 | 232077 | 232226 | 150 | fig\|1465.25.peg.146 | hypothetical protein |
| 83 | scf_20796_1 | 75188 | 75478 | 291 | fig\|1465.25.peg.83 | hypothetical protein |
| 84 | scf_20796_1 | 85897 | 86733 | 837 | fig\|1465.25.peg.89 | hypothetical protein |
| 85 | scf_20796_1 | 86926 | 87426 | 501 | fig\|1465.25.peg.90 | Phage protein |
| 86 | scf_20796_1 | 87462 | 88070 | 609 | fig\|1465.25.peg.91 | Phage protein |
| 87 | scf_20796_1 | 88086 | 90257 | 2172 | fig\|1465.25.peg.92 | hypothetical protein |
| 88 | scf_20796_1 | 90303 | 91967 | 1665 | fig\|1465.25.peg.93 | Phage protein |
| 89 | scf_20796_1 | 91983 | 94301 | 2319 | fig\|1465.25.peg.94 | unnamed protein product |
| 90 | scf_20796_22 | 15107 | 15832 | -726 | fig\|1465.25.peg.1810 | N-acetylmuramoyl-L-alanine amidase |
| 91 | scf_20796_22 | 1676 | 2299 | 624 | fig\|1465.25.peg.1783 | Phage-like element PBSX protein xkdA |
| 92 | scf_20796_22 | 16969 | 17310 | 342 | fig\|1465.25.peg.1813 | hypothetical protein |
| 93 | scf_20796_22 | 17394 | 18161 | 768 | fig\|1465.25.peg.1814 | Similar to terminase small subunit yqaS homolog |
| 94 | scf_20796_22 | 190 | 1665 | -1476 | fig\|1465.25.peg.1782 | Integrase [Bacteriophage A118] |
| 95 | scf_20796_22 | 21777 | 22814 | 1038 | fig\|1465.25.peg.1818 | Phage minor capsid protein |
| 96 | scf_20796_22 | 23937 | 24977 | 1041 | fig\|1465.25.peg.1821 | Phage major capsid protein |
| 97 | scf_20796_22 | 25028 | 25192 | 165 | fig\|1465.25.peg.1822 | hypothetical protein |
| 98 | scf_20796_22 | 25192 | 25551 | 360 | fig\|1465.25.peg.1823 | hypothetical protein |
| 99 | scf_20796_22 | 25551 | 25907 | 357 | fig\|1465.25.peg.1824 | hypothetical protein |
| 100 | scf_20796_22 | 26827 | 27003 | 177 | fig\|1465.25.peg.1827 | hypothetical protein |
| 101 | scf_20796_22 | 3135 | 3263 | 129 | fig\|1465.25.peg.1786 | hypothetical protein |
| 102 | scf_20796_22 | 3355 | 3954 | 600 | fig\|1465.25.peg.1787 | hypothetical protein |
| 103 | scf_20796_22 | 3969 | 4184 | 216 | fig\|1465.25.peg.1788 | hypothetical protein |
| 104 | scf_20796_22 | 4174 | 4347 | 174 | fig\|1465.25.peg.1789 | hypothetical protein |
| 105 | scf_20796_22 | 4396 | 4644 | 249 | fig\|1465.25.peg.1790 | hypothetical protein |
| 106 | scf_20796_22 | 4628 | 4885 | 258 | fig\|1465.25.peg.1791 | hypothetical protein |
| 107 | scf_20796_22 | 4882 | 5367 | 486 | fig\|1465.25.peg.1792 | Phage protein |
| 108 | scf_20796_22 | 5378 | 5974 | 597 | fig\|1465.25.peg.1793 | hypothetical protein |
| 109 | scf_20796_22 | 5967 | 6386 | 420 | fig\|1465.25.peg.1794 | Single-stranded DNA-binding protein |
| 110 | scf_20796_22 | 7303 | 7653 | 351 | fig\|1465.25.peg.1797 | hypothetical protein |
| 111 | scf_20796_22 | 8646 | 9380 | -735 | fig\|1465.25.peg.1799 | Phage antirepressor protein |
| 112 | scf_20796_28 | 72705 | 72893 | 189 | fig\|1465.25.peg.2382 | hypothetical protein |
| 113 | scf_20796_28 | 73093 | 74094 | 1002 | fig\|1465.25.peg.2383 | hypothetical protein |
| 114 | scf_20796_28 | 75817 | 75945 | 129 | fig\|1465.25.peg.2385 | hypothetical protein |
| 115 | scf_20796_28 | 75949 | 76470 | 522 | fig\|1465.25.peg.2386 | hypothetical protein |
| 116 | scf_20796_28 | 76653 | 77030 | 378 | fig\|1465.25.peg.2387 | hypothetical protein |
| 117 | scf_20796_30 | 10387 | 11541 | 1155 | fig\|1465.25.peg.2656 | DNA polymerase III beta subunit (EC 2.7.7.7);Ontology_term=KEGG_ENZYME:2.7.7.7 |
| 118 | scf_20796_30 | 12139 | 13260 | 1122 | fig\|1465.25.peg.2657 | hypothetical protein |
| 119 | scf_20796_30 | 13505 | 13723 | 219 | fig\|1465.25.peg.2658 | hypothetical protein |
| 120 | scf_20796_30 | 15339 | 15584 | 246 | fig\|1465.25.peg.2660 | hypothetical protein |
| 121 | scf_20796_30 | 16239 | 16502 | 264 | fig\|1465.25.peg.2661 | hypothetical protein |
| 122 | scf_20796_30 | 17372 | 17869 | 498 | fig\|1465.25.peg.2663 | hypothetical protein |
| 123 | scf_20796_30 | 1752 | 2687 | 936 | fig\|1465.25.peg.2649 | N-acetylmuramoyl-L-alanine amidase (EC 3.5.1.28); Ontology_term=KEGG_ENZYME:3.5.1.28 |
| 124 | scf_20796_30 | 20126 | 20275 | 150 | fig\|1465.25.peg.2665 | hypothetical protein |
| 125 | scf_20796_30 | 20503 | 20880 | 378 | fig\|1465.25.peg.2666 | Transcriptional regulator MarR family, Cinnamoyl ester hydrolase |
| 126 | scf_20796_30 | 20981 | 21592 | 612 | fig\|1465.25.peg.2667 | Glutathione-regulated potassium-efflux system anc illary protein KefG |
| 127 | scf_20796_30 | 22023 | 22163 | 141 | fig\|1465.25.peg.2668 | hypothetical protein |
| 128 | scf_20796_30 | 22688 | 22957 | 270 | fig\|1465.25.peg.2669 | hypothetical protein |
| 129 | scf_20796_30 | 23489 | 23611 | 123 | fig\|1465.25.peg.2670 | hypothetical protein |
| 130 | scf_20796_30 | 23583 | 24782 | 1200 | fig\|1465.25.peg.2671 | hypothetical protein |
| 131 | scf_20796_30 | 24824 | 24940 | 117 | fig\|1465.25.peg.2672 | hypothetical protein |
| 132 | scf_20796_30 | 24959 | 26065 | 1107 | fig\|1465.25.peg.2673 | hypothetical protein |
| 133 | scf_20796_30 | 26281 | 27135 | 855 | fig\|1465.25.peg.2674 | hypothetical protein |
| 134 | scf_20796_30 | 27224 | 27364 | 141 | fig\|1465.25.peg.2675 | hypothetical protein |
| 135 | scf_20796_30 | 2740 | 3387 | -648 | fig\|1465.25.peg.2650 | Peptidase M23/M37 family |
| 136 | scf_20796_30 | 33524 | 34141 | 618 | fig\|1465.25.peg.2680 | hypothetical protein |
| 137 | scf_20796_30 | 41362 | 41691 | 330 | fig\|1465.25.peg.2686 | hypothetical protein |
| 138 | scf_20796_30 | 41757 | 42134 | 378 | fig\|1465.25.peg.2687 | hypothetical protein |
| 139 | scf_20796_30 | 42695 | 42874 | 180 | fig\|1465.25.peg.2688 | hypothetical protein |
| 140 | scf_20796_30 | 44710 | 45546 | 837 | fig\|1465.25.peg.2690 | hypothetical protein |
| 141 | scf_20796_30 | 46668 | 47264 | 597 | fig\|1465.25.peg.2692 | hypothetical protein |
| 142 | scf_20796_30 | 47248 | 47385 | 138 | fig\|1465.25.peg.2693 | hypothetical protein |
| 143 | scf_20796_30 | 5227 | 5358 | 132 | fig\|1465.25.peg.2652 | hypothetical protein |
| 144 | scf_20796_30 | 73676 | 73990 | 315 | fig\|1465.25.peg.2715 | hypothetical protein |
| 145 | scf_20796_39 | 29195 | 29455 | -261 | fig\|1465.25.peg.3238 | FIG01226489: hypothetical protein |
| 146 | scf_20796_40 | 37709 | 37882 | 174 | fig\|1465.25.peg.3473 | Mobile element protein |
| 147 | scf_20796_40 | 39518 | 40393 | 876 | fig\|1465.25.peg.3476 | probable hydrolase |
| 148 | scf_20796_40 | 41413 | 41541 | 129 | fig\|1465.25.peg.3477 | hypothetical protein |
| 149 | scf_20796_40 | 41567 | 41695 | 129 | fig\|1465.25.peg.3478 | hypothetical protein |
| 150 | scf_20796_40 | 41728 | 42945 | 1218 | fig\|1465.25.peg.3479 | Epoxide hydrolase (EC3.3.2.9);Ontology_term=KEGG_ENZYME:3.3.2.9 |
| 151 | scf_20796_40 | 42975 | 43418 | 444 | fig\|1465.25.peg.3480 | Bile acid 7-alpha dehydratase BaiE (EC 4.2.1.106); Ontology_term=KEGG_ENZYME:4.2.1.106 |
| 152 | scf_20796_40 | 43861 | 44460 | 600 | fig\|1465.25.peg.3481 | Transcriptional regulatorTetR family |
| 153 | scf_20796_43 | 11133 | 11438 | 306 | fig\|1465.25.peg.3591 | Bacteriophage |
| 154 | scf_20796_43 | 11444 | 11743 | 300 | fig\|1465.25.peg.3592 | hypothetical protein |
| 155 | scf_20796_43 | 11778 | 12938 | 1161 | fig\|1465.25.peg.3593 | Phage major capsid protein |
| 156 | scf_20796_43 | 12963 | 13709 | -747 | fig\|1465.25.peg.3594 | Prophage Clp protease-like protein |
| 157 | scf_20796_43 | 13702 | 14856 | 1155 | fig\|1465.25.peg.3595 | Phage portal protein |
| 158 | scf_20796_43 | 14875 | 16551 | -1677 | fig\|1465.25.peg.3596 | Phage terminase, large subunit |
| 159 | scf_20796_43 | 16553 | 16867 | 315 | fig\|1465.25.peg.3597 | Phage protein |
| 160 | scf_20796_43 | 16973 | 17173 | 201 | fig\|1465.25.peg.3598 | hypothetical protein |
| 161 | scf_20796_43 | 17167 | 17508 | -342 | fig\|1465.25.peg.3599 | FIG01108941: hypothetical protein |
| 162 | scf_20796_43 | 20578 | 21030 | 453 | fig\|1465.25.peg.3606 | hypothetical protein |
| 163 | scf_20796_43 | 21426 | 21665 | 240 | fig\|1465.25.peg.3608 | hypothetical protein |
| 164 | scf_20796_43 | 22803 | 23447 | 645 | fig\|1465.25.peg.3612 | Dimeric dUTPase (EC 3.6.1.23); Ontology_term=KEGG_ENZYME:3.6.1.23 |
| 165 | scf_20796_43 | 25187 | 25477 | 291 | fig\|1465.25.peg.3615 | hypothetical protein |
| 166 | scf_20796_43 | 27204 | 27416 | 213 | fig\|1465.25.peg.3620 | hypothetical protein |
| 167 | scf_20796_43 | 27956 | 28069 | 114 | fig\|1465.25.peg.3622 | hypothetical protein |
| 168 | scf_20796_43 | 28842 | 29357 | 516 | fig\|1465.25.peg.3624 | Accessory gene regulator B |
| 169 | scf_20796_43 | 29350 | 30006 | 657 | fig\|1465.25.peg.3625 | hypothetical protein |
| 170 | scf_20796_54 | 22725 | 23552 | 828 | fig\|1465.25.peg.4167 | hypothetical protein |
| 171 | scf_20796_54 | 23908 | 24642 | 735 | fig\|1465.25.peg.4168 | hypothetical protein |
| 172 | scf_20796_55 | 11444 | 11749 | 306 | fig\|1465.25.peg.4179 | hypothetical protein |
| 173 | scf_20796_55 | 11938 | 12873 | 936 | fig\|1465.25.peg.4180 | hypothetical protein |
| 174 | scf_20796_55 | 15674 | 16264 | 591 | fig\|1465.25.peg.4186 | hypothetical protein |
| 175 | scf_20796_55 | 22053 | 22925 | 873 | fig\|1465.25.peg.4197 | Transcriptional regulator AraC family |
| 176 | scf_20796_55 | 23465 | 23749 | 285 | fig\|1465.25.peg.4198 | hypothetical protein |
| 177 | scf_20796_55 | 24344 | 24478 | 135 | fig\|1465.25.peg.4200 | hypothetical protein |
| 178 | scf_20796_55 | 26492 | 26623 | 132 | fig\|1465.25.peg.4204 | hypothetical protein |
| 179 | scf_20796_55 | 2716 | 2859 | 144 | fig\|1465.25.peg.4172 | N-acetylmuramoyl-L-alanine amidase (EC 3.5.1.28); Ontology_term=KEGG_ENZYME:3.5.1.28 |
| 180 | scf_20796_55 | 3054 | 3176 | 123 | fig\|1465.25.peg.4173 | hypothetical protein |
| 181 | scf_20796_55 | 3734 | 3868 | 135 | fig\|1465.25.peg.4174 | hypothetical protein |
| 182 | scf_20796_55 | 3939 | 7139 | 3201 | fig\|1465.25.peg.4175 | Lanthionine biosynthesis protein LanB |
| 183 | scf_20796_57 | 19339 | 19506 | 168 | fig\|1465.25.peg.4243 | hypothetical protein |
| 184 | scf_20796_57 | 21465 | 21710 | 246 | fig\|1465.25.peg.4246 | hypothetical protein |
| 185 | scf_20796_57 | 21712 | 22473 | 762 | fig\|1465.25.peg.4247 | Flagellar protein FlgJ [peptidoglycan hydrolase] (EC3.2.1.-); Ontology_term=KEGG_ENZYME:3.2.1.- |
| 186 | scf_20796_57 | 22484 | 22747 | 264 | fig\|1465.25.peg.4248 | hypothetical protein |
| 187 | scf_20796_57 | 22757 | 22894 | 138 | fig\|1465.25.peg.4249 | hypothetical protein |
| 188 | scf_20796_57 | 22894 | 23094 | 201 | fig\|1465.25.peg.4250 | hypothetical protein |
| 189 | scf_20796_58 | 461 | 751 | 291 | fig\|1465.25.peg.4251 | Methyltransferase (EC 2.1.1.-); Ontology_term=KEGG_ENZYME:2.1.1.- |
| 190 | scf_20796_61 | 1365 | 2771 | 1407 | fig\|1465.25.peg.4493 | TnpA transposase |
| 191 | scf_20796_61 | 2791 | 3225 | 435 | fig\|1465.25.peg.4494 | TnpA transposase |
| 192 | scf_20796_61 | 3303 | 4328 | 1026 | fig\|1465.25.peg.4495 | TnpA transposase |
| 193 | scf_20796_64 | 1141 | 1332 | 192 | fig\|1465.25.peg.4542 | hypothetical protein |
| 194 | scf_20796_64 | 2835 | 3983 | 1149 | fig\|1465.25.peg.4544 | hypothetical protein |
| 195 | scf_20796_64 | 3970 | 7557 | 3588 | fig\|1465.25.peg.4545 | DNA helicase, putative |
| 196 | scf_20796_66 | 2739 | 2879 | 141 | fig\|1465.25.peg.4578 | hypothetical protein |
| 197 | scf_20796_66 | 3538 | 4128 | 591 | fig\|1465.25.peg.4580 | hypothetical protein |
| 198 | scf_20796_66 | 4197 | 4349 | 153 | fig\|1465.25.peg.4581 | N-acetylmuramoyl-L-alanine amidase (EC 3.5.1.28); Ontology_term=KEGG_ENZYME:3.5.1.28 |
| 199 | scf_20796_69 | 11839 | 12006 | 168 | fig\|1465.25.peg.4652 | hypothetical protein |
| 200 | scf_20796_69 | 12070 | 12624 | 555 | fig\|1465.25.peg.4653 | hypothetical protein |
| 201 | scf_20796_69 | 1817 | 1936 | 120 | fig\|1465.25.peg.4639 | hypothetical protein |
| 202 | scf_20796_69 | 3545 | 4189 | 645 | fig\|1465.25.peg.4643 | ankyrin repeat protein |
| 203 | scf_20796_69 | 4376 | 4726 | 351 | fig\|1465.25.peg.4644 | FIG131328: Predicted ATP-dependent endonuclease of the OLD family |
| 204 | scf_20796_69 | 4866 | 5606 | 741 | fig\|1465.25.peg.4645 | Putative deoxyribonuclease similar to YcfH, type 4 |
| 205 | scf_20796_70 | 10769 | 11380 | 612 | fig\|1465.25.peg.4843 | hypothetical protein |
| 206 | scf_20796_70 | 4287 | 4517 | 231 | fig\|1465.25.peg.4836 | hypothetical protein |
| 207 | scf_20796_70 | 4613 | 4846 | 234 | fig\|1465.25.peg.4837 | hypothetical protein |
| 208 | scf_20796_70 | 5176 | 6861 | 1686 | fig\|1465.25.peg.4838 | putative phage protein |
| 209 | scf_20796_70 | 6907 | 7998 | 1092 | fig\|1465.25.peg.4839 | putative phage protein |
| 210 | scf_20796_70 | 8316 | 8507 | 192 | fig\|1465.25.peg.4840 | hypothetical protein |
| 211 | scf_20796_71 | 6294 | 6440 | 147 | fig\|1465.25.peg.4853 | hypothetical protein |
| 212 | scf_20796_71 | 6770 | 6889 | 120 | fig\|1465.25.peg.4854 | hypothetical protein |
| 213 | scf_20796_72 | 1027 | 1143 | 117 | fig\|1465.25.peg.4865 | hypothetical protein |
| 214 | scf_20796_72 | 663 | 905 | 243 | fig\|1465.25.peg.4864 | hypothetical protein |
| 215 | scf_20796_73 | 53 | 904 | 852 | fig\|1465.25.peg.4876 | Antirestriction protein |
| 216 | scf_20796_73 | 6850 | 6984 | 135 | fig\|1465.25.peg.4883 | hypothetical protein |
| 217 | scf_20796_73 | 7012 | 7581 | 570 | fig\|1465.25.peg.4884 | hypothetical protein |
| 218 | scf_20796_73 | 9095 | 9532 | 438 | fig\|1465.25.peg.4886 | hypothetical protein |
| 219 | scf_20796_75 | 1228 | 1362 | 135 | fig\|1465.25.peg.4896 | hypothetical protein |
| 220 | scf_20796_75 | 1450 | 1914 | 465 | fig\|1465.25.peg.4897 | Mobile element protein |
| 221 | scf_20796_75 | 187 | 1065 | 879 | fig\|1465.25.peg.4895 | Helix-turn-helix AraC type: Bacterial transcription activator effector binding |
| 222 | scf_20796_75 | 2070 | 2246 | 177 | fig\|1465.25.peg.4898 | hypothetical protein |
| 223 | scf_20796_78 | 2983 | 3756 | 774 | fig\|1465.25.peg.4918 | hypothetical protein |
| 224 | scf_20796_79 | 1491 | 1658 | 168 | fig\|1465.25.peg.4923 | hypothetical protein |
| 225 | scf_20796_85 | 2947 | 3195 | 249 | fig\|1465.25.peg.5130 | hypothetical protein |
| 226 | scf_20796_88 | 1055 | 1525 | 471 | fig\|1465.25.peg.5141 | hypothetical protein |
| 227 | scf_20796_88 | 1750 | 1950 | 201 | fig\|1465.25.peg.5143 | hypothetical protein |
| 228 | scf_20796_88 | 813 | 1058 | 246 | fig\|1465.25.peg.5140 | hypothetical protein |

**C)**

| **No** | **Scaffold** | **Start position** | **End position** | **Length** | **ID** | **Function** |
| --- | --- | --- | --- | --- | --- | --- |
| 1 | cf_23640_103 | 308 | 457 | 150 | fig\|1465.23.peg.593 | hypothetical protein |
| 2 | scf_23640_111 | 278 | 394 | 117 | fig\|1465.23.peg.759 | hypothetical protein |
| 3 | scf_23640_123 | 170 | 292 | 123 | fig\|1465.23.peg.934 | hypothetical protein |
| 4 | scf_23640_12 | 108794 | 108916 | 123 | fig\|1465.23.peg.882 | hypothetical protein |
| 5 | scf_23640_12 | 109716 | 110630 | 915 | fig\|1465.23.peg.884 | hypothetical protein |
| 6 | scf_23640_12 | 121654 | 122058 | 405 | fig\|1465.23.peg.899 | Site-specific DNA recombinase |
| 7 | scf_23640_12 | 122046 | 123143 | 1098 | fig\|1465.23.peg.900 | Site-specific DNA recombinase |
| 8 | scf_23640_12 | 123146 | 123301 | 156 | fig\|1465.23.peg.901 | hypothetical protein |
| 9 | scf_23640_12 | 123451 | 124920 | 1470 | fig\|1465.23.peg.902 | hypothetical protein |
| 10 | scf_23640_12 | 125525 | 125758 | 234 | fig\|1465.23.peg.903 | hypothetical protein |
| 11 | scf_23640_12 | 125761 | 126138 | 378 | fig\|1465.23.peg.904 | hypothetical protein |
| 12 | scf_23640_12 | 126853 | 126990 | 138 | fig\|1465.23.peg.905 | hypothetical protein |
| 13 | scf_23640_12 | 128422 | 130458 | 2037 | fig\|1465.23.peg.907 | restriction enzyme BcgI alpha chain-like protein(EC:2.1.1.72); Ontology_term=KEGG_ENZYME:2.1.1.72 |
| 14 | scf_23640_12 | 130839 | 130967 | 129 | fig\|1465.23.peg.908 | hypothetical protein |
| 15 | scf_23640_12 | 132554 | 133273 | 720 | fig\|1465.23.peg.909 | hypothetical protein |
| 16 | scf_23640_12 | 133454 | 133615 | 162 | fig\|1465.23.peg.910 | hypothetical protein |
| 17 | scf_23640_12 | 133809 | 134948 | -1140 | fig\|1465.23.peg.911 | hypothetical protein |
| 18 | scf_23640_12 | 134938 | 135153 | -216 | fig\|1465.23.peg.912 | hypothetical protein |
| 19 | scf_23640_12 | 136578 | 136763 | -186 | fig\|1465.23.peg.914 | hypothetical protein |
| 20 | scf_23640_12 | 137105 | 137293 | -189 | fig\|1465.23.peg.916 | hypothetical protein |
| 21 | scf_23640_132 | 40 | 180 | -141 | fig\|1465.23.peg.1077 | hypothetical protein |
| 22 | scf_23640_1 | 100798 | 101715 | 918 | fig\|1465.23.peg.131 | phage protein |
| 23 | scf_23640_1 | 101753 | 102730 | 978 | fig\|1465.23.peg.132 | FIG01229679: hypothetical protein |
| 24 | scf_23640_1 | 102787 | 103266 | 480 | fig\|1465.23.peg.133 | phage protein |
| 25 | scf_23640_1 | 103281 | 104828 | 1548 | fig\|1465.23.peg.134 | Replicative DNA helicase (DnaB);  Ontology_t erm=KEGG_ENZYME:3.6.4.12 |
| 26 | scf_23640_1 | 105944 | 107635 | 1692 | fig\|1465.23.peg.136 | Single-stranded-DNA-specific exonuclease R ecJ; Ontology_term=KEGG_ENZYME:3.1.-.- |
| 27 | scf_23640_1 | 107652 | 111611 | 3960 | fig\|1465.23.peg.137; | DNA polymerase III alpha subunit; Ontology_term=KEGG_ENZYME:2.7.7.7 |
| 28 | scf_23640_1 | 113195 | 113551 | -357 | fig\|1465.23.peg.141 | hypothetical protein |
| 29 | scf_23640_1 | 116967 | 117119 | -153 | fig\|1465.23.peg.149 | hypothetical protein |
| 30 | scf_23640_1 | 119025 | 119252 | -228 | fig\|1465.23.peg.154 | hypothetical protein |
| 31 | scf_23640_1 | 122016 | 122546 | 531 | fig\|1465.23.peg.159 | Phage DNA modification methyltransferase |
| 32 | scf_23640_1 | 122581 | 122931 | -351 | fig\|1465.23.peg.160 | hypothetical protein |
| 33 | scf_23640_1 | 1233 | 1361 | 129 | fig\|1465.23.peg.3 | hypothetical protein |
| 34 | scf_23640_1 | 123472 | 123762 | -291 | fig\|1465.23.peg.162 | hypothetical protein |
| 35 | scf_23640_1 | 128992 | 129375 | 384 | fig\|1465.23.peg.175 | Ribonucleotide reduction protein NrdI |
| 36 | scf_23640_1 | 129347 | 131446 | 2100 | fig\|1465.23.peg.176 | Ribonucleotide reductase of class Ib (aerobic) alpha subunit; Ontology_term=KEGG_ENZYME:1.17.4.1 |
| 37 | scf_23640_1 | 131461 | 132453 | 993 | fig\|1465.23.peg.177 | Ribonucleotide reductase of class Ib (aerobic) beta subunit; Ontology_term=KEGG_ENZYME:1.17.4.1 |
| 38 | scf_23640_1 | 136269 | 136760 | -492 | fig\|1465.23.peg.184 | hypothetical protein |
| 39 | scf_23640_1 | 137104 | 137286 | -183 | fig\|1465.23.peg.185 | hypothetical protein |
| 40 | scf_23640_1 | 137329 | 138114 | 786 | fig\|1465.23.peg.186 | Phage protein |
| 41 | scf_23640_1 | 138367 | 138738 | -372 | fig\|1465.23.peg.188 | hypothetical protein |
| 42 | scf_23640_1 | 139390 | 139578 | -189 | fig\|1465.23.peg.191 | hypothetical protein |
| 43 | scf_23640_1 | 21462 | 22103 | 642 | fig\|1465.23.peg.23 | hypothetical protein |
| 44 | scf_23640_1 | 22116 | 23438 | 1323 | fig\|1465.23.peg.24 | hypothetical protein |
| 45 | scf_23640_1 | 23413 | 23982 | 570 | fig\|1465.23.peg.25 | Phage protein |
| 46 | scf_23640_1 | 24027 | 30200 | 6174 | fig\|1465.23.peg.26 | DNA double-strand break repair Rad50 ATPase |
| 47 | scf_23640_1 | 30347 | 31360 | 1014 | fig\|1465.23.peg.27 | prophage LambdaBa site-specific recombinase phage integrase family |
| 48 | scf_23640_1 | 31375 | 31791 | 417 | fig\|1465.23.peg.28 | Phage protein |
| 49 | scf_23640_1 | 31766 | 32257 | 492 | fig\|1465.23.peg.29 | hypothetical protein |
| 50 | scf_23640_1 | 32285 | 32719 | 435 | fig\|1465.23.peg.30 | hypothetical protein |
| 51 | scf_23640_1 | 32768 | 33148 | 381 | fig\|1465.23.peg.31 | hypothetical protein |
| 52 | scf_23640_1 | 33152 | 33424 | 273 | fig\|1465.23.peg.32 | hypothetical protein |
| 53 | scf_23640_1 | 33443 | 34207 | 765 | fig\|1465.23.peg.33 | Phage protein |
| 54 | scf_23640_1 | 34228 | 34965 | 738 | fig\|1465.23.peg.34 | hypothetical protein |
| 55 | scf_23640_1 | 34962 | 35180 | 219 | fig\|1465.23.peg.35 | hypothetical protein |
| 56 | scf_23640_1 | 35468 | 36679 | -1212 | fig\|1465.23.peg.36 | similar to (EMBL:AL117211) YPMT1.20C%2C Yersinia pestis CO-92 hypothetical protein from plasmid pMT1 similar to (EMBL:AF074611) Y1035 Yersinia pestis KIM5 hypothetical protein from plasmid pMT1 blastn and dotplot analyses suggest that an insertion event has taken place at this point as compared to Yersinia pestis pMT1 |
| 57 | scf_23640_1 | 36842 | 37243 | 402 | fig\|1465.23.peg.37 | Phage protein |
| 58 | scf_23640_1 | 37256 | 37738 | 483 | fig\|1465.23.peg.38 | Phage protein |
| 59 | scf_23640_1 | 37793 | 38791 | 999 | fig\|1465.23.peg.39 | phage virion protein |
| 60 | scf_23640_1 | 38821 | 39354 | 534 | fig\|1465.23.peg.40 | Phage protein |
| 61 | scf_23640_1 | 39387 | 40835 | 1449 | fig\|1465.23.peg.41 | phage virion protein |
| 62 | scf_23640_1 | 40853 | 42376 | 1524 | fig\|1465.23.peg.42 | FIG01229601: hypothetical protein |
| 63 | scf_23640_1. | 42388 | 44172 | 1785 | fig\|1465.23.peg.43 | prophage terminase, ATPase subunit, putative |
| 64 | scf_23640_1 | 44172 | 45095 | 924 | fig\|1465.23.peg.44 | unknown |
| 66 | scf_23640_1 | 4502 | 4639 | 138 | fig\|1465.23.peg.8 | hypothetical protein |
| 67 | scf_23640_1 | 45070 | 45303 | 234 | fig\|1465.23.peg.45 | hypothetical protein |
| 68 | scf_23640_1 | 45307 | 46503 | 1197 | fig\|1465.23.peg.46 | Phage protein |
| 69 | scf_23640_1 | 46515 | 46730 | 216 | fig\|1465.23.peg.47 | hypothetical protein |
| 70 | scf_23640_1 | 46989 | 47309 | -321 | fig\|1465.23.peg.48 | DNA-binding protein HBsu |
| 71 | scf_23640_1 | 47405 | 48961 | 1557 | fig\|1465.23.peg.49 | hypothetical protein |
| 72 | scf_23640_1 | 49088 | 50137 | 1050 | fig\|1465.23.peg.50 | unknown |
| 73 | scf_23640_1 | 50998 | 51228 | 231 | fig\|1465.23.peg.51 | hypothetical protein |
| 74 | scf_23640_1 | 51627 | 52166 | 540 | fig\|1465.23.peg.52 | hypothetical protein |
| 75 | scf_23640_1 | 52180 | 53412 | 1233 | fig\|1465.23.peg.53 | unknown |
| 76 | scf_23640_1 | 53658 | 54032 | 375 | fig\|1465.23.peg.54 | hypothetical protein |
| 77 | scf_23640_1 | 5432 | 5785 | 354 | fig\|1465.23.peg.10 | hypothetical protein |
| 78 | scf_23640_1 | 54686 | 54949 | 264 | fig\|1465.23.peg.55 | hypothetical protein |
| 79 | scf_23640_1 | 55342 | 55602 | 261 | fig\|1465.23.peg.56 | hypothetical protein |
| 80 | scf_23640_1 | 55735 | 57600 | 1866 | fig\|1465.23.peg.57 | hypothetical protein |
| 81 | scf_23640_1 | 57736 | 59340 | 1605 | fig\|1465.23.peg.58 | hypothetical protein |
| 82 | scf_23640_1 | 5862 | 6524 | 663 | fig\|1465.23.peg.11 | hypothetical protein |
| 83 | scf_23640_1 | 59391 | 59513 | 123 | fig\|1465.23.peg.59 | hypothetical protein |
| 84 | scf_23640_1 | 59563 | 61116 | 1554 | fig\|1465.23.peg.60 | Modification methylase HphIA; Ontology_term=KEGG_ENZYME:2.1.1.37 |
| 85 | scf_23640_1 | 61721 | 62356 | 636 | fig\|1465.23.peg.61 | hypothetical protein |
| 86 | scf_23640_1 | 62662 | 62901 | 240 | fig\|1465.23.peg.62 | hypothetical protein |
| 87 | scf_23640_1 | 63102 | 63392 | 291 | fig\|1465.23.peg.63 | hypothetical protein |
| 88 | scf_23640_1 | 63530 | 64546 | 1017 | fig\|1465.23.peg.64 | hypothetical protein |
| 89 | scf_23640_1 | 64819 | 65493 | 675 | fig\|1465.23.peg.65 | hypothetical protein |
| 90 | scf_23640_1 | 66011 | 66133 | 123 | fig\|1465.23.peg.66 | hypothetical protein |
| 91 | scf_23640_1 | 66143 | 66520 | 378 | fig\|1465.23.peg.67 | hypothetical protein |
| 92 | scf_23640_1 | 67154 | 67369 | 216 | fig\|1465.23.peg.68 | hypothetical protein |
| 93 | scf_23640_1 | 67468 | 67794 | 327 | fig\|1465.23.peg.69 | hypothetical protein |
| 94 | scf_23640_1 | 69127 | 69414 | 288 | fig\|1465.23.peg.71 | hypothetical protein |
| 95 | scf_23640_1 | 69414 | 70553 | 1140 | fig\|1465.23.peg.72 | GTPase domain, tubulin/FtsZ family protein |
| 96 | scf_23640_1 | 70705 | 71139 | 435 | fig\|1465.23.peg.73 | Programmed cell death toxin YdcE |
| 97 | scf_23640_1 | 71299 | 71556 | 258 | fig\|1465.23.peg.74 | hypothetical protein |
| 98 | scf_23640_1 | 71612 | 71824 | 213 | fig\|1465.23.peg.75 | hypothetical protein |
| 99 | scf_23640_1 | 71925 | 72590 | 666 | fig\|1465.23.peg.76 | Gll2498 protein |
| 100 | scf_23640_1 | 72618 | 72890 | 273 | fig\|1465.23.peg.77 | hypothetical protein |
| 101 | scf_23640_1 | 72928 | 73362 | 435 | fig\|1465.23.peg.78 | hypothetical protein |
| 102 | scf_23640_1 | 73400 | 73975 | 576 | fig\|1465.23.peg.79 | hypothetical protein |
| 103 | scf_23640_1 | 74011 | 74787 | 777 | fig\|1465.23.peg.80 | putative metal-dependent membrane protease |
| 104 | scf_23640_1 | 74807 | 75256 | 450 | fig\|1465.23.peg.81 | Phage protein |
| 105 | scf_23640_1 | 75877 | 76431 | 555 | fig\|1465.23.peg.82 | hypothetical protein |
| 106 | scf_23640_1 | 77181 | 77594 | 414 | fig\|1465.23.peg.83 | Guanosine-3-bis(Diphosphate) 3'-pyrophosphohydrolase;Ontology_term=KEGG_ENZYME:3.1.7.2 |
| 107 | scf_23640_1 | 77666 | 77896 | 231 | fig\|1465.23.peg.84 | hypothetical protein |
| 108 | scf_23640_1 | 77960 | 78148 | 189 | fig\|1465.23.peg.85 | hypothetical protein |
| 109 | scf_23640_1 | 78188 | 78478 | 291 | fig\|1465.23.peg.86 | hypothetical protein |
| 110 | scf_23640_1 | 78561 | 79163 | 603 | fig\|1465.23.peg.87 | Phage protein |
| 111 | scf_23640_1 | 91848 | 92414 | -567 | fig\|1465.23.peg.110 | hypothetical protein |
| 112 | scf_23640_1 | 93697 | 94005 | -309 | fig\|1465.23.peg.114 | hypothetical protein |
| 113 | scf_23640_1 | 94421 | 94567 | -147 | fig\|1465.23.peg.116 | hypothetical protein |
| 114 | scf_23640_1 | 96881 | 97309 | -429 | fig\|1465.23.peg.123 | hypothetical protein |
| 115 | scf_23640_1 | 97337 | 97567 | -231 | fig\|1465.23.peg.124 | hypothetical protein |
| 116 | scf_23640_1 | 98557 | 98817 | -261 | fig\|1465.23.peg.127 | hypothetical protein |
| 117 | scf_23640_21 | 74185 | 74826 | -642 | fig\|1465.23.peg.2130 | hypothetical protein |
| 118 | scf_23640_21 | 74849 | 75055 | -207 | fig\|1465.23.peg.2131 | hypothetical protein |
| 119 | scf_23640_22 | 1598 | 3454 | 1857 | fig\|1465.23.peg.2152 | FIG00642656: hypothetical protein |
| 120 | scf_23640_22 | 3441 | 3875 | -435 | fig\|1465.23.peg.2153 | hypothetical protein |
| 121 | scf_23640_22 | 6031 | 6291 | -261 | fig\|1465.23.peg.2156 | hypothetical protein |
| 122 | scf_23640_22 | 6405 | 7133 | -729 | fig\|1465.23.peg.2157 | hypothetical protein |
| 123 | scf_23640_22 | 7315 | 7512 | -198 | fig\|1465.23.peg.2158 | hypothetical protein |
| 124 | scf_23640_24 | 79710 | 80219 | -510 | fig\|1465.23.peg.2401 | mutT/nudix family protein |
| 125 | scf_23640_24 | 80560 | 80964 | -405 | fig\|1465.23.peg.2402 | hypothetical protein |
| 126 | scf_23640_24 | 81290 | 81439 | -150 | fig\|1465.23.peg.2403 | hypothetical protein |
| 127 | scf_23640_24 | 81617 | 81877 | -261 | fig\|1465.23.peg.2404 | hypothetical protein |
| 128 | scf_23640_24 | 82470 | 83282 | 813 | fig\|1465.23.peg.2406 | oxetanocin A resistance protein |
| 129 | scf_23640_25 | 23264 | 23434 | -171 | fig\|1465.23.peg.2432 | hypothetical protein |
| 130 | scf_23640_25 | 23504 | 23698 | -195 | fig\|1465.23.peg.2433 | hypothetical protein |
| 131 | scf_23640_25 | 23758 | 24276 | -519 | fig\|1465.23.peg.2434 | hypothetical protein |
| 132 | scf_23640_27 | 57773 | 58096 | -324 | fig\|1465.23.peg.2639 | hypothetical protein |
| 133 | scf_23640_27 | 60333 | 60485 | -153 | fig\|1465.23.peg.2644 | hypothetical protein |
| 134 | scf_23640_27 | 60488 | 60664 | -177 | fig\|1465.23.peg.2645 | hypothetical protein |
| 135 | scf_23640_27 | 60679 | 61392 | -714 | fig\|1465.23.peg.2646 | hypothetical protein |
| 136 | scf_23640_2 | 106035 | 106208 | -174 | fig\|1465.23.peg.1827 | hypothetical protein |
| 137 | scf_23640_2 | 106449 | 107123 | -675 | fig\|1465.23.peg.1828 | hypothetical protein |
| 138 | scf_23640_2 | 110699 | 110815 | -117 | fig\|1465.23.peg.1832 | hypothetical protein |
| 139 | scf_23640_2 | 110883 | 111359 | 477 | fig\|1465.23.peg.1833 | unknown |
| 140 | scf_23640_2 | 111352 | 111498 | -147 | fig\|1465.23.peg.1834 | hypothetical protein |
| 141 | scf_23640_3 | 208701 | 208913 | -213 | fig\|1465.23.peg.2994 | hypothetical protein |
| 142 | scf_23640_3 | 210336 | 211001 | -666 | fig\|1465.23.peg.2996 | hypothetical protein |
| 143 | scf_23640_3 | 211072 | 211740 | -669 | fig\|1465.23.peg.2997 | hypothetical protein |
| 144 | scf_23640_3 | 211733 | 212251 | -519 | fig\|1465.23.peg.2998 | hypothetical protein |
| 145 | scf_23640_3 | 212259 | 212375 | -117 | fig\|1465.23.peg.2999 | hypothetical protein |
| 146 | scf_23640_3 | 213086 | 213316 | -231 | fig\|1465.23.peg.3000 | hypothetical protein |
| 147 | scf_23640_3 | 213418 | 214695 | -1278 | fig\|1465.23.peg.3001 | hypothetical protein |
| 148 | scf_23640_3 | 214770 | 214901 | -132 | fig\|1465.23.peg.3002 | hypothetical protein |
| 149 | scf_23640_3 | 215068 | 215250 | -183 | fig\|1465.23.peg.3003 | hypothetical protein |
| 150 | scf_23640_3 | 215268 | 215630 | 363 | fig\|1465.23.peg.3004 | transcriptional regulator Cro/CI family |
| 151 | scf_23640_3 | 215809 | 216060 | -252 | fig\|1465.23.peg.3005 | hypothetical protein |
| 152 | scf_23640_3 | 216066 | 216335 | -270 | fig\|1465.23.peg.3006 | hypothetical protein |
| 153 | scf_23640_3 | 216384 | 216503 | -120 | fig\|1465.23.peg.3007 | hypothetical protein |
| 154 | scf_23640_3 | 216500 | 216736 | -237 | fig\|1465.23.peg.3008 | hypothetical protein |
| 155 | scf_23640_3 | 216874 | 217125 | -252 | fig\|1465.23.peg.3009 | hypothetical protein |
| 156 | scf_23640_3 | 217122 | 217331 | -210 | fig\|1465.23.peg.3010 | hypothetical protein |
| 157 | scf_23640_3 | 217444 | 217638 | -195 | fig\|1465.23.peg.3011 | hypothetical protein |
| 158 | scf_23640_3 | 217652 | 217948 | -297 | fig\|1465.23.peg.3012 | hypothetical protein |
| 159 | scf_23640_3 | 218091 | 218315 | -225 | fig\|1465.23.peg.3013 | hypothetical protein |
| 160 | scf_23640_3 | 218373 | 218600 | 0.0000000 | fig\|1465.23.pegsuperinfection exclusion protein.3014 | Transition state regulatory protein AbrB-0 |
| 161 | scf_23640_3 | 219239 | 219778 | -540 | fig\|1465.23.peg.3016 | hypothetical protein |
| 162 | scf_23640_3 | 219806 | 220171 | 366 | fig\|1465.23.peg.3017 | Ferric siderophore transport system%2C periplasmic binding protein TonB |
| 163 | scf_23640_3 | 220168 | 221352 | 1185 | fig\|1465.23.peg.3018 | Phage protein |
| 164 | scf_23640_3 | 221345 | 221596 | -252 | fig\|1465.23.peg.3019 | hypothetical protein |
| 165 | scf_23640_3 | 221580 | 222134 | 555 | fig\|1465.23.peg.3020 | Phage protein |
| 166 | scf_23640_3 | 222193 | 222705 | -513 | fig\|1465.23.peg.3021 | hypothetical protein |
| 167 | scf_23640_3 | 222698 | 224770 | 2073 | fig\|1465.23.peg.3022 | DNA polymerase I;Ontology_term=KEGG_ENZYM E:2.7.7.7 |
| 168 | scf_23640_3 | 224786 | 224977 | -192 | fig\|1465.23.peg.3023 | hypothetical protein |
| 169 | scf_23640_3 | 225085 | 225279 | -195 | fig\|1465.23.peg.3024 | hypothetical protein |
| 170 | scf_23640_3 | 225269 | 225613 | -345 | fig\|1465.23.peg.3025 | hypothetical protein |
| 171 | scf_23640_3 | 225610 | 225789 | -180 | fig\|1465.23.peg.3026 | hypothetical protein |
| 172 | scf_23640_3 | 225806 | 226006 | -201 | fig\|1465.23.peg.3027 | hypothetical protein |
| 173 | scf_23640_3 | 226040 | 226306 | -267 | fig\|1465.23.peg.3028 | hypothetical protein |
| 174 | scf_23640_3 | 226569 | 226892 | -324 | fig\|1465.23.peg.3029 | hypothetical protein |
| 175 | scf_23640_3 | 226889 | 227053 | -165 | fig\|1465.23.peg.3030 | hypothetical protein |
| 176 | scf_23640_3 | 227393 | 228538 | 1146 | fig\|1465.23.peg.3031 | RNA polymerase sporulation specific sigma factor SigG |
| 177 | scf_23640_3 | 228528 | 228644 | -117 | fig\|1465.23.peg.3032 | hypothetical protein |
| 178 | scf_23640_3 | 228812 | 229582 | -771 | fig\|1465.23.peg.3033 | hypothetical protein |
| 179 | scf_23640_3 | 229588 | 231951 | 2364 | fig\|1465.23.peg.3034 | DNA primase phage associated |
| 180 | scf_23640_3 | 232498 | 233883 | 1386 | fig\|1465.23.peg.3035 | DNA helicase phage-associated |
| 181 | scf_23640_3 | 233884 | 234120 | -237 | fig\|1465.23.peg.3036 | hypothetical protein |
| 182 | scf_23640_3 | 234148 | 234681 | -534 | fig\|1465.23.peg.3037 | hypothetical protein |
| 183 | scf_23640_3 | 235016 | 235876 | 861 | fig\|1465.23.peg.3038 | Site-specific recombinase XerD |
| 184 | scf_23640_3 | 235930 | 236586 | -657 | fig\|1465.23.peg.3039 | hypothetical protein |
| 185 | scf_23640_3 | 236583 | 238127 | 1545 | fig\|1465.23.peg.3040 | Mu-like prophage FluMu protein gp28 |
| 186 | scf_23640_3 | 238140 | 240311 | 2172 | fig\|1465.23.peg.3041 | Unknown |
| 187 | scf_23640_3 | 240385 | 243333 | -2949 | fig\|1465.23.peg.3042 | hypothetical protein |
| 188 | scf_23640_3 | 243346 | 243708 | -363 | fig\|1465.23.peg.3043 | hypothetical protein |
| 189 | scf_23640_3 | 243677 | 244204 | -528 | fig\|1465.23.peg.3044 | hypothetical protein |
| 190 | scf_23640_3 | 244204 | 244608 | -405 | fig\|1465.23.peg.3045 | hypothetical protein |
| 191 | scf_23640_3 | 244608 | 245165 | -558 | fig\|1465.23.peg.3046 | hypothetical protein |
| 192 | scf_23640_3 | 245339 | 245698 | -360 | fig\|1465.23.peg.3047 | hypothetical protein |
| 193 | scf_23640_3 | 245706 | 245927 | -222 | fig\|1465.23.peg.3048 | hypothetical protein |
| 194 | scf_23640_3 | 245940 | 247466 | -1527 | fig\|1465.23.peg.3049 | hypothetical protein |
| 195 | scf_23640_3 | 247466 | 247900 | -435 | fig\|1465.23.peg.3050 | hypothetical protein |
| 196 | scf_23640_3 | 247913 | 248260 | -348 | fig\|1465.23.peg.3051 | hypothetical protein |
| 197 | scf_23640_3 | 248269 | 248385 | -117 | fig\|1465.23.peg.3052 | hypothetical protein |
| 198 | scf_23640_3 | 248514 | 251393 | 2880 | fig\|1465.23.peg.3053 | Phage tail length tape-measure protein |
| 199 | scf_23640_3 | 251393 | 251989 | -597 | fig\|1465.23.peg.3054 | Putative phage protein |
| 200 | scf_23640_3 | 251982 | 253124 | -1143 | fig\|1465.23.peg.3055 | hypothetical protein |
| 201 | scf_23640_3 | 253121 | 253471 | -351 | fig\|1465.23.peg.3056 | hypothetical protein |
| 202 | scf_23640_3 | 253468 | 253854 | -387 | fig\|1465.23.peg.3057 | hypothetical protein |
| 203 | scf_23640_3 | 253871 | 254992 | 1122 | fig\|1465.23.peg.3058 | Phage-like element PBSX protein xkdT |
| 204 | scf_23640_3 | 255002 | 255580 | -579 | fig\|1465.23.peg.3059 | hypothetical protein |
| 205 | scf_23640_3 | 255565 | 255888 | -324 | fig\|1465.23.peg.3060 | hypothetical protein |
| 206 | scf_23640_3 | 255885 | 256280 | -396 | fig\|1465.23.peg.3061 | hypothetical protein |
| 207 | scf_23640_3 | 256297 | 258231 | -1935 | fig\|1465.23.peg.3062 | hypothetical protein |
| 208 | scf_23640_3 | 258251 | 258496 | -246 | fig\|1465.23.peg.3063 | hypothetical protein |
| 209 | scf_23640_3 | 258496 | 258618 | -123 | fig\|1465.23.peg.3064 | hypothetical protein |
| 210 | scf_23640_3 | 264698 | 264847 | -150 | fig\|1465.23.peg.3074 | hypothetical protein |
| 211 | scf_23640_3 | 264900 | 265094 | -195 | fig\|1465.23.peg.3075 | hypothetical protein |
| 212 | scf_23640_3 | 29426 | 29545 | -120 | fig\|1465.23.peg.2824 | hypothetical protein |
| 213 | scf_23640_3 | 29544 | 29660 | -117 | fig\|1465.23.peg.2825 | hypothetical protein |
| 214 | scf_23640_3 | 29643 | 29777 | -135 | fig\|1465.23.peg.2826 | hypothetical protein |
| 215 | scf_23640_3 | 31362 | 32531 | -1170 | fig\|1465.23.peg.2830 | hypothetical protein |
| 216 | scf_23640_47 | 35846 | 36034 | -189 | fig\|1465.23.peg.4143 | hypothetical protein |
| 217 | scf_23640_52 | 1459 | 1833 | -375 | fig\|1465.23.peg.4408 | Spore coat protein A |
| 218 | scf_23640_52 | 588 | 1130 | -543 | fig\|1465.23.peg.4407 | Spore coat protein A |
| 218 | scf_23640_55 | 10852 | 11565 | 714 | fig\|1465.23.peg.4473 | putative response regulator homolog of RumR and ScnR |
| 219 | scf_23640_55 | 12023 | 12373 | 351 | fig\|1465.23.peg.4474 | FIG01228292: hypothetical protein |
| 220 | scf_23640_55 | 12396 | 13127 | 732 | fig\|1465.23.peg.4475 | putative transporter trans-membrane domain bacteriocin immunity protein |
| 221 | scf_23640_55 | 13141 | 13893 | 753 | fig\|1465.23.peg.4476 | FIG01236037: hypothetical protein |
| 222 | scf_23640_55 | 13890 | 14807 | 918 | fig\|1465.23.peg.4477 | ABC transporter ATP-binding protein |
| 223 | scf_23640_55 | 15509 | 15805 | -297 | fig\|1465.23.peg.4478 | hypothetical protein |
| 224 | scf_23640_55 | 16348 | 16707 | -360 | fig\|1465.23.peg.4479 | hypothetical protein |
| 225 | scf_23640_55 | 16695 | 17282 | -588 | fig\|1465.23.peg.4480 | hypothetical protein |
| 226 | scf_23640_55 | 17599 | 18249 | -651 | fig\|1465.23.peg.4481 | hypothetical protein |
| 227 | scf_23640_55 | 8562 | 8906 | -345 | fig\|1465.23.peg.4470 | mutT/nudix family protein |
| 228 | scf_23640_55 | 8873 | 9070 | -198 | fig\|1465.23.peg.4471 | mutT/nudix family protein |
| 229 | scf_23640_55 | 9439 | 10851 | 1413 | fig\|1465.23.peg.4472 | putative histidine kinase ScnK homolog |
| 230 | scf_23640_58 | 10285 | 11100 | -816 | fig\|1465.23.peg.4516 | hypothetical protein |
| 231 | scf_23640_5 | 146 | 979 | -834 | fig\|1465.23.peg.4206 | hypothetical protein |
| 232 | scf_23640_5 | 15331 | 16230 | 900 | fig\|1465.23.peg.4219 | Peptidoglycan N-acetylglucosamine deacetylase;Ontology_term=KEGG_ENZYME:3.5.1.- |
| 233 | scf_23640_5 | 16804 | 16923 | -120 | fig\|1465.23.peg.4220 | hypothetical protein |
| 234 | scf_23640_5 | 17240 | 18562 | 1323 | fig\|1465.23.peg.4221 | peptidase S8 and S53 subtilisin kexin sedolisin |
| 235 | scf_23640_5 | 18516 | 18767 | -252 | fig\|1465.23.peg.4222 | hypothetical protein |
| 236 | scf_23640_5 | 18760 | 18900 | -141 | fig\|1465.23.peg.4223 | hypothetical protein |
| 237 | scf_23640_5 | 19014 | 19133 | -120 | fig\|1465.23.peg.4224 | hypothetical protein |
| 238 | scf_23640_5 | 19575 | 19739 | -165 | fig\|1465.23.peg.4225 | hypothetical protein |
| 239 | scf_23640_5 | 20362 | 20502 | -141 | fig\|1465.23.peg.4226 | hypothetical protein |
| 240 | scf_23640_5 | 209203 | 209466 | -264 | fig\|1465.23.peg.4341 | hypothetical protein |
| 241 | scf_23640_5 | 210116 | 211717 | 1602 | fig\|1465.23.peg.4342 | ATP-dependent DNA helicase UvrD/PcrA |
| 242 | scf_23640_5 | 211698 | 213599 | 1902 | fig\|1465.23.peg.4343 | FIG131328: Predicted ATP-dependent endonuclease of the OLD family |
| 243 | scf_23640_5 | 46089 | 46580 | -492 | fig\|1465.23.peg.4243 | hypothetical protein |
| 244 | scf_23640_5 | 46582 | 48510 | -1929 | fig\|1465.23.peg.4244 | plasmid-related protein |
| 245 | scf_23640_5 | 48620 | 48814 | -195 | fig\|1465.23.peg.4245 | hypothetical protein |
| 246 | scf_23640_5 | 52774 | 55368 | 2595 | fig\|1465.23.peg.4249 | L. lactis predicted coding region ORF00041 |
| 247 | scf_23640_5 | 55626 | 56627 | -1002 | fig\|1465.23.peg.4250 | hypothetical protein |
| 248 | scf_23640_5 | 57825 | 57962 | -138 | fig\|1465.23.peg.4252 | hypothetical protein |
| 249 | scf_23640_6 | 190697 | 190849 | -153 | fig\|1465.23.peg.4723 | hypothetical protein |
| 250 | scf_23640_6 | 190865 | 191191 | -327 | fig\|1465.23.peg.4724 | Prophage Lp1 protein |
| 251 | scf_23640_6 | 195404 | 195532 | -129 | fig\|1465.23.peg.4733 | hypothetical protein |
| 252 | scf_23640_6 | 202425 | 202583 | -159 | fig\|1465.23.peg.4747 | hypothetical protein |
| 253 | scf_23640_8 | 143018 | 145588 | -2571 | fig\|1465.23.peg.5097 | hypothetical protein |
| 254 | scf_23640_8 | 145979 | 146164 | -186 | fig\|1465.23.peg.5098 | hypothetical protein |
| 255 | scf_23640_8 | 146157 | 146276 | -120 | fig\|1465.23.peg.5099 | hypothetical protein |
| 256 | scf_23640_9 | 84161 | 84460 | -300 | fig\|1465.23.peg.5199 | hypothetical protein |
